# CRISPR/Cas9-mediated fine-tuning of miRNA expression in tetraploid potato

**DOI:** 10.1093/hr/uhac147

**Published:** 2022-06-30

**Authors:** Tjaša Lukan, Florian Veillet, Maja Križnik, Anna Coll, Tjaša Mahkovec Povalej, Karmen Pogačar, Katja Stare, Laura Chauvin, Jean-Eric Chauvin, Kristina Gruden

**Affiliations:** Department of Biotechnology and Systems Biology, National Institute of Biology, Večna pot 111, Ljubljana, 1000 Slovenia; IGEPP, INRAE, Institut Agro, Universite ´ de Rennes, Ploudaniel 29260, France; Department of Biotechnology and Systems Biology, National Institute of Biology, Večna pot 111, Ljubljana, 1000 Slovenia; Department of Biotechnology and Systems Biology, National Institute of Biology, Večna pot 111, Ljubljana, 1000 Slovenia; Department of Biotechnology and Systems Biology, National Institute of Biology, Večna pot 111, Ljubljana, 1000 Slovenia; Department of Biotechnology and Systems Biology, National Institute of Biology, Večna pot 111, Ljubljana, 1000 Slovenia; Department of Biotechnology and Systems Biology, National Institute of Biology, Večna pot 111, Ljubljana, 1000 Slovenia; IGEPP, INRAE, Institut Agro, Universite ´ de Rennes, Ploudaniel 29260, France; IGEPP, INRAE, Institut Agro, Universite ´ de Rennes, Ploudaniel 29260, France; Department of Biotechnology and Systems Biology, National Institute of Biology, Večna pot 111, Ljubljana, 1000 Slovenia

## Abstract

MicroRNAs (miRNAs) are small noncoding RNAs, which modulate the abundance and spatiotemporal accumulation of target mRNAs at transcriptional and post-transcriptional levels and through that play important roles in several biological processes in plants. Here we show that in polyploid species, CRISPR/Cas9 system can be used for fine-tuning of miRNA expression, which can have broader range of applications compared to knock-out mutants. We established the complete pipeline for CRISPR-Cas9-mediated modulation of miRNA expression in potato. It consists of (1) design and assembly of dual sgRNA CRISPR/Cas9 constructs, (2) transient transfection of protoplasts following fast and efficient screening by high resolution melting analysis to select functional sgRNAs, and (3) stable transformation of potato explants with functional sgRNAs and selection of regenerated transgenic lines with desired mutations and desired miRNA abundance based on sequencing and RT-qPCR. We show that miRNA-editing using dual sgRNA approach results in different types of mutations among transgenic lines but also in different alleles of the same plant, which are target site-dependent. The most frequent were short deletions, but we also detected 1-nt insertions (T or G), deletions between two sgRNAs and larger deletions. miRNA abundance correlates with the frequency and type of introduced mutations, as more extensive mutations in more alleles result in lower miRNA abundance. Interestingly, some mutated loci can generate alternative miRNAs, now novel targets were however predicted for those. In all transgenic lines with *Cas9* expression, we detected mutations, suggesting high efficiency of Cas9-editing. We confirmed the miRNA-editing efficiency of our optimised approach in two different potato genotypes and three different loci.

## Introduction

MicroRNAs (miRNAs) are 20- to 24-nucleotides-long endogenous small RNAs that modulate the abundance and spatiotemporal accumulation of target mRNAs at transcriptional and post-transcriptional levels and through that regulate several plant processes [29]. The biogenesis of miRNAs occurs in the nucleus where miRNA genes (*MIR*) are transcribed into long primary transcripts (pri-miRNAs), which undergo two slicing steps. The first slicing step results in the production of shorter stem-loop structures, called precursor miRNAs (pre-miRNAs), while in the second step double-stranded miRNA/miRNA^*^ duplexes are generated. miRNA/miRNA^*^ duplex consists of two potential mature miRNAs that derive from two ends (5′ and 3′) of the pre-miRNA precursor. Both mature miRNAs (termed as miR-5p and miR-3p) can be loaded into the Argonaute (AGO)-containing RNA-induced silencing complexes (RISC) to induce gene silencing through sequence-specific cleavage or translational repression of target mRNAs [25, 29]. In addition to the control of targets at the post-transcriptional level, miRNAs can also regulate gene expression on transcriptional level by inducing DNA methylation [58].

Fine-tuning miRNAs abundance is a powerful biotechnological strategy to improve performance of plants in the field [18]. For example, tolerance to abiotic or biotic stress in crops of economic importance can be adjusted using this approach [8]. The expression of small noncoding RNAs, including miRNAs, can be modulated by the CRISPR/Cas9 system as shown for some plant species [7, 64]. The novel gene-editing strategy is still a challenge, yet worth accepting, due to the compelling robustness, specificity, and stability for the modification of miRNA expression [4]. However, underlying molecular basis of CRISPR/Cas9 gene editing are still not well understood [8]. For example, the mechanisms of single guide RNA (sgRNA) interaction with the target and Cas9 protein is mechanistically not well understood, which frequently results in their inefficient design [60].

Cultivated potato (*Solanum tuberosum* L*.*) is one of the most important crops with yearly production of about 370 million tons [19]. Improving its resistance to biotic and abiotic stress is crucial to ensure high and environmentally friendly production. However, despite substantial breeding efforts for the development of diploid cultivars, potato is a tetraploid and largely heterozygous crop, which makes application of CRISPR/Cas9 technology even more challenging [39]. Not many studies on gene-editing in polyploids have been performed so far [48]. In potato, CRISPR/Cas9 technology was mostly used in combination with *Agrobacterium*-mediated stable transformation [10, 57, 64]. The alternative option is protoplast transfection [42], which is in potato problematic as regeneration of plants from protoplast is long lasting and difficult to establish for a specific genotype ([2, 3, 21, 55]).

In stable transformation, regeneration of transgenic plants is the time-consuming step. Therefore, to avoid stable transformations with poorly efficient sgRNAs, *in vivo* screening of sgRNA should be performed in advance to confirm their functionality [10, 28, 54]. A recent study showed that protoplast transfection with plasmid DNA is an efficient way to deliver CRISPR components into potato cells [22]. Thus, this seemed as a suitable approach for screening of sgRNA efficiency.

To date, CRISPR/Cas9-mediated miRNA editing in potato has not been reported. We established the complete pipeline for CRISPR-Cas9-mediated modulation of miRNA expression in potato, combining two methods for potato transformation: transient transfection of protoplasts and *Agrobacterium*-mediated stable transformation. The pipeline consists of design and assembly of dual sgRNA CRISPR-Cas9 constructs, transient transfection of protoplasts following screening by high resolution melting analysis to select functional sgRNAs, and stable transformation of potato with functional sgRNAs. In the last step, transgenic lines with desired mutations and miRNA abundance are selected by sequencing and RT-qPCR. We show that miRNA abundance correlates with the number and type of mutations. This is also in accordance with predictions of pre-miRNA processing efficiency, which is strongly affected in the same mutants. Therefore, we conclude that in polyploid species, one can use dual sgRNA CRISPR/Cas9 system for fine-tuning of miRNA expression, which may be even more useful than having only knock-out of function.

## Results

### High resolution melting analysis following protoplasts transfection is a fast and an efficient screening method for testing functionality of designed sgRNAs

**Figure 1:**
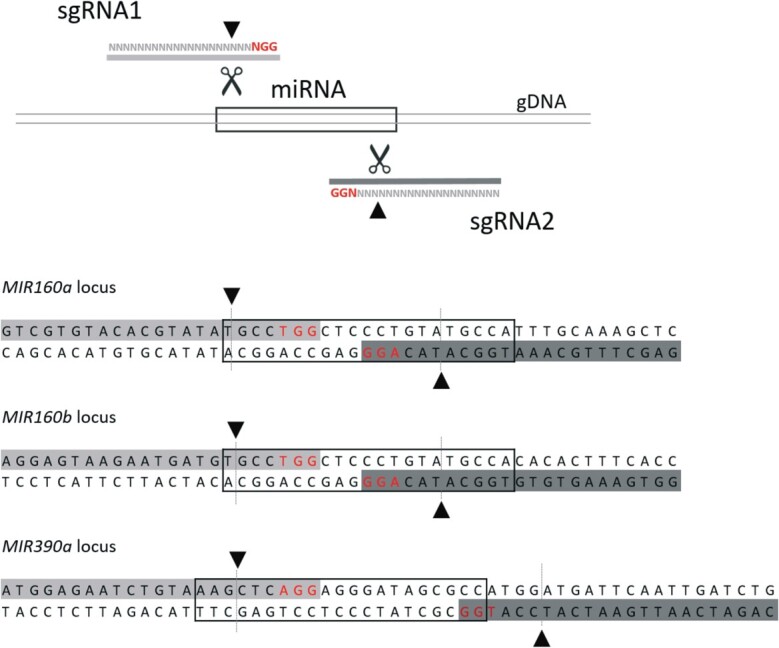
Design of dual single guide RNA (sgRNA) constructs for CRISPR-Cas9-mediated modulation of miRNAs expression. Two sgRNAs (sgRNA1 and sgRNA2) were designed for each target locus (*MIR160a*, *MIR160b* or *MIR390a*) to eliminate miR-5p (miR160a-5p, miR160b-5p and miR390-5p) produced at the loci. Insight from the genomic DNA (gDNA) of potato is given (bottom). Predicted cutting site of Cas9 is shown by arrows and dotted lines. PAM motif is shown in red. Sense and antisense strain of mature miRNA 5p coding sequence is boxed. sgRNA1 is shown in light grey while sgRNA2 is shown in dark grey.

To establish fast and efficient protocol for CRISPR/Cas9-mediated modulation of miRNA expression in potato, miR160a-5p, miR160b-5p and miR390a-5p, novel components of potato immune response which potentially regulate cell death and auxin signaling [27] and are encoded by three *MIR* genes (i.e. *MIR160a*, *MIR160b* and *MIR390a*), were chosen as targets. This way we were testing our protocol on unrelated miRNAs and had in addition also the ability to check specificity of the protocol, if related loci are present in the genome. Since miRNA silencing with a single sgRNA is not necessarily efficient due to the fact that the base frame shift does not always affect miRNA’s functionality [62], we designed sgRNAs to target both 5′ and 3′ ends of each mature miR-5p coding sequence (i.e. miR160a-5p, miR160b-5p, miR390a-5p). This should in optimal situation result in removal of whole miRNA sequence, which is the more abundant of the two produced from the same loci, or at least sufficiently perturb the structure of pre-miRNA to impair proper excision of miRNAs (Figure 1, Figure S1, Figure S2).

**Figure 2:**
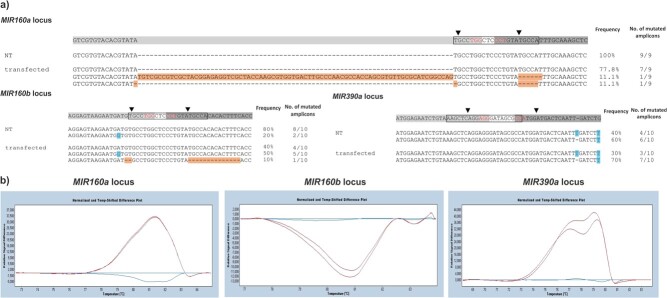
Confirmation of the functionality of designed single guide RNAs (sgRNAs) by protoplasts transfection. a) Diversity of mutations observed in CRISPR/Cas9-edited regions of *MIR* genes in transfected protoplasts. Results of Sanger sequencing for individual PCR products within *MIR* genes to identify types and frequency of mutations in CRISPR-edited protoplasts of potato cv. Désirée (bottom; transfected) and natural polymorphism in control potato cv. Désirée (top; NT) are shown. Next to sequences, the percentage of PCR product with the particular type of mutations is shown. Mature miRNA 5p (boxed), sgRNA1 (light grey), sgRNA2 (dark grey), PAM motif (red), theoretical cutting site (arrowhead) as located in the precursor sequences are shown above the alignment. Allelic variations are shown in blue, mutations are shown in orange. b) Presence of mutations in target loci as determined by high resolution melt analysis (HRM). HRM was performed on two technical replicates of PCR amplification for each target loci (*MIR160a*, *MIR160b* and *MIR390a*), using genomic DNA isolated from protoplasts from three independent transfections pooled together as a template. Melting curves of the PCR products are shown (see Figure S3 for HRM primer design). Red lines: transfected protoplasts, blue lines: non-transfected protoplasts.

To streamline the process, the functionalities of designed sgRNAs were tested in a transient set up prior stable transformation. For each target miRNA, both sgRNAs were simultaneously introduced into potato protoplasts of cv. Désirée using a vector set for multigene cloning and expression in potato [11]. At the stage of first protoplasts divisions, mutations in transfected protoplasts were detected by the high resolution melting analysis (HRM) for all *MIR* loci targets (Figure 2a), as the melting curves differed from the melting curves of the control (non-transfected protoplasts). Besides, the melting curves differed also between the replicates, suggesting the presence of different types of mutations (Figure 2a).

To confirm the results of HRM and further determine the frequency and types of mutations in miRNAs in transfected protoplasts, individual PCR products covering mature miRNAs and adjacent regions were analysed by sequencing (Data S1). First, we checked for natural allelic variation within the Désirée genotype we studied. We detected allelic variation in miRNAs coding regions and sgRNA target regions in control samples for *MIR160b* and *MIR390a*, but not for *MIR160a* (Figure 2b, “polymorphism in NT protoplasts” sheet in Data S1 and Figure S4). Next, we analysed the same regions in transfected potato protoplasts to search for potential CRISPR-mediated mutations. The results confirmed the presence of mutations in *MIR160a* and *MIR160b*, while we did not detect any mutations in *MIR390a* (Figure 2b, “type of mutations” sheet in Data S1 and Figure S5). The discrepancy between the results obtained by each methodology could be explained by low editing efficiency of designed sgRNAs and a relatively low number (10) of sequenced individual PCR products.

### miRNA-editing using dual sgRNA constructs results in diverse types of mutations in stably transformed potato plants

**Figure 3:**
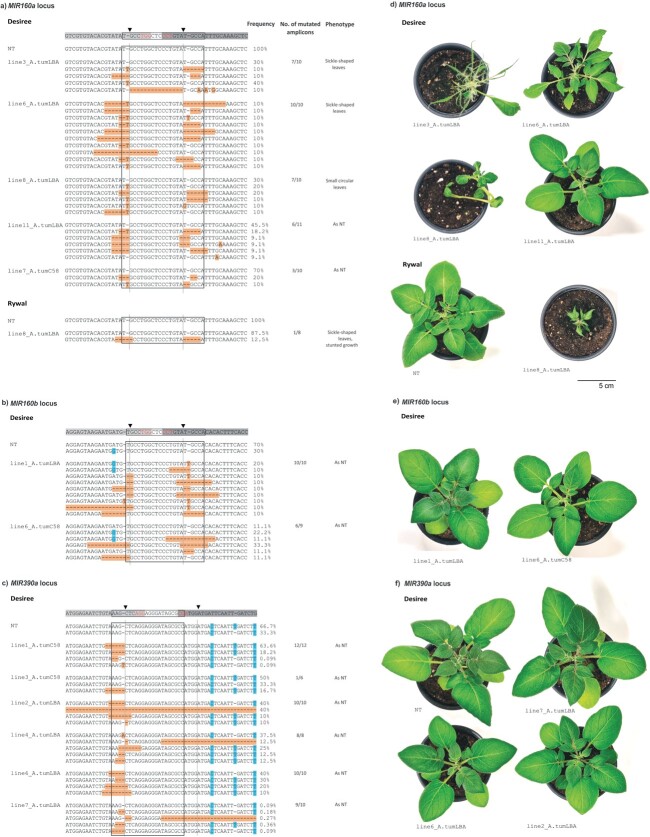
Diversity of mutations observed in CRISPR/Cas9-edited *MIR* loci in stably transformed plants. Results of sequencing for individual PCR products within a) *MIR160a* in Désirée and Rywal, b) *MIR160b* in Désirée and c) *MIR390a* in Désirée genotypes. For each transformation different transgenic lines were analysed (denoted as lines in sequence alignments; each line is named with a number and the Agrobacteria strain used for transformation) and within each line individual PCR products were sequenced (several sequences per each line are given). Natural sequence variability in control potato cv. Désirée and cv. Rywal (NT) is also shown. Next to sequences, the percentage of PCR products with the particular type of mutations is shown. See Figure S6 for the number of PCR products sequenced and Data S2 and Data S3 for sequencing IDs, primers used and types of mutations. Mature miR-5p (boxed), sgRNA1 (light grey), sgRNA2 (dark grey), PAM motif (red), theoretical cutting site (arrowhead) as located in the precursor are shown above the alignment. Allelic variations are shown in blue, mutations are shown in orange. Phenotypes of three weeks old d) cr-*MIR160a* and Rywal NT, e) cr-*MIR160b* and f) cr-*MIR390a* and Désirée NT plants grown in soil are shown. Scale is 5 cm.

Since all sgRNA constructs were confirmed as functional by HRM in the protoplast transient expression system, we proceeded with stable transformation of two potato genotypes, cv. Désirée and cv. Rywal, using two different strains of *Agrobacterium,* LBA4404 and C58pMP90. For each target *MIR loci*, both sgRNAs were simultaneously introduced into potato explants, using a vector set for multigene cloning and expression in potato, to obtain transgenic plants [11]. We observed no change in phenotype in CRISPR-edited *MIR390a* (*cr-MIR390a*) and *MIR160b* (*cr-MIR160b*) transgenic lines grown in tissue cultures or soil if compared to non-transgenic plants. On the other hand, some of the CRISPR-edited *MIR160a* (*cr-MIR160a*) transgenic lines (40% (2/5) in case of cv. Désirée (line3_A.tumLBA, line6_A.tumLBA) and 100% (1/1) in case of cv. Rywal) had sickle-shaped leaves and exhibit lamina growth arrest (Figure 3d,e,fData S2, Data S3) when grown in tissue cultures or soil. This phenotype was expected, as it is typical for plants with perturbed auxin signalling [51] as auxin response factors (ARFs) are known targets of miR160-5p. Besides, one Désirée transgenic line had small circular leaves when grown in soil (line8_A. tumLBA; Figure 3d,e,f).

To search for transgenic lines with introduced CRISPR-mediated mutations in specific *MIR* loci, we sequenced PCR products covering mature miR-5p coding regions and adjacent regions directly and analysed them using Inference of CRISPR Edits (ICE) Analysis Tool [23]. Transgenic lines with the highest knock-out score, which is determined by the rate of editing at a particular locus, and the lines with different phenotype (sickle-shaped leaves), were further analysed by cloning of PCR products and sequencing of individual products to determine the types of mutations and their frequency (see “types of mutations” sheet in Data S2, Data S3 and Figure S6; https://doi.org/10.5281/zenodo.5727015). To exclude the possibility that detected mutations in the analysed regions are natural polymorphism (SNPs), we determined allelic variation in analysed regions in potato cv. Rywal and cv. Désirée (see Figure 3a,b,c; “polymorphism in NT plants” sheet in Data S2, Data S3 and Figure S6).

We obtained several different types of mutations detected in different transgenic lines but also in different alleles of the same plant. For all *MIR loci*, the most abundant types of mutations were short deletions (up to 6 nt), but we also identified 1-nt insertions (T or G) and longer deletions (Figure 3a,b,c; Data S2). Mutations were frequently, but not exclusively introduced in the expected (a few nucleotides upstream of PAM motif) cutting site (Figure 3a,b,c). Only in one transgenic line, cr-*MIR160a*_line3_A.tumLBA, we detected excision of the complete region in-between expected cutting site of both sgRNAs (Figure 3a).

In the majority of the cr-*MIR160a* Désirée transgenic lines, at least one of the alleles remained non-mutated (Figure 3a,b,c; Data S2). However, we also obtained one transgenic line, cr-*MIR160*a_line6_A.tumLBA, in which all alleles analysed were modified (10/10 mutated amplicons; Data S2). In contrast, in half of the cr-*MIR160b* and cr-*MIR390a* transgenic lines, only modified alleles were detected (Figure 3a,b,c; Data S2). Of note, transformation efficiency as well as editing efficiency were much lower in cv. Rywal, as we managed to obtain one cr-*MIR160a* Rywal transgenic line with one mutated allele detected (Figure 3a,b,c; Data S3).

In case of cv. Désirée, both *Agrobacterium* strains were functional, although the percentage of transgenic lines with mutations was higher when LBA4404 strain was used (80% (4/5 plants) vs. 20% (1/5) for *MIR160*a, 50% (1/2) vs. 33% (1/3) for *MIR160b* and 80% (4/5) vs. 40% (2/5) for *MIR390a*) (Figure 3a,b,c) compared to C58pMP90. Similarly, in cv. Rywal, we produced one transgenic line with LBA4404 strain, while we did not produce any transgenics when C58pmp90 *Agrobacterium* strain was used.

To exclude the possibility of off-target effects in the homologous regions (55% identity in the target region), we also confirmed that the construct for editing *MIR160a* did not introduce mutations in *MIR160b* loci and the construct for editing *MIR160b* did not introduce mutations in *MIR160a* loci (see “off-targets effect” sheet in Data S2).

### miRNA abundance correlates with the number and type of introduced mutations in potato

**Figure 4:**
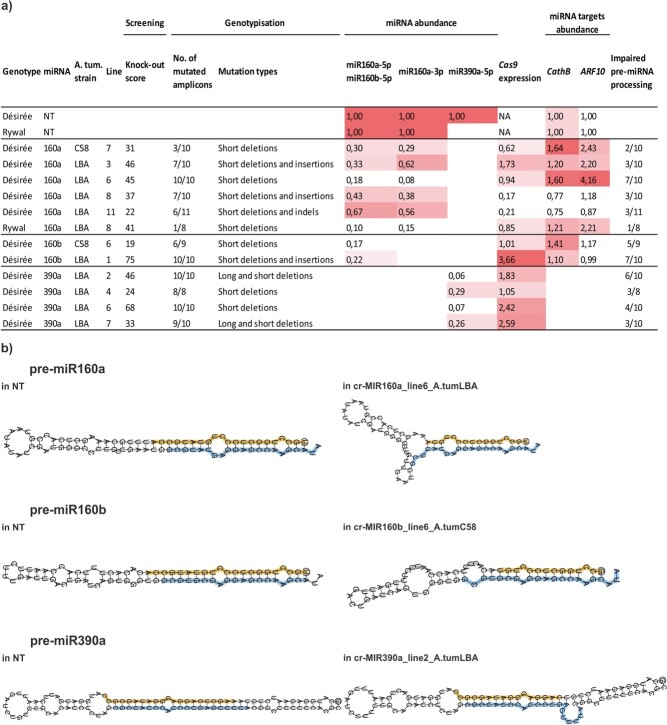
miRNA abundance correlates with the number and type of CRISPR-induced mutations in potato. a) To link the type and frequency of mutations with miRNA abundance, we followed relative miRNA abundance in the selected transgenic lines. Relative miRNA abundance in transgenic lines normalised to the averaged expression in non-transgenics (NT) is given. Values are shaded in red according to their expression level. We also checked, if the construct was fully inserted into the genome of each transgenic line and determined expression of *Cas9* gene. Genotype: potato genotype Désirée or Rywal used for stable transformation, A. tum. strain: *Agrobacterium tumefaciens* strain LBA4404 or C58pMP90 used for stable transformation of potato, miRNA: target miR160a-5p, miR160b-5p or miR390a-5p, Line: potato transgenic line, Screening (knock-out score): determined by the rate of editing at a particular locus) according to CRISPR Edits (ICE) analysis (Synthego; see methods), Genotypisation: results of sequencing of individual PCR products (number of mutated PCR products divided by all analysed PCR products and mutation types in mutated PCR products), miRNA abundance: relative abundance of mature miR160a-5p, miR160b-5p, miR160a-3p and miR390a-5p determined by RT-qPCR, *Cas9* expression: relative expression of *Cas9* in transformed plants determined by RT-qPCR. NA: not applicable. miRNA targets abundance: relative expression of *CathB* and *ARF10* in transformed plants determined by RT-qPCR. Impaired pre-miRNA processing: pre-miRNAs processing efficiency has been predicted as impaired if the MFEI values of pre-miRNAs were below 0.85 (see methods) or internal loop (> 5 nt) has been detected in miRNA/miRNA^*^ duplex region. Indicated are the number of pre-miRNAs with impaired processing compared to the number of all PCR products analysed in each line. b) Examples of perturbed secondary structures of pre-miRNA sequences. One example is given per each selected potato *MIR loci*, secondary structure of non-mutated pre-miRNA is shown for comparison, see https://doi.org/10.5281/zenodo.5727015 for all. Sequences of miRNAs coding regions are highlighted in colours (miR-5p – orange, miR-3p - blue).

To determine whether the type and frequency of mutations has an effect on miRNA abundance, we analysed miRNA abundance levels in the selected transgenic lines (Figure 4a; Data S4). As a control, we determined baseline miRNA expression in NT plants. As expected, miRNA abundance was lower in the transgenic lines with more alleles modified (cr-*MIR390a*_line2_A.tumLBA vs. cr-*MIR390a*_line4_A.tumLBA and cr-*MIR160a*_line6_A.tumLBA vs. cr-*MIR160a*_line3_A.tumLBA). Interestingly, in six transgenic lines with mutations in all analysed amplicons, the expression of miRNA was still detected, although its level was greatly reduced (Figure 4a). One explanation could be that some types of mutations, especially single point mutations (observed in one allele of *MIR390a*_line2) do not necessarily lead to disruption of the secondary structure of precursor and consequently to disruption of DCL processing activity to produce mature miRNA [13, 47].

Therefore, to investigate the effects of the introduced mutations, we predicted secondary structures for all altered pre-miRNAs of each transgenic line and assessed their stability and potential for efficient processing of miRNAs from these precursors (Data S2, Data S3, https://doi.org/10.5281/zenodo.5727015). Minimal folding free energy (MFE) as a measure of folding stability has been one of the main criteria for accurate miRNA annotation. Plant miRNA precursors however exhibit AU bias, that is absent in other non-coding RNAs [61]. Therefore, Zhang *et al.* proposed the minimal folding free energy index (MFEI>0.85) of pre-miRNAs, that considers this, as a better criterion for distinguishing pre-miRNAs from other RNAs. By calculating MFEI values we observed that 27 out of 52 mutated pre-miRNAs did not satisfy the criterion of MFEI values above 0.85, but instead showed values more similar to tRNA, rRNAs and mRNAs (MFEI values ~0.6) [61]. The vast majority (24) of these 27 altered pre-miRNAs harbour 4-nt or longer deletions and therefore exhibit highly altered secondary structures (Data S2, Data S3, https://doi.org/10.5281/zenodo.5727015) with reduced stability, which may affect DCL1 recognition and processing efficiency. Additionally, changes in the local structural features, such as the position of bulges and internal loops and their shape have been shown to reduce the processing efficiency and accuracy [49, 65]. Typical miRNA/miRNA^*^ duplexes should have five or fewer mismatches [5] as higher number of mismatches would lead to destabilization of duplexes [9]. Internal loops can change the initial positions of DCL cleavage sites and impair the production of mature miRNAs [49]. By examining miRNA/miRNA^*^ duplex region of altered pre-miRNAs (MFEI<0.85), we uncovered 17 pre-miRNAs in which internal loops were formed in duplex region due to an increased number of mismatches (> 5 nts), which were the result of CRISPR-Cas9 induced deletions. Altogether we predict that 28 out of 52 mutated pre-miRNAs cannot be processed efficiently, including those with 126 nt-long deletion, which resulted in whole pre-miRNA excision (Figure 4a, Data S2, Data S3). However, some altered pre-miRNAs might still be processed due to retention of a stable structure or due to the absence of internal loops in duplex regions (Data S2).

### CRISPR-Cas9 introduced mutations can induce generation of new miRNA variants

**Figure 5:**
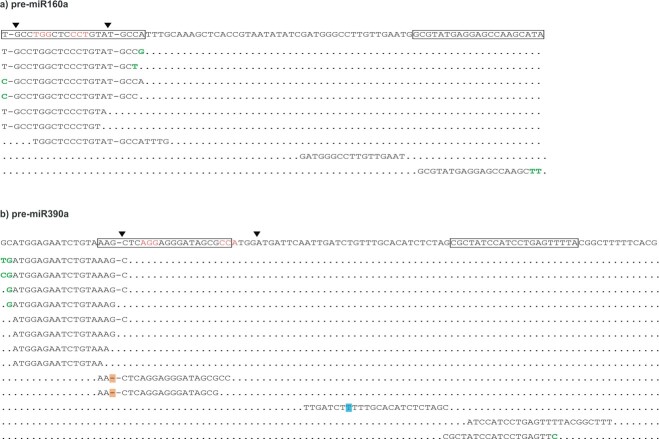
miRNA variants detected in cr-*MIR160a* and cr-*MIR390a* transgenic lines. Nine miRNA variants originating from mutated *MIR160a* locus (a) and thirtheen miRNA variants originating from mutated *MIR390a* locus (b) aligned to wild-type pre-miRNA sequences. Mature miR-5p and miR-3p (boxed), PAM motif (red), Cas9 theoretical cutting site (arrowhead) as located precursor are shown above the alignment. Allelic variations are shown in blue, mutations are shown in orange, post-transcriptional modifications are highlighted in green.

Using sRNA sequencing approach, we experimentally confirmed whether CRISPR-Cas9 induced mutations affected processing or instead provide DCL with a new template to generate new miRNA variants from the mutated pre-miRNA precursors. For sequencing, we selected three cr-*MIR160a* and three cr-*MIR390a* transgenic lines harbouring the most diverse types of mutations in pre-miRNA precursors (Figure S6). miRNA variants specifically produced from mutated pre-miRNAs were identified by mapping sRNA reads to mutated pre-miRNAs, excluding those existing in NT plants (Figure S7). By mapping sRNA reads to precursors, we identified only templated miRNA variants, i.e. those that exhibit perfect sequence complementarity to their pre-miRNAs and vary in length [41]. Since miRNA variants can also be generated by post-transcriptional modifications, through enzymatic activity that either adds or removes specific nucleotides at the miRNA ends, we ran the isomiR identification pipeline to also identify these so-called non-templated miRNA variants to account for the potentially induced generation of new miRNA variants by CRISPR-Cas9 [41]. By using a high threshold filter for quality of sRNA sequences, we ensured that all the sequences we identified as miRNA variants, specifically generated from transgenic lines were genuine. In total, we identified 9 miRNA variants in cr-*MIR160a* and 13 miRNA variants in cr-*MIR390a* lines mainly originating from 5’end of pre-miRNAs, which corresponds to the site of CRISPR-Cas9 induced mutations (Figure 5, Figure S8). Almost all were of low abundance, being represented by only one or two read counts (Figure S8, Data S5). Those that could be unambiguously assigned (mapped only to one specific altered pre-miRNAs, marked with asterisks, see Figure S8), corresponded to three pre-miRNA precursors of cr-*MIR160a* and three of cr-*MIR390a* transgenic lines, having slightly altered secondary structure, for which we predicted that introduced mutations would still allow their processing. This shows that our predictions agree well with the experimental data (Figure S8, Data S2). For two altered pre-miRNAs, one from cr-*MIR160a*_line3_A.tumLBA and one from cr-*MIR390a*_line2_A.tumLBA, where miRNA expression was still detected even though all alleles analysed were altered, we found that 1-nt indels in these two precursors did not affect pre-miRNA processing (Figure S6, Figure S8). We identified a shorter variant of miR390-5p in these plants. For cr-*MIR390a_*line6_A.tumLBA (also all alleles modified), no novel miRNA variants were detected, although the results of our prediction indicated that 7 out of 10 pre-miRNAs could still be processed, however it seems that 2-nt long deletions were sufficient to impair the processing (Figure S8, Data S2). Interesting example is mutated pre-miRNAs from cr-*MIR390a_*line7_A.tumLBA with long deletion, resulting in nearly half of the length of wild-type pre-miR390a (59 nt instead of 108 nt), for which impaired processing was predicted. In these plants several novel miRNA variants were discovered. Two miRNA variants produced from this precursor have the highest abundance among detected novel miRNA variants, comparable to the levels of the corresponding canonical miRNAs in NT plants, providing further evidence that processing was not impaired in this case, but it yielded functionally unrelated miRNAs (Data S5).

### miRNA variants produced from mutated *MIR* loci are not functionally relevant

To evaluate the effect of mutations in the 5p coding region of mature miRNAs in terms of conservation of biological function, all detected novel miRNA variants originating from altered precursors were subjected to target prediction screening using the psRNATarget tool to assess binding to their corresponding wild-type miRNA targets. In potato, miR160a-5p and miR160b-5p guide the cleavage of *ARF10* and *ARF17*, whereas miR390a-5p directs cleavage of non-coding *TAS3* transcripts [27, 40, 59], leading to the production of *TAS3*-tasiRNAs that negatively regulate *ARF2*, *ARF3* and *ARF4* mRNAs [1]. Target analysis revealed that 5 out of 9 variants of cr-*MIR160a* lines retained the target binding to *ARF10* and *ARF17* (Data S6), but all were detected in minute quantities (Data S5). The miRNA variant cr-miR160a.7 harbour four mismatches, while the remaining variants have up to two mismatches compared to wild-type miR160a-5p. Of the 13 miRNA variants of cr-*MIR390a*, only cr-miR390a.5 with one nucleotide deletion has been detected to preserve binding to *TAS3* (Data S6). Next, we searched for potential novel targets of two highly abundant variants detected in cr-*MIR390a_*line7_A.tumLBA that had comparable abundance to mature miRNAs in wild-type samples. No target transcripts were found in potato for a miRNA variant cr-miR390.8, whereas the variant cr-miR390a.12 was predicted to target *GCN5-related N-acetyltransferase*. Upon closer examination of the target prediction results, we detected three consecutive mispairings between 12–14 nt of miRNA sequence, (Data S6) corresponding to the central miRNA region. Base pairing here is critical for miRNA-mediated target repression so miRNA may not be functionally relevant [30].

To assess the impact of reduced levels of mature miRNAs due to CRISPR-Cas9 induced mutations, along with the fact that also some miRNA variants can also maintain target binding, we measured the expression levels of miRNA targets in the selected transgenic lines. We found that the expression *of ARF10*, target of miR160a-5p and miR160b-5p and expression of *CathB*, target of miR160a-3p, are negatively correlated with miRNA abundance. This shows that CRISPR-Cas9 induced mutations efficiently reduce mature miRNAs levels and that five miRNA variants which were predicted to retain target binding, do not contribute to the regulation of the mature miRNAs targets, which is also consistent with their low abundance (Figure 4a, Data S6, Data S5).

### miRNA-editing efficiency with selected dual sgRNA constructs is high

To determine editing efficiency, the presence of the transgene in the genome of transgenic lines was first confirmed by amplifying several regions of T-DNA (Cas9, antibiotic selection and sgRNAs) in the genome of transgenics with mutations (Figure S9). Next, we followed *Cas9* expression. In all transgenic lines with *Cas9* expression, we detected mutations, suggesting high efficiency of Cas9-editing. We conclude that our protocol for CRISPR-editing is highly efficient, as T-DNA was integrated, *Cas9* was expressed and was functional in all transgenic lines (Figure 4).

## Discussion

We developed a protocol for fast and efficient CRISPR/Cas9-mediated miRNA expression modulation in tetraploid plant. The protocol consists of three steps: 1) design and assembly of dual sgRNAs into plant expression plasmids, 2) transient transfection of protoplasts followed by HRM to select functional sgRNAs and 3) stable transformation of potato with functional sgRNAs followed by the selection of transgenic lines with desired mutations and miRNA abundance by sequencing and qPCR.

We choose experimental design of dual sgRNAs bordering miRNA-5p excision site. Using such approach, we can expect also excision of sequences in between both cutting sites besides the indels generated by individual sgRNAs. Following protoplasts transfection, functionalities of sgRNAs were determined by HRM and Sanger sequencing. We were able to confirm mutated alleles in *MIR160a* and *MIR160b* by both methods (Data S1). The presence of mutations could in *MIR390a*-edited protoplasts only be confirmed by HRM. This could be explained by low editing efficiency of designed sgRNAs and relatively low number of analysed individual PCR products (Data S1) as following stable transformation we did obtain mutated lines with the same set of sgRNAs. Determined by sequencing, low editing efficiency in potato protoplasts transfection set-up was observed in our experiment (20% and 10% in case of *MIR160a* and *MIR160b*, respectively), but was also shown by others [2, 52]. Therefore, we conclude that HRM is fast and reliable screening method for the selection of functional sgRNAs and additional sequencing is not required at this step of the procedure.

According to the literature, Cas9 generates a DNA double-strand break at a position about 3 bp upstream of the PAM sequence. We detected different types of short deletions at this position (Figure 2, Figure 3, Data S1, Data S2), which is in accordance with other studies reporting CRISPR-mediated editing of different potato genes [10, 54, 57, 64] as well as CRISPR-mediated miRNA editing in rice [12], soybean [24] and tomato [15]. In case of cr-*MIR160a* Désirée transgenic lines, mutations were frequently, but not exclusively introduced a few nucleotides upstream of PAM motif (Figure 3). In accordance with our study, the mutations were frequently present close to the cutting site also in other studies of potato gene editing, at positions 2–5 bp upstream or 4 nt downstream of PAM motif [2], 3 or 4 nt upstream of PAM [54], 0–4 nt upstream of PAM [10] and 3 nt upstream or 0 nt downstream of PAM [57].

Deletion of the whole fragment between two target regions was observed in one line (cr-*MIR160a_*line3_A.tumLBA, cv. Désirée) (Figure 3). Although due to the dual sgRNA strategy that we applied, we expected to obtain more transgenic lines with deletions between target sites, efficiency of dual sgRNA approach is most probably sequence- and species-dependent, according to the results of several other studies where deletions between two guide sequences appeared with different frequencies [44, 52, 56]. Besides, it is also highly dependent on the sgRNAs cutting efficiencies, as simultaneous cutting at both target sites is crucial to get the deletion. On the other hand, we showed that individual cutting by both sgRNAs, resulting in short deletions or 1 nt insertions in both target sites, is high, as it was detected in all transgenic lines for *MIR160a* and *MIR160b* constructs and in 50% of transgenic lines for *MIR390a* construct (Figure 3). Since miRNAs abundance was reduced in all transgenic lines, we conclude that deletion of the whole miRNA coding region is desired, but not crucial to decrease miRNA abundance (Figure 3).

We have however shown, both through *in silico* predictions and sRNA sequencing (Figure 4, Figure 5), that single nuclotide deletions and insertions do not always impair the processing of pre-miRNA. We detected miRNA variants in such cases. This is consistent with the study by Cuperus *et al.*, who reported that even just a point mutation in the pre-miRNA region can affect processing by DCL1, leading to increased accumulation of 22-nt miRNAs instead of the wild-type 21-nt form, along with the generation of size heterogeneous miRNA sequences (~20–24 nt) [13]. We predicted that these miRNA variants can still target the same transcripts (Data S6). Similarly, 1 bp-indels in mature miR528 region did not abolish miRNA function in rice [64]. The quantity of these newly generated miRNA variants was however much lower compared to the wild-type miRNAs (Data S5), indicating lower efficiency of precursor processing also in case of short indels.

By analysing the phenotype of transgenic plants, we observed that *MIR160a*-edited plants with lower miR160a abundance have sickle-shaped leaves and exhibit lamina growth arrest (Figure 3, Figure 4). This is in accordance with the results published by Damodharan *et al.* [15], who observed abnormal leaf phenotypes in miR160a-edited tomato plants. The phenotype of *MIR160b*-edited plants did not change due to the dominant role of *MIR160a* loci over *MIR160b*. *MIR160a* loci is not affected in cr-*MIR160b*-edited plants and since the identical miRNA (miR160a-5p) is produced from *MIR160a* loci this does not affect the final phenotype. Similar observations have been discovered in the study by [15], who observed no difference in phenotypes of *MIR160b*-edited and wild-type tomato plants, further indicating a dominant role of *MIR160a* over *MIR160b* in tomato.

We obtained transgenic lines with different types of mutations in different alleles of the same plant (Figure 3). This is in accordance with results of the *OsMIR818b* gene editing in rice reported by [12], where 39% of transgenic lines had heterogeneous mutations. Moreover, more than four different sequences were observed for a single plant (Figure 3), suggesting that these transgenic lines are mosaic. Our results are in accordance with several other studies, which demonstrate that the occurrence of mosaic plants is frequently high in primary transformants [20, 43, 45, 52, 54].

Editing efficiency to mutate all four alleles in miRNA coding region is target-dependent. In our study, all sequences were mutated in 20% of analysed transgenic lines of cv. Désirée for *MIR160a*, 50% for *MIR160b* and 67% for *MIR390a*. Our results are in agreement with the results reported by others who also showed that editing efficiency in potato is not consistent when different guide sequences are used, however, their results have not been obtained for miRNA coding regions [2, 10].

The complete pipeline for CRISPR-Cas9-mediated modulation of miRNA expression that we established considers pros and cons of different approaches. As *Agrobacterium*-mediated stable transformation is time consuming, transient transfection of protoplasts following screening by HRM for the selection of optimal sgRNAs in a transient set-up prior stable transformation in combination with ICE software-dependent selection of lines with desired mutations is a significant improvement. We show that by using our pipeline for CRISPR/Cas9-mediated miRNA expression, miRNAs abundance could be fine-tuned in polyploid species with high efficiency in relatively short time. In addition, for the first time, we have characterized in detail the CRISPR-Cas9 transgenic lines that we have generated. We show that all induced mutations do not impair the processing of the modified pre-miRNA, but the processing is less efficient. With the development of new CRISPR-mediated precision breeding tools such as base editing and prime editing [38, 53], we could also envision to precisely quantitatively engineer endogenous miRNA in order to modify their target, conferring a new level for fine-tuning plant physiology.

## Materials and methods

### sgRNA design

Potato mature miRNA and precursor sequences for *MIR160a* and *MIR160b* were obtained from miRBase (Accession No. MI0025955, MI0025956) and for *MIR390a* from the study of Križnik *et al.* [26, 27]. Two sgRNAs were designed to target sense (sgRNA1) and antisense (sgRNA2) strand of each miRNA using Cas-Designer (http://www.rgenome.net/cas-designer/) according to the location (target site close to 5′ end of sense or antisense strain of miRNA), GC content (20% - 80%), out-of-frame score (the highest) and the number of mismatches in the genome (the lowest) (Figure S1). Parameters used were PAM type: SpCas9 from *Streptococcus pyogenes* 5’-NGG-3′, crRNA length (length of target without the NGG PAM motif): 20 nt, target genome: *S. tuberosum* (PGSC v4.03). Designed sgRNAs were ordered as forward and reverse oligonucleotides containing the 20-bp spacer sequence (without PAM) complementary to the target strand with added overhangs compatible with the cloning strategy. To enable cloning into pTwist_ENTR/M1, ATTG or AAAC was added to 5′ end of forward or reverse oligonucleotide, respectively. To enable cloning into pTwist/M2 CTTG or AAAC was added to 5′ end of forward or reverse oligonucleotide, respectively, as described in Chauvin *et al.* [11].

### sgRNA cloning

sgRNAs cloning was performed as explained in [11]. Entry plasmids pTwist_ENTR/M1 and pTwist/M2 (Addgene IDs 173 756–173 758) were digested using *Bsa*I (NEB) according to the provider’s instructions and purified using Wizard® SV Gel and PCR Clean-Up system (Promega; Figure S10). Double-stranded sgRNAs were produced by mixing 1 μl of forward and 1 μl of reverse oligonucleotides (100 μM) with 48 μl of water, incubating 5 min at 95°C in a thermocycler, followed by incubation of the tubes at room temperature for 20 min. sgRNA1 and sgRNA2 were introduced into pTwist_ENTR/M1 and pTwist/M2 entry plasmids, respectively, using T4 DNA ligase (ThermoFischer Scientific; Figure S10). Plasmids were purified from *E. coli* One Shot™ TOP10 cells using NucleoSpin® Plasmid (Macherey-Nagel) and Sanger sequenced using M13F and M13R primers. sgRNA1-containing pTwist_ENTR/M1 was digested using *Mre*I and *BstE*II (ThermoFischer Scientific) and purified using Wizard® SV Gel and PCR Clean-Up system (Promega). sgRNA2 was released from pTwist/M2 using the same restriction enzymes and cloned into *Mre*I- and *BstE*II-digested pTwist_ENTR/M1 using T4 DNA ligase (ThermoFischer Scientific). The presence of the expected construct in the entry plasmid was confirmed by colony PCR and Sanger sequencing. Constructs containing two sgRNAs were introduced into Gateway compatible destination pDeCas9 plant expression vector with kanamycin-*in planta* resistance [16, 20] using Gateway™ LR Clonase™ II Enzyme mix (ThermoFischer Scientific) according to provider’s instructions. The presence of the expected insert in the destination plasmid was confirmed by colony PCR using forward oligonucleotide that served for sgRNA cloning and the SS144 primer (GTCCGGACGTCTTAATTAACC) and Sanger sequencing using SS144 primer. Destination plasmids with inserts and their maps were deposited on Addgene (Addgene ID): pDe_Cas9_KANA_R160a (173756), pDe_Cas9_KANA_R160b (173757), pDe_Cas9_KANA_R390a (173758).

### Plant material

Potato plants for protoplasting (cv. Désirée) were grown in stem node tissue culture in MS10 medium under controlled environmental conditions (20 ± 2°C in the light and 18 ± 2°C in the dark with >90 μmol/m2/s2 radiation (OSRAM L 36 W/77 TL-D 36 W/865) and a 16-h photoperiod). Potato plants (cv. Désirée and cv. Rywal) prior and after stable transformation were grown in stem node tissue culture under controlled environmental conditions (22 ± 2°C in the light and 19 ± 2°C in the dark with 70–90 μmol/m2/s2 radiation (OSRAM L 58 W/77 FLUORA lamps, Germany) and a 16-h photoperiod) as previously explained [33].

### PEG-mediated potato protoplasts transfection

Protoplasts were isolated from leaves of *in vitro* propagated plants of cv. Désirée in stem node tissue culture as explained elsewhere [11]. pDeCas9 destination plasmids containing sgRNA1 and sgRNA2 were purified using the QIAGEN Plasmid Plus Midi Kit (Qiagen), followed by a potassium acetate precipitation (Supplementary MS1). 300 μl of protoplasts with concentration 2^*^10^6^ per μl were transfected with 5 μg of precipitated plasmid DNA. Transfected protoplasts were incubated with 300 μl of 30% PEG (sterile 30% polyethylene glycol 4000 and 4% CaCl_2_ in ddH_2_O, pH 6.7–7.0) for 8 minutes, then the mixture was diluted by gradually applying 10 ml of medium A [11]. Transfected protoplasts were kept in the dark at 25°C. As a control of transfection, we confirmed GFP fluorescence in protoplasts following transfection with a p35S::GFP construct by fluorescent microscopy.

### High-resolution melting analysis (HRM)

Protoplasts from three independent transfections were mixed (6–15 days post transfection), centrifuged for 5 min at 10000 g and the pellet was frozen in liquid nitrogen. Genomic DNA was isolated from the pellet using Nucleo Spin Plant II (Macherey-Nagel) following the protocol. HRM was performed on two technical replicates for each construct (*MIR160a*, *MIR160b* and *MIR390a*) as explained elsewhere [52], but without spiking. See Figure S3 for primer design.

### Genotypisation of transfected protoplasts

We used isolated genomic DNA as a template for HRM to amplify a coding and surrounding region (approximately 800 bp-long) for each miRNA (Figure S11) with Phusion® High-Fidelity DNA Polymerase (NEB). As a control and to determine potential polymorphism in targeted regions, gDNA isolated from non-transfected Désirée was also used as a template. We used Wizard SV Gel and PCR Clean-Up System (Promega) to purify the amplicons from the agarose gel. We cloned PCR products into pJET and transformed them into *E. coli* with CloneJET PCR Cloning Kit (Thermo Fisher Scientific) to determine types of mutations and polymorphism. Next, the constructs were isolated (9 or 10 colonies per PCR product) and Sanger sequenced from both directions as explained elsewhere [34] with primers specific for amplicons (Figure S11). The protocols were performed according to the manufacturer’s instructions. The sequences were aligned using AlignX [32].

### Potato stable transformation

Potato (*S. tuberosum L*.) transgenic lines cr-*MIR160a*, cr-*MIR160b* and cr-*MIR390a* were prepared by stable transformation of Rywal and Désirée genotypes. pDeCas9 destination plasmids containing sgRNA1 and sgRNA2 were electroporated into *Agrobacterium tumefaciens* LBA4404 (Eppendorf Electroporator 2510) following manufacturer’s protocol at 2000 V or chemically transformed into C58pMP90 strain (Supplementary MS2). The transformed bacteria were used for the transformation of stem internodes from in vitro plantlets of cv. Rywal as described elsewhere [37] using spectinomycin for the selection of transformed *Agrobacterium* (final concentration 75 μg/ml) and kanamycin for the selection of transgenic plants (final concentration 50 mg/l). Transformation of cv. Désirée was performed as described in Supplementary MS3. Well-rooted kanamycin-resistant plants were sub-cultured to produce plantlets of the independently transformed lines. Transgenic lines were grown in MS30 medium (Supplementary MS3) under controlled environmental conditions as explained above.

### Identification of transgenic lines by PCR

Genomic DNA was isolated from transgenic lines in tissue cultures using DNeasy Plant Mini Kit (Qiagen). Four different regions of T-DNA (Figure S9a) were amplified from genomic DNA using primers listed in Figure S9b and KAPA2G Robust HotStart PCR Kit (Kapa Biosystems) according to the provider’s instructions.

### Genotypisation of transgenic lines

800 bp-long regions surrounding coding sequence for each miRNA were amplified from genomic DNA and purified as explained above. For the screening of transgenic lines with desired mutations, PCR products were Sanger sequenced (Eurofins Genomics) with primers specific for amplicons (Figure S11). To determine types of mutations and polymorphism, PCR products were for the selected transgenic lines and NT plants (Data S2) cloned into pJET and transformed into *E. coli* as explained above. Next, the constructs were isolated, sequenced and aligned as explained above.

Next, the constructs were isolated from 9 or 10 colonies per PCR product using GenElute™ Plasmid Miniprep Kit (Sigma) and used for Sanger sequencing (Eurofins Genomics) with primers specific for amplicons (Figure S11). All steps were performed according to the manufacturer’s instructions. The sequences were aligned using AlignX [32].

### Off-target effect

Approximately 800 bp-long region surrounding coding sequence for *MIR160a* and *MIR160b* were sequenced as explained in section “Genotypisation of transgenic lines”, but with primers specific for *MIR160a* in cr-*MIR160b* transgenic lines and with primers specific for *MIR160b* in cr-*MIR160a* transgenic lines.

### Inference of CRISPR edits analysis

Inference of CRISPR Edits (ICE) analysis was performed using ICE v2 CRISPR Analysis Tool ( [23]; https://ice.synthego.com) according to the provider’s instructions. The used parameters were the following: GTCGTGTACACGTATATGCC and GAGCTTTGCAAATGGCATAC as guide sequences and DZW775 (Désirée) and CCP522 (Rywal) as control ab1. files for *MIR160a*, AGGAGTAAGAATGATGTGCC and GGTGAAAGTGTGTGGCATAC as guide sequences and DZW857 as a control ab1. file for *MIR160b* and ATGGAGAATCTGTAAAGCTC and AAGATCAATTGAGTCATCCA as guide sequences and EBE422 as a control ab1. file for *MIR390a*. Note that the obtained scores are slightly higher than expected due to a polymorphism as the program predicted a mutation on the position of the natural polymorphism.

### RT-qPCR expression analysis

For detection of miRNA abundance and *Cas9* expression, total RNA was extracted from homogenized leaves from transgenic lines in tissue cultures (several plants per line) using TRizol reagent (Thermo Fisher Scientific) combined with RNA purification on Zymo-Spin columns (Direct-zol RNA MiniPrep Plus Kit, Zymo Research) according to the manufacturer’s protocols. As a control, RNA was extracted from non-transgenic Rywal and Désirée plants. To elute RNA, 50 μl of pre-warmed (80°C) DNase/RNase-free water was added to the Zymo-Spin columns and incubated at room temperature for 10 min before centrifugation at 16000 x g for 1 min. The isolated RNA was subjected to DNase digestion (RNase-Free DNase Set, Qiagen). RNA concentration, quality and purity were determined by agarose gel electrophoresis and NanoDrop ND -1000 spectrophotometer (Thermo Scientific).

Stem-loop RT-qPCR was used to quantify the expression of target miRNAs (miR160a-5p, miR160a-3p, miR160b-5p and miR390a-5p) relative to the endogenous control (miR167a-5p.1; [27]). TaqMan MicroRNA Assays with miRNA-specific stem-loop primers (Thermo Fisher Scientific) were ordered according to the sequence of the selected miRNAs (Assay IDs: 000341, 476214_mat, 001409, 006933_mat). Of note, miR160a-5p and miR160b-5p have identical sequences, therefore the same assay was used for their quantification.

RNA (1 μg) was first reverse transcribed using SuperScript III First-Strand Synthesis System and stem-loop RT Megaplex primer pool (both Thermo Fisher Scientific). To prevent cross-hybridization of the primers of the two complementary miRNAs (miR160a-5p and miR160a-3p), two custom-made RT megaplex primer pools were prepared. The first primer pool contained miR160a-5p, miR390a-5p and miR167a-5p.1 and the second primer pool contained miR160a-3p and miR167a-5p.1. The 20-μl reverse transcription reactions were performed according to the manufacturer’s instructions with the following modifications: use of 100 U SuperScript III enzyme per reaction, 0.5 × stem-loop primer concentration, and use of a pulsed reverse transcription protocol [27]. To determine expression of *Cas9*, total RNA (2 μg) was reverse transcribed using High Capacity cDNA Reverse Transcription kit according to manufacturer’s instructions (Applied Biosystems).

qPCR reactions were performed in 5 μl volumes in duplicates and two dilutions (10- and 50-fold) per sample using 2.75 μl TaqMan Universal Master Mix II and 0.25 μl TaqMan MicroRNA Assays (Applied Biosystems). The qPCR was performed on ViiA 7 or QuantStudio 7 Flex Real-Time PCR system (Applied Biosystems) and using universal cycling conditions (10 min at 95°C; 15 s at 95°C, 1 min at 60°C, 40 cycles). miRNA expression was quantified using a relative standard curve method by normalization to the endogenous control stu-miR167a-5p.1 with quantGenius (http://quantgenius.nib.si; [6]). As a control, we determined baseline miRNA expression in non-transgenic (NT) plants, which we presented as an average of expression in ten plants (see Figure 4 and Data S4). Relative miRNA abundance in transgenic lines was normalised to the averaged expression in NT. For detection of *Cas9, CathB* and *Arf10* expression, qPCR reactions were performed in 5 μl volumes in duplicates and two dilutions (10- and 50-fold) per sample using TaqMan Universal PCR Master Mix (Applied Biosystems) and TaqMan assays (Table S1; Integrated DNA Technologies). The samples were analyzed on 7900HT Fast Real-Time PCR System (Applied Biosystems) using universal cycling conditions as written above. *Cas9* expression was quantified using a relative standard curve method by normalization to the endogenous control cytochrome oxidase (*COX*), while *CathB* and *Arf10* expressions were normalized to the endogenous controls *COX* and elongation factor 1 (*EF-1*) as described previously [35, 46]. As a control, we determined baseline *CathB* and *Arf10* expressions in non-transgenic (NT) plants, which we presented as an average of expression in ten plants (Data S7). Relative *CathB* and *Arf10* abundance in transgenic lines was normalised to the averaged expression in NT.

### Bioinformatics analysis of miRNA precursors

Potato mature miRNA and precursor sequences for *MIR160a* and *MIR160b* were obtained from miRBase (https://www.mirbase.org/; Accession No. MI0025955, MI0025956) and for *MIR390a* from the study of Križnik *et al.* [27]. The secondary structures and minimal folding free energies (MFEs) values of wild-type and mutated pre-miRNAs were predicted using RNAfold (Vienna RNA package v2.4.18) with default settings [31]. MFEI values were calculated as described by [61]. Based on RNAfold results the secondary structures were redrawn and miRNA/miRNA^*^ duplex regions were highlighted using RNA Folding/Annotation tool of The Small RNA Workbench v4.5 [50].

### sRNA-Seq analysis

DNase-treated total RNA from three cr-*MIR160a* lines (i.e. line 3, line 6, line 11) and three cr-*MIR390* lines (i.e. line 2, line 6, line 7) were used for library preparation and sRNA-seq at Novogene UK Ltd. using an Illumina NovaSeq system (50 bp single-end at 20 milion read depth). Along with the six sRNA-Seq samples, in the analysis we also included three sRNA-Seq samples from non-transgenic plants of cv. Désirée from our previous study [27]. The raw sRNA reads were trimmed for 3’adapters and filtered for quality (phred >20). The preprocessed reads were mapped to wild-type pre-miR160a and pre-miRNA390a sequences, or mutated pre-miRNA sequences to identify templated miRNA variants [41, 63]. All mappings were performed using the CLC Genomics Workbench v21 mapping tool (Qiagen), with no mismatches allowed. To identify non-templated miRNA variants being produced from specific *MIR* loci but post-transcriptionally modified at 5′ or 3′ ends, sRNA reads were submitted to isomiRID pipeline [17]. To determine miRNA variants produced specifically from pre-miRNAs of cr-*MIR160a* transgenic lines we excluded those present in sRNA-Seq samples of cv. Désirée or present in cr-*MIR390* lines, since in all those samples the *MIR160a* locus was not affected. Similarly, to determine miRNA variants produced specifically from pre-miRNAs of cr-*MIR390a,* we excluded those present in sRNA-Seq samples of cv. Désirée or present in cr-*MIR160a* lines, as in all those samples the *MIR390*a locus was unaffected. miRNAs and miRNA variants has been counted in CLC Genomics Workbench and normalized by the counts per million (cpm) value. Targets of miRNA variants were predicted using psRNATarget tool v2 with a stricter parameter maximum expectation set to 3 (instead of 5) and other default settings [14].

The sRNA sequencing data have been deposited in the Sequence Read Archive (SRA) at the NCBI database under the accession number PRJNA783147.

## Acknowledgements

We thank Prof. Holger Puchta (Botanical Institute II, Karlsruhe Institute of Technology, Karlsruhe, Germany) for providing the pDeCas9 backbone, Prof. Blanca San Segundo (CRAG, Barcelona) for advice regarding sgRNA design and Mathilde Merrer, Marie-Paule Kermarrec, Lidija Matičič, Nastja Marondini, Brina Dragar, Nina Kobe and Barbara Jaklič for technical assistance. We acknowledge the BrACySol BRC (INRA Ploudaniel, France) that provided us with the cv. Désirée that was used for protoplasting. Potato plants for stable transformation were obtained from the Institute of Plant Breeding and Acclimatisation—National Research Institute, Młochów, Poland (cv. Rywal) and from the Department of Stress and Developmental Biology, Leibniz Institute of Plant Biochemistry, Halle (Saale), Germany (cv. Désirée). This research was financially supported by the Slovenian Research Agency (research core funding no. P4-0165 and projects J4-2544), the Investissement d’Avenir program of the French National Agency of Research for the project GENIUS (ANR-11-BTBR-0001_GENIUS), the Institut Carnot Plant2Pro program for the project POTATOCRISP and COST Action PlantEd (CA18111).

## Author contributions

TL, FV, MK and KG designed the research. TL, FV, LC, TMP, KP, KS performed the research. TL, FV, TMP, KP, KS, MK, KG analyzed the data. TL, FV, ACR, TMP, KP, KS, MK, LC, JEC, KG contributed to the writing or revision of the article.

## Data Availability

Sequences and secondary structures of mutated pre-miRNAs were deposited to Zenodo (https://doi.org/10.5281/zenodo.5727015; [36]). Destination plasmids with inserts and their maps were deposited to Addgene (Addgene ID): pDe_Cas9_KANA_R160a (173756), pDe_Cas9_KANA_R160b (173757), pDe_Cas9_KANA_R390a (173758). The sRNA sequencing data from three cr-MIR160a lines (i.e. line 3, line 6, line 11) and three cr-MIR390 lines (i.e. line 2, line 6, line 7) were deposited to the Sequence Read Archive (SRA) at the NCBI database (accession number PRJNA783147).

## Conflict of interest

The authors declare that they have no conflicts of interest.

## Supplementary data

Supplementary data is available at *Horticulture Research * online.

## Supplementary Material

Web_Material_uhac147
