# Supplementary material for: CRISPR/Cas9-mediated fine-tuning of miRNA expression in tetraploid potato
Source: Hortic Res. 2022 Jun 30;9:uhac147. doi: 10.1093/hr/uhac147 (PMC9437727; doi:10.1093/hr/uhac147)
Supplement: Web_Material_uhac147 [file web_material_uhac147.zip › Figure S1.pdf]

Figure S1: *MIR* loci, *MIR160a* (a), *MIR160b* (b) and *MIR390a* (c) from potato (boxed) and their precursors pre- and pri-miRNAs. Potato mature miRNAs and their precursor sequences for *MIR160a* and *MIR160b* were obtained from miRBase (Accession No. MI0025955, MI0025956) and for *MIR390a* from the study of Križnik *et al.*, 2017 (Kozomara *et al.*, 2019, Križnik *et al.*, 2017). Two sgRNAs (highlighted in grey) were designed to target each *MIR* using Cas-Designer (http://www.rgenome.net/cas-designer/) according to GC content (20% - 80%), out-of-frame score (the highest) and the number of mismatches in the genome (the lowest). One sgRNA (sgRNA1) was targeting sense and the other sgRNA (sgRNA2) was targeting antisense strain of each mature 5p strand miRNA.

a) *MIR160a* locus

miR160a-5p
TGCCTGGCTCCCTGTATGCCA

pre-miR160a
TGCCTGGCTCCCTGTATGCCATTTGCAAAGCTCACCGTAATATATCGATGGGCCTTGTTGAATGGCGTATGAGG
AGCCAAGCATA

Reverse complement
TATGCTTGGCTCCTCATACGCCATTCAACAAGGCCCATCGATATATTACGGTGAGCTTTGCAAATGGCATACAG
GGAGCCAGGCA

pri-miR160a
NNNNNNNNNNNNNNNNNNNNNNNNNNNNNNNNNNNNNNNNNNNNNNNNNNNNNNNNNNNNNNNNNNNNNN
NNNNNNNNNNNNNNNNNNNNNNNNNNNNNNNNNNNNNNNNNNNNNNNNNNNNNNNNNNNNNNNNNNNNNN
NNNNNNNNNNNNNNNNNNNNNNNNNNNNNNNNNNNNNNNNNNNNNNNNNNNNNNNNNNNNNNNNNNNNNN
NNNNNNNNNNNNNNNNNNNNNNNNNNNNNNNNNNNNNNNNNNNNNNNNNNNNNNNNNNNNNNNNNNNNNN
NNNNNNNNNNNNNNNNNNNNNNNNNNNNNNNNNNNNNNNNNNNNNNNNNNNNNNNNNNNNNNNNNNNNNN
NNNNNNNNNNNNNNNNNNNNNNNNNNNNNNNNNNNNNNNNNNNNNNNNNNNNNNNNNNNNNNNNNNNNNN
CAAAAGGAACTTGGCCATTTTCCCCTCTTTATATGTCTCTATATATCTACTAGGTGAAGAACAAGCTTACAAAAG
TTCCTTCTATCTCTCTCAAAGAACATAAAAGAGAAAAAAAACACAAGAGATGAAATATCTCTTCTTATTTCTTGT
GATTTTTAATTTCTTAAGGCTATGCTTGTTTTGTGCCTTTTGAAAAGGGTTATTAATACATTTTATTTTTTTTACTA
GATACACAAAAATGAGAAAGTGAATCTACTTGGTTTTATGGTTGTATACACAATAATAGTTTTTTTTTGTTAAAA
AAAATTACGGGGTTTGAAGATCCATATGATTAGGACCCTAATTTGATCATCTATTTATATTTCTCATTTAATTTTT
ATTTTCACTAGATAAACCATTATTCTCCTCTTCTAAATTCATATTTCTTCATCTTCTTGATTCTTGAAAATATATAT
ATATGATCATGTAGAGATTATGAATTTAAAGAGCTAATAATTATATATATGCATATGAGTATGTGTGTATATGT
ATTGATACACGTCGTGTACACGTATATGCCTGGCTCCCTGTATGCCATTTGCAAAGCTCACCGTAATATATCGA
TGGGCCTTGTTGAATGGCGTATGAGGAGCCAAGCATA

Reverse complement
TATGCTTGGCTCCTCATACGCCATTCAACAAGGCCCATCGATATATTACGGTGAGCTTTGCAAATGGCATACAG
GGAGCCAGGCATATACGTGTACACGACGTGTATCAATACATATACACACATACTCATATGCATATATATAATTA
TTAGCTCTTTAAATTCATAATCTCTACATGATCATATATATATATTTTCAAGAATCAAGAAGATGAAGAAATATG
AATTTAGAAGAGGAGAATAATGGTTTATCTAGTGAAAATAAAAATTAAATGAGAAATATAAATAGATGATCAA
ATTAGGGTCCTAATCATATGGATCTTCAAACCCCGTAATTTTTTTAACAAAAAAAAACTATTATTGTGTATACA
ACCATAAAACCAAGTAGATTCACTTTCTCATTTTTGTGTATCTAGTAAAAAAAATAAAATGTATTAATAACCCTT

TTCAAAAGGCACAAAACAAGCATAGCCTTAAGAAATTAAAAATCACAAGAAATAAGAAGAGATATTTCATCTC
TTGTGTTTTTTTTCTCTTTTATGTTCTTTGAGAGAGATAGAAGGAACTTTTGTAAGCTTGTTCTTCACCTAGTAGA
TATATAGAGACATATAAAGAGGGGAAAATGGCCAAGTTCCTTTTGGTGGGGGTGGGGGTGGGGGGAG

Schematic illustration of the miR160a-5p in the potato genome

G T C G T G T A C A C G T A T A T G C C T G G C T C C C T G T A T G C C A T T T G C A A A G C T C
C A G C A C A T G T G C A T A T A C G G A C C G A G G G A C A T A C G G T A A A C G T T T C G A G

LEGEND
highlighted in grey: sgRNAs
boxed: miRNAs
red: PAM motif

Analysis of sgRNAs by Cas-Designer
(http://www.rgenome.net/cas-designer/)

sgRNA (5' to 3')	Direction	GC contents (20% - 80% recommended)	Out-of-frame score (> 66 recommended)	0	1	2
				mismatches in the genome		
GTCGTGTACACGTATATGCCTGG	+	50	61.1	0	0	0
GAGCTTTGCAAATGGCATACAGG	-	45	71.8	0	0	0

b) *MIR160b* locus

miR160b-5p
TGCCTGGCTCCCTGTATGCCA

pre-miR160b
TGCCTGGCTCCCTGTATGCCACACACTTTCACCAATTCTTTGATTGACTGATCAGTGGGTGGCGTGCGAGGAGC
CAAGCATA

Reverse complement
TATGCTTGGCTCCTCGCACGCCACCCACTGATCAGTCAATCAAAGAATTGGTGAAAGTGTGTGGCATACAGGG
AGCCAGGCA

pri-miR160b
GTATAGATGTTAGTTGAAGCTAAATTAAATGACATATTTATGTGTTATGCCATAGTGCAATTGTGCACAAGTGA
TTCTATATAGTTATATATGCACTATCTGAAATTTAAAATTTAAACATTAATTTGGTATGTTTCTACTCCTACTACT
GCTACTGAATGGATTAATGATCTTATTTATTTGGCATGTCTCTACTGCTACTTAATGGGAAAAAAACAATTTTTT
TCTTTGACAAAGGTGACCAACTTTACAAATGAATCTCACTCAAGAAAAAACAGACCAAAACAAAAAGGGACCT
CCACCCAATTTTGTAAGAAAAAAATGCTAAAAAAATAAATTAAAGAGCATGAAATGAGGAAGAATCATGAAAA
TGAGTCTAAACAATCTATTTTCCCCAACTTTACCTAATCAAAACATTGGTGGAAGCTGATCTTGTTGGTTTGGGA
AAAGTTAAAAAGGTATTTAGGACTTGATAGAAAAAGATGGGTGGAGGGGTCGGTAGTAGTGGTGGGGGTCT

ATTGATATTTTATTGTTTCAAACAACATCAGGTATACAAAATTTATTGATTCGATTGATTTAAATTCATATTGAGT
AATGTCACACTAAGTTTTAAGGGGTAAAGTTGTCTTAGCCTATAAATTCAAAGTTTGATTAATAGTAGAGGTAT
CTCAATCAAATCGATCACTGTACAAATTATTAATTGATTGTGTTTATAAGAGTAATTTTAGCATTAGATTATGGT
ATGATATTTCCTTTATCTTCCATGATTTCAAAAATTTGAACTTTTATGTGTTGTAAGGAAAGTAGAGTAACGGTA
CATTATTATGTACATGGTAATAAACTTTAATATATTATTGACCTTTATATATTCTCATTTATAACTCTTTTCTTAGG
TATATTATAAATATATTCCCCTCATTTCACAAAGAAAGAAGAGGAGAAATTAAAAGAAGAAATTTTGGGCTATT
TGTTTAGCATTGGTGAAGGAGTAAGAATGATGTGCCTGGCTCCCTGTATGCCACACACTTTCACCAATTCTTTG
ATTGACTGATCAGTGGGTGGCGTGCGAGGAGCCAAGCATA

Reverse complement
TATGCTTGGCTCCTCGCACGCCACCCACTGATCAGTCAATCAAAGAATTGGTGAAAGTGTGTGGCATACAGGG
AGCCAGGCACATCATTCTTACTCCTTCACCAATGCTAAACAAATAGCCCAAAATTTCTTCTTTTAATTTCTCCTCT
TCTTTCTTTGTGAAATGAGGGGAATATATTTATAATATACCTAAGAAAAGAGTTATAAATGAGAATATATAAAG
GTCAATAATATATTAAAGTTTATTACCATGTACATAATAATGTACCGTTACTCTACTTTCCTTACAACACATAAAA
GTTCAAATTTTTGAAATCATGGAAGATAAAGGAAATATCATACCATAATCTAATGCTAAAATTACTCTTATAAAC
ACAATCAATTAATAATTTGTACAGTGATCGATTTGATTGAGATACCTCTACTATTAATCAAACTTTGAATTTATA
GGCTAAGACAACTTTACCCCTTAAAACTTAGTGTGACATTACTCAATATGAATTTAAATCAATCGAATCAATAAA
TTTTGTATACCTGATGTTGTTTGAAACAATAAAATATCAATAGACCCCCACCACTACTACCGACCCCTCCACCCA
TCTTTTTCTATCAAGTCCTAAATACCTTTTTAACTTTTCCCAAACCAACAAGATCAGCTTCCACCAATGTTTTGATT
AGGTAAAGTTGGGGAAAATAGATTGTTTAGACTCATTTTCATGATTCTTCCTCATTTCATGCTCTTTAATTTATTT
TTTTAGCATTTTTTTCTTACAAAATTGGGTGGAGGTCCCTTTTTGTTTTGGTCTGTTTTTTCTTGAGTGAGATTCA
TTTGTAAAGTTGGTCACCTTTGTCAAAGAAAAAAATTGTTTTTTTCCCATTAAGTAGCAGTAGAGACATGCCAA
ATAAATAAGATCATTAATCCATTCAGTAGCAGTAGTAGGAGTAGAAACATACCAAATTAATGTTTAAATTTTAA
ATTTCAGATAGTGCATATATAACTATATAGAATCACTTGTGCACAATTGCACTATGGCATAACACATAAATATGT
CATTTAATTTAGCTTCAACTAACATCTATAC

Schematic illustration of the miR160b-5p gene loci in the potato genome

A G G A G T A A G A A T G A T G T G C C T G G C T C C C T G T A T G C C A C A C A C T T T C A C C
T C C T C A T T C T T A C T A C A C G G A C C G A G G G A C A T A C G G T G T G T G A A A G T G G

LEGEND
highlighted in grey: sgRNAs
boxed: miRNAs
red: PAM motif

Analysis of sgRNAs by Cas-Designer
(http://www.rgenome.net/cas-designer/)

sgRNA (5' to 3')	Direction	GC contents (20% - 80% recommended)	Out-of-frame score (> 66 recommended)	0	1	2
				mismatches in the genome		
AGGAGTAAGAATGATGTGCCTGG	+	45	56.8	0	0	0
GGTGAAAGTGTGTGGCATACAGG	-	50	67.0	0	0	0

c) *MIR390a* locus

miR390a-5p

AAGCTCAGGAGGGATAGCGCC

pre-miR390a

GCATGGAGAATCTGTAAAGCTCAGGAGGGATAGCGCCATGGATGATTCAATTGATCTGTTTGCACATCTCTAG
CGCTATCCATCCTGAGTTTTACGGCTTTTTCACGC

Reverse complement

GCGTGAAAAAGCCGTAAAACTCAGGATGGATAGCGCTAGAGATGTGCAAACAGATCAATTGAATCATCCATG
GCGCTATCCCTCCTGAGCTTTACAGATTCTCCATGC

pri-miR390a

CACAATATGTACAGGGTAAGGTCTGCCAGAAATCTTTGTCAAATGAAGGATTTTATGTCTATGTGTGACAGTCC
CATGTGATTGAACGTTGTGACTATTAATACATCATAACATTTGACTCTATCTAAAGGTTACATTTTTTTTTTCCTT
TTCAATTGGTGCGTACAAAGATCAAATTGGTCATCATTAAGATGAAGATTATATTGAAGAAATTCGTAGGCAAT
GATGAAACAAATGTAGAAATTAGGGAGAAAAAAAAAGATTAGGAGAAAAATTGGTACCTACACGGACACAAT
TGTCATAATGAAGAAAATAAAATATTAATGTTAATATTAATACTAATACTAAGTTTGGTATTATTTAACTTTTTAT
TTAATAGTGCTTTCAATCTATTTATATTTATAGTAAATCATGACATTAACAATATCAATACTTCTTTTATTCTAATT
CATTCATATAATACAAAGTTCAGATTTTAACAATTAAATTACCTATTTAATTAATTTGACTATGAGTTAGAGTAT
GAAATCTTTAAATTTTTATGAAAAATTTATAGTAATAAAAAAGGAAATGTCTTTGCATCTCGAAATCTGAAATAC
CAAACATAAATTGAAACAGACAAAATACTTACTACACGTAATAACCGTACGTAGTAGCTAATTTCAAGATTGGT
GAGGTGAAGTAACGTAGTCGGCAGAAGCATTTAAATGAGTAATTCAAGACATAATATATATTATTATAATAAT
AATAATAACCAAAAGAAAGAAAAATAAAAAAAATGAATAGTATTGACCAATAATAGAGACAAATACCACACAA
GTTGAAGAATAATTTTTGTTAGTCAATATACATCCCCACGTTAGTTTGCTACTATAAATAGGTTCTTTTTTTCTTC
AAAACTTTCCCAACCATTCATTTCATCATTTTTGGTGCTGTCCTTTCTTTATGTTTTTCCCTTTCATAAACTATAG
TGAAAAAAATTAATTTTCTGGTGGAAATGCATGGAGAATCTGTAAAGCTCAGGAGGGATAGCGCCATGGATG
ATTCAATTGATCTGTTTGCACATCTCTAGCGCTATCCATCCTGAGTTTTACGGCTTTTTCACGC

Reverse complement

GCGTGAAAAAGCCGTAAAACTCAGGATGGATAGCGCTAGAGATGTGCAAACAGATCAATTGAATCATCCATG
GCGCTATCCCTCCTGAGCTTTACAGATTCTCCATGCATTTCCACCAGAAAATTAATTTTTTTCACTATAGTTTATG
AAAGGGAAAAACATAAAGAAAGGACAGCACCAAAAATGATGAAATGAATGGTTGGGAAAGTTTTGAAGAA
AAAAAGAACCTATTTATAGTAGCAAACTAACGTGGGGATGTATATTGACTAACAAAAATTATTCTTCAACTTGT
GTGGTATTTGTCTCTATTATTGGTCAATACTATTCATTTTTTTTATTTTTCTTTCTTTTGGTTATTATTATTATTATA
ATAATATATATTATGTCTTGAATTACTCATTTAAATGCTTCTGCCGACTACGTTACTTCACCTCACCAATCTTGAA
ATTAGCTACTACGTACGGTTATTACGTGTAGTAAGTATTTTGTCTGTTTCAATTTATGTTTGGTATTTCAGATTTC
GAGATGCAAAGACATTTCCTTTTTTATTACTATAAATTTTTCATAAAAATTTAAAGATTTCATACTCTAACTCATA
GTCAAATTAATTAAATAGGTAATTTAATTGTTAAAATCTGAACTTTGTATTATATGAATGAATTAGAATAAAAGA
AGTATTGATATTGTTAATGTCATGATTTACTATAAATATAAATAGATTGAAAGCACTATTAAATAAAAAGTTAAA
TAATACCAAACTTAGTATTAGTATTAATATTAACATTAATATTTTATTTTCTTCATTATGACAATTGTGTCCGTGT
AGGTACCAATTTTTCTCCTAATCTTTTTTTTTCTCCCTAATTTCTACATTTGTTTCATCATTGCCTACGAATTTCTTC
AATATAATCTTCATCTTAATGATGACCAATTTGATCTTTGTACGCACCAATTGAAAAGGAAAAAAAAAATGTAA
CCTTTAGATAGAGTCAAATGTTATGATGTATTAATAGTCACAACGTTCAATCACATGGGACTGTCACACATAGA
CATAAAATCCTTCATTTGACAAAGATTTCTGGCAGACCTTACCCTGTACATATTGTG

Schematic illustration of the miR390a-5p in the potato genome

A T G G A G A A T C T G T A A A G C T C A G G A G G G A T A G C G C C A T G G A T G A T T C A A T T G A T C T G
T A C C T C T T A G A C A T T T C G A G T C C T C C C T A T C G C G G T A C C T A C T A A G T T A A C T A G A C

LEGEND
highlighted in grey:
sgRNAs
boxed: miRNAs
red: PAM motif

Analysis of sgRNAs by Cas-Designer
(http://www.rgenome.net/cas-designer/)

sgRNA (5' to 3')	Direction	GC contents (20% - 80% recommended)	Out-of-frame score (> 66 recommended)	0	1	2
				mismatches in the genome		
ATGGAGAATCTGTAAAGCTCAGG	+	40	78.6	0	0	1
CAGATCAATTGAATCATCCATGG	-	35	65.5	0	0	0
