# Supplementary material for: CRISPR/Cas9-mediated fine-tuning of miRNA expression in tetraploid potato
Source: Hortic Res. 2022 Jun 30;9:uhac147. doi: 10.1093/hr/uhac147 (PMC9437727; doi:10.1093/hr/uhac147)

Figure S10: CRISPR-Cas9 cloning strategy for introducing two guide sequences (sgRNA) in potato. Each entry plasmid harbours a RNA PolIII promoter (U6) from *Arabidopsis* (At) or *Solanum tuberosum* (St) and an enhanced single guide RNA scaffold (esgRNA; not shown). sgRNAs cloning was performed as explained in Chauvin *et al.,* 2021. Briefly, guide sequences (sgRNA1, sgRNA2) are cloned into an entry plasmid (harbouring *Bsa*I restriction sites) using T4 DNA ligase. For multiplexing, the CRISPR functional module of pTwist/M2 is flanked by unique restriction sites, allowing cloning into the pTwist_ENTR/M1 using T4 DNA ligase. The guide cassette, harbouring one or two modules, is transferred into a destination plasmid, which includes the *ccdB* counter selection, through a Gateway LR reaction. The final construct can be used for *Agrobacterium*-mediated transformation and PEG-mediated protoplast transfection. LB: left border of T-DNA, RB: right border of T-DNA, PcUBI: *Petroselinum crispum* Ubiquitin4-2 promoter, Kan: kanamycin resistance gene, Cm: chloramphenicol resistance gene, Spec: spectinomycin resistance gene.

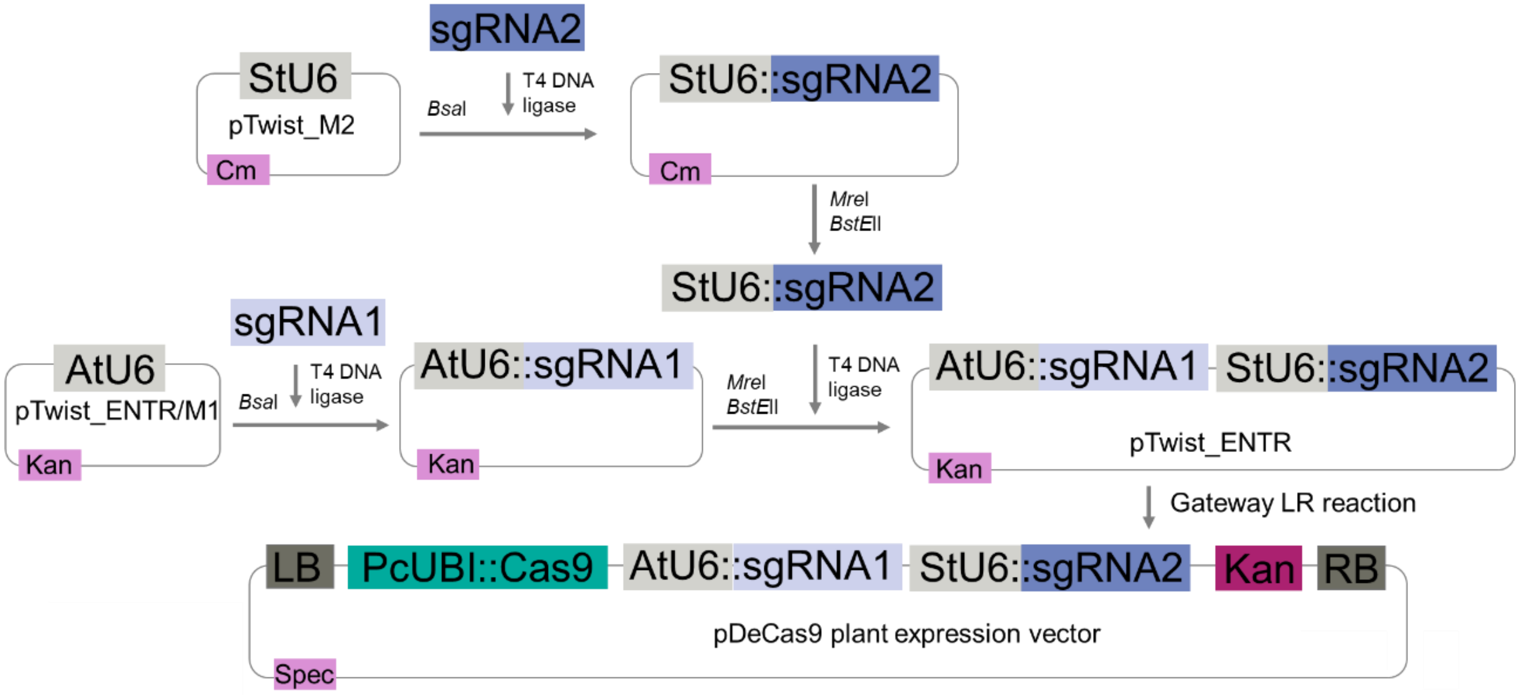

Supplement: Web_Material_uhac147 [file web_material_uhac147.zip › Figure S10.pdf]
