# Supplementary material for: CRISPR/Cas9-mediated fine-tuning of miRNA expression in tetraploid potato
Source: Hortic Res. 2022 Jun 30;9:uhac147. doi: 10.1093/hr/uhac147 (PMC9437727; doi:10.1093/hr/uhac147)
Supplement: Web_Material_uhac147 [file web_material_uhac147.zip › Figure S2.pdf]

Figure S2: MIR loci in the genome of potato. Sequences of miRNAs coding regions (miR-5p and miR-3p; boxed) and their precursors (pre-miRNAs; not italics) are shown in the genome of potato (italics). Guide sequences (Figure S1) are highlighted in grey.

MIR160a locus

miR160a-5p miR160a-3p

*ATTGATACACGTCGTGTACACGTATA*TGCCTGGCTCCCTGTATGCCATTTGCAAAGCTCACCGTAATATATCGATGGGCCTTGTTGAATGGCGTATGAGGAGCCAAGCATA
*TAACTATGTGCAGCACATGTGCATAT*ACGGACCGAGGGACATACGGTAAACGTTTCGAGTGGCATTATATAGCTACCCGGAACAACTTACCGCATACTCCTCGGTTCGTAT

MIR160b locus

miR160b-5p miR160b-3p

*GCATTGGTGAAGGAGTAAGAATGATG*TGCCTGGCTCCCTGTATGCCACACACTTTCACCAATTCTTTGATTGACTGATCAGTGGGTGGCGTGCGAGGAGCCAAGCATA
*CGTAACCACTTCCTCATTCTTACTAC*ACGGACCGAGGGACATACGGTGTGTGAAAGTGGTTAAGAAACTAACTGACTAGTCACCCACCGCACGCTCCTCGGTTCGTAT

MIR390a locus

miR390a-5p miR390a-3p

*TGGTGGAAAT*GCATGGAGAATCTGTAAAGCTCAGGAGGGATAGCGCCATGGATGATTCAATTGATCTGTTTGCACATCTCTAGCGCTATCCATCCTGAGTTTTACGGCTTTTTCACGC
*ACCACCTTTA*CGTACCTCTTAGACATTTCGAGTCCTCCCTATCGCGGTACCTACTAAGTTAACTAGACAAACGTGTAGAGATCGCGATAGGTAGGACTCAAAATGCCGAAAAAGTGCG

Not italics...precursor pre-miRNA
Highlighted in grey...sgRNAs
Boxed...miRNAs
