# Supplementary material for: CRISPR/Cas9-mediated fine-tuning of miRNA expression in tetraploid potato
Source: Hortic Res. 2022 Jun 30;9:uhac147. doi: 10.1093/hr/uhac147 (PMC9437727; doi:10.1093/hr/uhac147)
Supplement: Web_Material_uhac147 [file web_material_uhac147.zip › Figure S3.pdf]

Figure S3: miRNAs miR160a-5p (a), miR160b-5p (b) and miR390a-5p (c) from potato (boxed) were located in the genome of cv. Désirée (Sevestre *et al.*, 2020). Primers were designed to amplify 80-200 bp-long region surrounding each miRNA. sgRNAs targeting miRNAs are highlighted in grey. Bold italics - regions where primers anneal.

a) miR160a-5p

G T C G T G T A C A C G T A T A T G C C T G G C T C C C T G T A T G C C A T T T G C A A A G C T C
C A G C A C A T G T G C A T A T A C G G A C C G A G G G A C A T A C G G T A A A C G T T T C G A G

LEGEND
grey: sgRNAs
boxed: miRNAs
red: PAM motif

Sequence from potato cv. Désirée (chr05, antisense strain)

TTGTGTAATAATTGTGTAAATATTTATATATTATAAGTTCATACAACTATAAACTCATTGCAAAAAGATAAAAGG
TTTTTTTTTATTTTCCTTTGTTTGCAAAAGATCCTAAAAACTACAATAAAGAGTAAGAATACATTTTATAAAAAGT
ATAATTCTCTAGGTGACAGAAACAAAAACAGGTAGCATAGATTTGTGGTTATTTAAACTTGAAGTTGTGTTTTG
ACATGCATTTTACTTAGTAAGAAATGAATTATTATGAAAGAAAGTGTAAATTTATTCGGAAGAAAATTTTCAAA
ATCTGGTAAAATTTAATGTATAAACACATATATAAAGATAAATTATAAAAATCTGATTCAAAATCTATGGTGAA
ATATGCTTAACTTAAATACTAGCTTAGCACTAACATTAATCCATCTACTAATATCAAAGTTTCTTGTAGAATAATA
ACCACAAGAAAATCCAAACATTAAAATTTAATCAAAGTAAAATCAAACATTCACTAAACATTACATATAGCAAA
TTTTAAAAAAGTTACAAACTTATTTTTTTAAAATATAATTAAAAGCACATATATAGGAAGCAAATTAAGAAGCCC
TAGACATATAATCAATTATCATGATGTAAATTTAGAAAGTAGACAGGATGAATTAATTTTAAAAAATACTTACC
TGTTTCAACTGAGAATGAAGAATTCTTCAACAATCACCATAACCTAGTTTAGATCTCAACATAAACAAATTAAAT
TAATAGAAAGAAAGAAAAAAAGAGGTAACTTTATATCACAAGATGGTTACATATATCAACATCATATACACGA
TATCGGAAT***ATGCTTGGCTCCTCATACGC***CATTCAACAAGGCCCATCGATATATTACGGTGAGCTTTGCAAATG
GCATACAGGGAGCCAGGCATATACGTGTA***CACGACGTGTATCAATACATATACACA***CATACTCATATGCATAT
ATATAATTATTAGCTCTTTAAATTCATAATCTCTACATGATCATATATATATATTTTCAAGAATCAAGAAGATGAA
GAAATATGAATTTAGAAGAGGAGAATAATGGTTTATCTAGTGAAAATAAAAATTAAATGAGAAATATAAATAG
ATGATCAAATTAGGGTCCTAATCATATGGATCTTCAAACCCCGTAATTTTTTTTAACAAAAAAAAACTATTATTG
TGTATACAACCATAAAACCAAGTAGATTCACTTTCTCATTTTTGTGTATCTAGTAAAAAAAATAAAATGTATTAA
TAACCCTTTTCAAAAGGCACAAAACAAGCATAGCCTTAAGAAATTAAAAATCACAAGAAATAAGAAGAGATAT
TTCATCTCTTGTGTTTTTTTTCTCTTTTATGTTCTTTGAGAGAGATAGAAGGAACTTTTGTAAGCTTGTTCTTCACC
TAGTAGATATATAGAGACATATAAAGAGGGGAAAATGGCCAAGTTCCTTTTGGTGGGGGTGGGGGTGGGGG
GAG

PRIMERS

miR160a_HRM_F
TGTGTATATGTATTGATACACGTCGTG

miR160a_HRM_R_RC
ATGCTTGGCTCCTCATACGC

b) miR160b-5p

A G G A G T A A G A A T G A T G T G C C T G G C T C C C T G T A T G C C A C A C A C T T T C A C C
T C C T C A T T C T T A C T A C A C G G A C C G A G G G A C A T A C G G T G T G T G A A A G T G G

LEGEND
grey: sgRNAs
boxed: miRNAs
red: PAM motif

Sequence from potato cv. Désirée (chr02, sense strain)

AATGAGTCTAAACAATCTATTTTCCCCAACTTTACCTAATCAAAACATTGGTGGAAGCTGATCTTGTTGGTTTGG
GAAAAGTTAAAAAGGTATTTAGGACTTGATAGAAAAAGATGGGTGGAGGGGTCGGTAGTAGTGGTGGGGGT
CTATTGATATTTTATTGTTTCAAACAACATCAGGTATACAAAATTTATTGATTCGATTGATTTAAATTCATATTGA
GTAATGTCACACTAAGTTTTAAGGGGTAAAGTTGTCTTAGCCTATAAATTCAAAGTTTGATTAATAGTAGAGGT
ATCTCAATCAAATCGATCACTGTACAAATTATTAATTGATTGTGTTTATAAGAGTAATTTTAGCATTAGATTATG
GTATGATATTTCCTTTATCTTCCATGATTTCAAAAATTTGAACTTTTATGTGTTGTAAGGAAAGTAGAGTAACGG
TACATTATTATGTACATGGTAATAAACTTTAATATATTATTGACCTTTATATATTCTCATTTATAACTCTTTTCTTA
GGTATATTATAAATATATTCCCCTCATTTCACAAAGAAAGAAGAGGAGAAATTAAAAGAAGAAATTTTG***GGCT***
ATTTGTTTAGCATTGGTGAAGGAGTAAGAATGATGTGCCTGGCTCCCTGTATGCCACACACTTTCACCAATTCT
TTGATTGACT***GATCAGTGGGTGGCGTGC***GAGGAGCCAAGCATACCACCCGCTTTCTCATTTTCAATTTTTTTCT
GAGATTGCTATATATACTTGTTGATTTGAAAAACAAAAGTCGCTCTAAATTTTCCTCATTTTTTAATTTGTGTGTG
TGTTATGTGATTATGTTGCGTTGATTTCATCTGTCATTTCTACTTCTAATTTTAAACTAAGGTAATTGTCATTTAA
TGGTGTGAGCGGAATTTTGCAGAAGTGGTGTCACCCTACTGTTGCAATTCATCTGGGCGGTAAGATCAAGGAG
TATGCTTCCCGATACCTCCCAAGGGAAATCTCTAATTTGAATTTCAAATTGACGGATTAATGTGAGCTTATCCAT
GTGGTTATGCACTCTTTGAATAGGAATCTGTTTGCTGAAAGATCCTATAAAGAATATGATGATTATATTGATCTT
CTGAATGCAAATTATATACTAGTATTAATTTTCCTTCTGTTGAATGGGCTTCTTTGTATACAAGTTATAACTTCTT
GTGTCTTATTATTATATTAATCGTATAATCCAATAGTTTCAAGAGATTTTAAGTATTGTCACTTTGCCAATGATG
GACTTAGTGTTGCATAGTAAAAGAAACTAAGGACATTTTCAATATCAATTACGATTTTTGAAGAGATA

PRIMERS

miR160b_HRM_F
GGCTATTTGTTTAGCATTGGTGA

miR160b_HRM_R_RC
GCACGCCACCCACTGATC

c) miR390a-5p

A T G G A G A A T C T G T A A A G C T C A G G A G G G A T A G C G C C A T G G A T G A T T C A A T T G A T C T G
T A C C T C T T A G A C A T T T C G A G T C C T C C C T A T C G C G G T A C C T A C T A A G T T A A C T A G A C

LEGEND
grey: sgRNAs
boxed: miRNAs
red: PAM motif

Sequence from potato cv. Désirée (chr09, sense strain)

TTTCAATCTATTTATATTTATAGTAAATCATGACATTAACAATATCAATACTTCTTTTATTCTAATTCATTCATATA
ATACAAAGTTCAGATTTTAACAATTAAATTACCTATTTAATTAATTTGACTATGAGTTAGAGTATGAAATCTTTA
AATTTTTATGAAAAATTTATAGTAATAAAAAAGGAAATGTCTTTGCATCTCGAAATCTGAAATACCAAACATAA
ATTGAAACAGACAAAATACTTACTACACGTAATAACCGTACGTAGTAGCTAATTTCAAGATTGGTGAGGTGAA
GTAACGTAGTCGGCAGAAGCATTTAAATGAGTAATTCAAGACATAATATATATTATTATAATAATAATAATAAC
CAAAAGAAAGAAAAATAAAAAAAAATGAATAGTATTGACCAATAATAGAGACAAATACCACACAAGTTGAAGA
ATAATTTTTGTTAGTCAATATACATCCCCACGTTAGTTTGCTACTATAAATAGGTTCTTTTTTTCTTCAAAACTTTC
CCAACCATTCATTTCATCATTTTTGGTGCTGTCCTTTCTTTATGTTTTTTCCCTTTCATAAACTATAGTGAAAAAAA
TTAATT***TTCTGGTGGAAATGCATGGA***GAATCTGTAAAGCTCAGGAGGGATAGCGCCATGGATGATTCAATTG
ATCTGTTTGCACAT***CTCTAGCGCTATCCATCCTGAGT***TTTACGGCTTTTTCACGCTCATTTTTTAATGCATCACTTT
TTTTTTATCTGCTCTAGGGTCTAATAAAATTGGATTTGTGCAAGGAAGATTCCATTGTGAAGGTAAAATGCGTT
CATTATTGAAGAGAATTTTGATTTTCAAAATTTGAATTTGAAATGTCTTACTAATCGAACTAACATAATAGTTAA
TTGTAATGCATGCTGTTGAATATCCGATACTACACTAGTACTCCTATGTGGTTCAGCTTAAATTTCAGTTAATTTC
TGAATAAAATGGTTCTTTTGGTTGTCTTTAATTTTGATATGCTAAATAATTATTCAATTTAAAACACATATAATTA
ACTCGTACACTGTCAGTTTTTTTAAAAAAAATTAACATTTGTTTTTTACTTTACTGTGATATTTTAATATATTATTT
TTTCGCTGTCACTATTAATCTGATATTTTTTTTTTGTTTATTGAGAGTATGTTTGACTAATCTTGAAAGTTAGACT
GAATAAGATCAATTAATTATTTTAAAATAATTAAAATTTATACAAAATATTATAAGATATAATTATATATTTTCAT
GGTAATATGATGAAAA

PRIMERS

miR390a_HRM_F
TTCTGGTGGAAATGCATGGA

miR390a_HRM_R_RC
ACTCAGGATGGATAGCGCTAGAG

Sevestre F, Facon M, Wattebled F, Szydlowski N. 2020. Facilitating gene editing in potato: a Single-Nucleotide Polymorphism (SNP) map of the *Solanum tuberosum* L. cv. Desiree genome. *Sci Rep* **10**: 2045.
