# Supplementary material for: CRISPR/Cas9-mediated fine-tuning of miRNA expression in tetraploid potato
Source: Hortic Res. 2022 Jun 30;9:uhac147. doi: 10.1093/hr/uhac147 (PMC9437727; doi:10.1093/hr/uhac147)
Supplement: Web_Material_uhac147 [file web_material_uhac147.zip › Figure S4.pdf]

Figure S4: Allelic variation in miRNAs coding regions and adjacent regions in protoplasts of non-transgenic potato cv. Désirée. Results of Sanger sequencing for individual PCR products covering coding regions and adjacent regions of three *MIR* loci: a) *MIR160a*, b) *MIR160b* and c) *MIR390a*. 9 or 10 individual PCR products are shown. Results are presented as consensus sequence, obtained by forward and reverse primers (Figure S11). See Polymorphism in NT protoplasts sheet in Data S1 for sequencing IDs, primers used and results. See (https://doi.org/10.5281/zenodo.5727015) for individual sequencing results. miRNA (boxed), sgRNA1 (light grey), sgRNA2 (dark grey), PAM motif (red), theoretical cutting site (arrowhead) in the *MIR* loci are shown above the alignments. Allelic variation is shown by blue.

a) *MIR160a* locus

```
                                            ▼              ▼
                              GTCGTGTACACGTATATGCCTGGCTCCCTGTATGCCATTTGCAAAGCTC

miR160a_NT-proto_col.2        GTCGTGTACACGTATATGCCTGGCTCCCTGTATGCCATTTGCAAAGCTC
miR160a_NT-proto_col.3        GTCGTGTACACGTATATGCCTGGCTCCCTGTATGCCATTTGCAAAGCTC
miR160a_NT-proto_col.4        GTCGTGTACACGTATATGCCTGGCTCCCTGTATGCCATTTGCAAAGCTC
miR160a_NT-proto_col.5        GTCGTGTACACGTATATGCCTGGCTCCCTGTATGCCATTTGCAAAGCTC
miR160a_NT-proto_col.6        GTCGTGTACACGTATATGCCTGGCTCCCTGTATGCCATTTGCAAAGCTC
miR160a_NT-proto_col.7        GTCGTGTACACGTATATGCCTGGCTCCCTGTATGCCATTTGCAAAGCTC
miR160a_NT-proto_col.8        GTCGTGTACACGTATATGCCTGGCTCCCTGTATGCCATTTGCAAAGCTC
miR160a_NT-proto_col.9        GTCGTGTACACGTATATGCCTGGCTCCCTGTATGCCATTTGCAAAGCTC
miR160a_NT-proto_col.10       GTCGTGTACACGTATATGCCTGGCTCCCTGTATGCCATTTGCAAAGCTC
```

b) *MIR160b* locus

```
                                            ▼              ▼
                              AGGAGTAAGAATGATGTGCCTGGCTCCCTGTATGCCACACACTTTCACC

miR160b_NT-proto_col.1        AGGAGTAAGAATGATGTGCCTGGCTCCCTGTATGCCACACACTTTCACC
miR160b_NT-proto_col.2        AGGAGTAAGAATGATGTGCCTGGCTCCCTGTATGCCACACACTTTCACC
miR160b_NT-proto_col.3        AGGAGTAAGAATGATGTGCCTGGCTCCCTGTATGCCACACACTTTCACC
miR160b_NT-proto_col.4        AGGAGTAAGAATGATGTGCCTGGCTCCCTGTATGCCACACACTTTCACC
miR160b_NT-proto_col.5        AGGAGTAAGAATGATGTGCCTGGCTCCCTGTATGCCACACACTTTCACC
miR160b_NT-proto_col.6        AGGAGTAAGAATGATGTGCCTGGCTCCCTGTATGCCACACACTTTCACC
miR160b_NT-proto_col.7        AGGAGTAAGAATGGTGTGCCTGGCTCCCTGTATGCCACACACTTTCACC
miR160b_NT-proto_col.8        AGGAGTAAGAATGGTGTGCCTGGCTCCCTGTATGCCACACACTTTCACC
miR160b_NT-proto_col.9        AGGAGTAAGAATGATGTGCCTGGCTCCCTGTATGCCACACACTTTCACC
miR160b_NT-proto_col.10       AGGAGTAAGAATGATGTGCCTGGCTCCCTGTATGCCACACACTTTCACC
```

c) *MIR390a* locus

```
                                                   ▼                   ▼
                              ATGGAGAATCTGTAAAGCTCAGGAGGGATAGCGCCATGGATGACTCAATT-GATCTG

miR390a_NT-proto_col.1        ATGGAGAATCTGTAAAGCTCAGGAGGGATAGCGCCATGGATGACTCAATTTGATCTT
miR390a_NT-proto_col.2        ATGGAGAATCTGTAAAGCTCAGGAGGGATAGCGCCATGGATGACTCAATTTGATCTT
miR390a_NT-proto_col.3        ATGGAGAATCTGTAAAGCTCAGGAGGGATAGCGCCATGGATGACTCAATT-GATCTT
miR390a_NT-proto_col.4        ATGGAGAATCTGTAAAGCTCAGGAGGGATAGCGCCATGGATGACTCAATT-GATCTT
miR390a_NT-proto_col.5        ATGGAGAATCTGTAAAGCTCAGGAGGGATAGCGCCATGGATGACTCAATT-GATCTT
miR390a_NT-proto_col.6        ATGGAGAATCTGTAAAGCTCAGGAGGGATAGCGCCATGGATGACTCAATT-GATCTT
miR390a_NT-proto_col.7        ATGGAGAATCTGTAAAGCTCAGGAGGGATAGCGCCATGGATGACTCAATTTGATCTT
miR390a_NT-proto_col.8        ATGGAGAATCTGTAAAGCTCAGGAGGGATAGCGCCATGGATGACTCAATTTGATCTT
miR390a_NT-proto_col.9        ATGGAGAATCTGTAAAGCTCAGGAGGGATAGCGCCATGGATGACTCAATT-GATCTT
miR390a_NT-proto_col.10       ATGGAGAATCTGTAAAGCTCAGGAGGGATAGCGCCATGGATGACTCAATT-GATCTT
```
