# Supplementary material for: CRISPR/Cas9-mediated fine-tuning of miRNA expression in tetraploid potato
Source: Hortic Res. 2022 Jun 30;9:uhac147. doi: 10.1093/hr/uhac147 (PMC9437727; doi:10.1093/hr/uhac147)
Supplement: Web_Material_uhac147 [file web_material_uhac147.zip › Figure S6.pdf]

Figure S6: CRISPR-mediated mutations in miRNAs coding and adjacent regions in transgenic potato plants. Results of Sanger sequencing for individual PCR products (col.) covering coding regions and adjacent regions of *MIR* loci: a) *MIR160a* in Désirée and Rywal, b) *MIR160b* in Désirée and c) *MIR390a* in Désirée (bottom; line) are shown to identify types and frequency of mutations. Allelic variations in non-transgenic potato cv. Désirée and cv. Rywal (top; NT) are also shown. Results are presented as consensus sequence, obtained by forward and reverse primers (Figure S11). See Type of mutations sheet in Data S2 and Data S3 for for sequencing IDs, primers used and types of mutations. See https://doi.org/10.5281/zenodo.5727015 for individual sequencing results. miRNA (boxed), sgRNA1 (light grey), sgRNA2 (dark grey), PAM motif (red), theoretical cutting site (arrowhead) in the *MIR* loci are shown above the alignment. Allelic variations are shown by blue, mutations are shown by orange.

a) *MIR160a* locus

Désirée

```
                                   GTCGTGTACACGTATAT-GCCTGGCTCCCTGTAT-GCCATTTGCAAAGCTC

miR160a_NT_col.1                   GTCGTGTACACGTATAT-GCCTGGCTCCCTGTAT-GCCATTTGCAAAGCTC
miR160a_NT_col.2                   GTCGTGTACACGTATAT-GCCTGGCTCCCTGTAT-GCCATTTGCAAAGCTC
miR160a_NT_col.3                   GTCGTGTACACGTATAT-GCCTGGCTCCCTGTAT-GCCATTTGCAAAGCTC
miR160a_NT_col.4                   GTCGTGTACACGTATAT-GCCTGGCTCCCTGTAT-GCCATTTGCAAAGCTC
miR160a_NT_col.6                   GTCGTGTACACGTATAT-GCCTGGCTCCCTGTAT-GCCATTTGCAAAGCTC
miR160a_NT_col.7                   GTCGTGTACACGTATAT-GCCTGGCTCCCTGTAT-GCCATTTGCAAAGCTC
miR160a_NT_col.8                   GTCGTGTACACGTATAT-GCCTGGCTCCCTGTAT-GCCATTTGCAAAGCTC
miR160a_NT_col.9                   GTCGTGTACACGTATAT-GCCTGGCTCCCTGTAT-GCCATTTGCAAAGCTC
miR160a_NT_col.10                  GTCGTGTACACGTATAT-GCCTGGCTCCCTGTAT-GCCATTTGCAAAGCTC
miR160a_NT_col.11                  GTCGTGTACACGTATAT-GCCTGGCTCCCTGTAT-GCCATTTGCAAAGCTC

miR160a_NT-proto_col.2             GTCGTGTACACGTATAT-GCCTGGCTCCCTGTAT-GCCATTTGCAAAGCTC
miR160a_NT-proto_col.3             GTCGTGTACACGTATAT-GCCTGGCTCCCTGTAT-GCCATTTGCAAAGCTC
miR160a_NT-proto_col.4             GTCGTGTACACGTATAT-GCCTGGCTCCCTGTAT-GCCATTTGCAAAGCTC
miR160a_NT-proto_col.5             GTCGTGTACACGTATAT-GCCTGGCTCCCTGTAT-GCCATTTGCAAAGCTC
miR160a_NT-proto_col.6             GTCGTGTACACGTATAT-GCCTGGCTCCCTGTAT-GCCATTTGCAAAGCTC
miR160a_NT-proto_col.7             GTCGTGTACACGTATAT-GCCTGGCTCCCTGTAT-GCCATTTGCAAAGCTC
miR160a_NT-proto_col.8             GTCGTGTACACGTATAT-GCCTGGCTCCCTGTAT-GCCATTTGCAAAGCTC
miR160a_NT-proto_col.9             GTCGTGTACACGTATAT-GCCTGGCTCCCTGTAT-GCCATTTGCAAAGCTC
miR160a_NT-proto_col.10            GTCGTGTACACGTATAT-GCCTGGCTCCCTGTAT-GCCATTTGCAAAGCTC

miR160a_line3_col.1_A.tumLBA       GTCGTGTACACGTATAT-GCCTGGCTCCCTGTAT-GCCATTTGCAAAGCTC
miR160a_line3_col.2_A.tumLBA       GTCGTGTACACGTATATTGCCTGGCTCCCTGTA------TTTGCAAAGCTC
miR160a_line3_col.3_A.tumLBA       GTCGTGTACACGTATAT-GCCTGGCTCCCTGTAT-GCCATTTGCAAAGCTC
miR160a_line3_col.4_A.tumLBA       GTCGTGTACACGT-----GCCTGGCTCCCTGTAT----ATTTGCAAAGCTC
miR160a_line3_col.5_A.tumLBA       GTCGTGTACACGTATATTGCCTGGCTCCCTGTAT-GCCATTTGCAAAGCTC
miR160a_line3_col.6_A.tumLBA       GTCGTGTACACGTATATTGCCTGGCTCCCTGTAT-GCCATTTGCAAAGCTC
miR160a_line3_col.7_A.tumLBA       GTCGTGTACACGTATATTGCCTGGCTCCCTGTAT-GCCATTTGCAAAGCTC
miR160a_line3_col.8_A.tumLBA       GTCGTGTACACGTATATTGCCTGGCTCCCTGTAT-GCCATTTGCAAAGCTC
miR160a_line3_col.9_A.tumLBA       GTCGTGTACACGTATAT----------------T-GCAAATGGCAAAGCTC
miR160a_line3_col.10_A.tumLBA      GTCGTGTACACGTATAT-GCCTGGCTCCCTGTAT-GCCATTTGCAAAGCTC

miR160a_line6_col.1_A.tumLBA       GTCGTGTACAC-----T-GCCTGGCTCCCTGTA------------AAGCTC
miR160a_line6_col.2_A.tumLBA       GTCGTGTACAC-------GCCTGGCTCCCTGTAT----ATTTGCAAAGCTC
miR160a_line6_col.3_A.tumLBA       GTCGTGTACACGTAT-T-GCCTGGCTCCCTGTATTGCCATTTGCAAAGCTC
miR160a_line6_col.4_A.tumLBA       GTCGTGTACACGTAT-T-GCCTGGCTCCCTGTA-----ATTTGCAAAGCTC
miR160a_line6_col.5_A.tumLBA       GTCGTGTACAC-------GCCTGGCTCCCTGTA---------GCAAAGCTC
miR160a_line6_col.6_A.tumLBA       GTCGTGTAC---------GCCTGGCTCCCTGTA-----ATTTGCAAAGCTC
miR160a_line6_col.7_A.tumLBA       GTCGTGTACACGTAT-T-GCCTGGCTCCCTGTAT-GCCATTTGCAAAGCTC
miR160a_line6_col.8_A.tumLBA       GTCGTGTA------------------CCCTGTAT-GCCATTTGCAAAGCTC
miR160a_line6_col.9_A.tumLBA       GTCGTGTACACGTAT-T-GCCTGGCTCCCTG-----CCATTTGCAAAGCTC
miR160a_line6_col.10_A.tumLBA      GTCGTGTACACGTATATTGCCTGGCTCCCTGTA-----ATTTGCAAAGCTC

miR160a_line8_col.2_A.tumLBA       GTCGTGTACACGTATAT-GCCTGGCTCCCTGTAT-GCCATTTGCAAAGCTC
miR160a_line8_col.3_A.tumLBA       GTCGTGTACACGTATATTGCCTGGCTCCCTGTAT-GCCATTTGCAAAGCTC
miR160a_line8_col.4_A.tumLBA       GTCGTGTACACGTAT---GCCTGGCTCCCTGTAT------TTGCAAAGCTC
miR160a_line8_col.5_A.tumLBA       GTCGTGTACACGTATATTGCCTGGCTCCCTGTAT------TTGCAAAGCTC
miR160a_line8_col.6_A.tumLBA       GTCGTGTACACGTAT---GCCTGGCTCCCTGTAT------TTGCAAAGCTC
miR160a_line8_col.7_A.tumLBA       GTCGTGTACACGTATATTGCCTGGCTCCCTGTAT-GCCATTTGCAAAGCTC
miR160a_line8_col.8_A.tumLBA       GTCGTGTACACGTATATTGCCTGGCTCCCTGTAGTGCCATTTGCAAAGCTC
miR160a_line8_col.9_A.tumLBA       GTCGTGTACACGTATAT-GCCTGGCTCCCTGTAT-GCCATTTGCAAAGCTC
miR160a_line8_col.10_A.tumLBA      GTCGTGTACAC-----T-GCCTGGCTCCCTGTAT-GCCATTTGCAAAGCTC
miR160a_line8_col.11_A.tumLBA      GTCGTGTACACGTATAT-GCCTGGCTCCCTGTAT-GCCATTTGCAAAGCTC

miR160a_line9_col.1_A.tumLBA       GTCGTGTACACGTATAT-GCCTGGCTCCCTGTAT-GCCATTTGCAAAGCTC
miR160a_line9_col.2_A.tumLBA       GTCGTGTACACGTATAT-GCCTGGCTCCCTGTAT-GCCATTTGCAAAGCTC
miR160a_line9_col.3_A.tumLBA       GTCGTGTACACGTATAT-GCCTGGCTCCCTGTAT-GCCATTTGCAAAGCTC
miR160a_line9_col.4_A.tumLBA       GTCGTGTACACGTATAT-GCCTGGCTCCCTGTAT-GCCATTTGCAAAGCTC
miR160a_line9_col.5_A.tumLBA       GTCGTGTACACGTATAT-GCCTGGCTCCCTGTAT-GCCATTTGCAAAGCTC
miR160a_line9_col.6_A.tumLBA       GTCGTGTACACGTATAT-GCCTGGCTCCCTGTAT-GCCATTTGCAAAGCTC
miR160a_line9_col.7_A.tumLBA       GTCGTGTACACGTATAT-GCCTGGCTCCCTGTAT-GCCATTTGCAAAGCTC
miR160a_line9_col.8_A.tumLBA       GTCGTGTACACGTATAT-GCCTGGCTCCCTGTAT-GCCATTTGCAAAGCTC
miR160a_line9_col.9_A.tumLBA       GTCGTGTACACGTATAT-GCCTGGCTCCCTGTAT-GCCATTTGCAAAGCTC
miR160a_line9_col.10_A.tumLBA      GTCGTGTACACGTATAT-GCCTGGCTCCCTGTAT-GCCATTTGCAAAGCTC
miR160a_line9_col.11_A.tumLBA      GTCGTGTACACGTATAT-GCCTGGCTCCCTGTAT-GCCATTTGCAAAGCTC
```

```
miR160a_line11_col.1_A.tumLBA      GTCGTGTACACGTATAT-GCCTGGCTCCCTGTAT-GCCATTTGCAAAGCTC
miR160a_line11_col.2_A.tumLBA      GTCGTGTACACGTAT-T-GCCTGGCTCCCTGTA------ATTTGCAAAGCTC
miR160a_line11_col.3_A.tumLBA      GTCGTGTACACGTATAT-GCCTGGCTCCCTGTAT-GCCATTTGCAAAGCTC
miR160a_line11_col.4_A.tumLBA      GTCGTGTACACGTATAT-GCCTGGCTCCCTGTAT-GCCATTTGCAAAGCTC
miR160a_line11_col.5_A.tumLBA      GTCGTGTACACGT-----GCCTGGCTCCCTGT---GCCATTTGAAAAGCTC
miR160a_line11_col.6_A.tumLBA      GTCGTGTACACGTATAT-GCCTGGCTCCCTGTAT-GCCATTTGCAAAGCTC
miR160a_line11_col.7_A.tumLBA      GTCGTGTACACGTATAT-GCCTGGCTCCCTGTAT-GCCATTTGCAAAGCTC
miR160a_line11_col.8_A.tumLBA      GTCGTGTACACGT-----GCCTGGCTCCCTGT---GCCATTTGCAAAGCTC
miR160a_line11_col.9_A.tumLBA      GTCGTGTACACGTAT-T-GCCTGGCTCCCTGTA------ATTTGCAAAGCTC
miR160a_line11_col.10_A.tumLBA     GTCGTGTACACGT-----GCCTGGCTCCCTGTAT------TTGCAAAGCTC
miR160a_line11_col.11_A.tumLBA     GTCGTGTACACGTATAT-GCCTGGCTCCCTGTAT-GCCATTTACAAAGCTC

miR160a_line5_col.1_A.tumC58       GTCGTGTACACGTATAT-GCCTGGCTCCCTGTAT-GCCATTTGCAAAGCTC
miR160a_line5_col.2_A.tumC58       GTCGTGTACACGTATAT-GCCTGGCTCCCTGTAT-GCCATTTGCAAAGCTC
miR160a_line5_col.3_A.tumC58       GTCGTGTACACGTATAT-GCCTGGCTCCCTGTAT-GCCATTTGCAAAGCTC
miR160a_line5_col.4_A.tumC58       GTCGTGTACACGTATAT-GCCTGGCTCCCTGTAT-GCCATTTGCAAAGCTC
miR160a_line5_col.5_A.tumC58       GTCGTGTACACGTATAT-GCCTGGCTCCCTGTAT-GCCATTTGCAAAGCTC
miR160a_line5_col.6_A.tumC58       GTCGTGTACACGTATAT-GCCTGGCTCCCTGTAT-GCCATTTGCAAAGCTC
miR160a_line5_col.7_A.tumC85       GTCGTGTACACGTATAT-GCCTGGCTCCCTGTAT-GCCATTTGCAAAGCTC
miR160a_line5_col.8_A.tumC58       GTCGTGTACACGTATAT-GCCTGGCTCCCTGTAT-GCCATTTGCAAAGCTC
miR160a_line5_col.9_A.tumC58       GTCGTGTACACGTATAT-GCCTGGCTCCCTGTAT-GCCATTTGCAAAGCTC
miR160a_line5_col.10_A.tumC58      GTCGTGTACACGTATAT-GCCTGGCTCCCTGTAT-GCCATTTGCAAAGCTC

miR160a_line7_col.1_A.tumC58       GTCGCGTACACGTAT---GCGTGGCTCCCTGTAT---CATTTGCAAAGCTC
miR160a_line7_col.2_A.tumC58       GTCGTGTACACGTATAT-GCCTGGCTCCCTGTAT-GCCATTTGCAAAGCTC
miR160a_line7_col.3_A.tumC58       GTCGTGTACACGTATAT-GCCTGGCTCCCTGTAT-GCCATTTGCAAAGCTC
miR160a_line7_col.5_A.tumC58       GTCGTGTACACGTATAT-GCCTGGCTCCCTGTAT-GCCATTTGCAAAGCTC
miR160a_line7_col.6_A.tumC58       GTCGTGTACACGTATATTGCCTGGCTCCCTGTA--GCCATTTGCAAAGCTC
miR160a_line7_col.7_A.tumC58       GTCGTGTACACGTATAT-GCCTGGCTCCCTGTAT-GCCATTTGCAAAGCTC
miR160a_line7_col.8_A.tumC58       GTCGTGTACACGTATAT-GCCTGGCTCCCTGTAT-GCCATTTGCAAAGCTC
miR160a_line7_col.9_A.tumC58       GTCGTGTACACGTATAT-GCCTGGCTCCCTGTAT-GCCATTTGCAAAGCTC
miR160a_line7_col.10_A.tumC58      GTCGTGTACACGTATAT-GCCTGGCTCCCTGTAT-GCCATTTGCAAAGCTC
miR160a_line7_col.11_A.tumC58      GTCGTGTACACGTAT---GCCTGGCTCCCTGTAT---CATTTGCAAAGCTC

miR160a_line8_col.3_A.tumC58       GTCGTGTACACGTATAT-GCCTGGCTCCCTGTAT-GCCATTTGCAAAGCTC
miR160a_line8_col.4_A.tumC58       GTCGTGTACACGTATAT-GCCTGGCTCCCTGTAT-GCCATTTGCAAAGCTC
miR160a_line8_col.5_A.tumC58       GTCGTGTACACGTATAT-GCCTGGCTCCCTGTAT-GCCATTTGCAAAGCTC
miR160a_line8_col.6_A.tumC58       GTCGTGTACACGTATAT-GCCTGGCTCCCTGTAT-GCCATTTGCAAAGCTC
miR160a_line8_col.7_A.tumC58       GTCGTGTACACGTATAT-GCCTGGCTCCCTGTAT-GCCATTTGCAAAGCTC
miR160a_line8_col.10_A.tumC58      GTCGTGTACACGTATAT-GCCTGGCTCCCTGTAT-GCCATTTGCAAAGCTC
miR160a_line8_col.11_A.tumC58      GTCGTGTACACGTATAT-GCCTGGCTCCCTGTAT-GCCATTTGCAAAGCTC

miR160a_line10_col.1_A.tumC58      GTCGTGTACACGTATAT-GCCTGGCTCCCTGTAT-GCCATTTGCAAAGCTC
miR160a_line10_col.2_A.tumC58      GTCGTGTACACGTATAT-GCCTGGCTCCCTGTAT-GCCATTTGCAAAGCTC
miR160a_line10_col.4_A.tumC58      GTCGTGTACACGTATAT-GCCTGGCTCCCTGTAT-GCCATTTGCAAAGCTC
miR160a_line10_col.5_A.tumC58      GTCGTGTACACGTATAT-GCCTGGCTCCCTGTAT-GCCATTTGCAAAGCTC
miR160a_line10_col.6_A.tumC58      GTCGTGTACACGTATAT-GCCTGGCTCCCTGTAT-GCCATTTGCAAAGCTC
miR160a_line10_col.11_A.tumC58     GTCGTGTACACGTATAT-GCCTGGCTCCCTGTAT-GCCATTTGCAAAGCTC

miR160a_line11_col.1_A.tumC58      GTCGTGTACACGTATAT-GCCTGGCTCCCTGTAT-GCCATTTGCAAAGCTC
miR160a_line11_col.2_A.tumC58      GTCGTGTACACGTATAT-GCCTGGCTCCCTGTAT-GCCATTTGCAAAGCTC
miR160a_line11_col.3_A.tumC58      GTCGTGTACACGTATAT-GCCTGGCTCCCTGTAT-GCCATTTGCAAAGCTC
miR160a_line11_col.4_A.tumC58      GTCGTGTACACGTATAT-GCCTGGCTCCCTGTAT-GCCATTTGCAAAGCTC
miR160a_line11_col.5_A.tumC58      GTCGTGTACACGTATAT-GCCTGGCTCCCTGTAT-GCCATTTGCAAAGCTC
miR160a_line11_col.7_A.tumC58      GTCGTGTACACGTATAT-GCCTGGCTCCCTGTAT-GCCATTTGCAAAGCTC
miR160a_line11_col.8_A.tumC58      GTCGTGTACACGTATAT-GCCTGGCTCCCTGTAT-GCCATTTGCAAAGCTC
miR160a_line11_col.9_A.tumC58      GTCGTGTACACGTATAT-GCCTGGCTCCCTGTAT-GCCATTTGCAAAGCTC
miR160a_line11_col.10_A.tumC58     GTCGTGTACACGTATAT-GCCTGGCTCCCTGTAT-GCCATTTGCAAAGCTC
miR160a_line11_col.11_A.tumC58     GTCGTGTACACGTATAT-GCCTGGCTCCCTGTAT-GCCATTTGCAAAGCTC
```

Rywal

```
                                      ▼                  ▼
                                   GTCGTGTACACGTATATGCCTGGCTCCCTGTATGCCATTTGCAAAGCTC

miR160a_NT_col.1                   GTCGTGTACACGTATATGCCTGGCTCCCTGTATGCCATTTGCAAAGCTC
miR160a_NT_col.2                   GTCGTGTACACGTATATGCCTGGCTCCCTGTATGCCATTTGCAAAGCTC
miR160a_NT_col.5                   GTCGTGTACACGTATATGCCTGGCTCCCTGTATGCCATTTGCAAAGCTC
miR160a_NT_col.6                   GTCGTGTACACGTATATGCCTGGCTCCCTGTATGCCATTTGCAAAGCTC
miR160a_NT_col.7                   GTCGTGTACACGTATATGCCTGGCTCCCTGTATGCCATTTGCAAAGCTC
miR160a_NT_col.10                  GTCGTGTACACGTATATGCCTGGCTCCCTGTATGCCATTTGCAAAGCTC
miR160a_NT_col.11                  GTCGTGTACACGTATATGCCTGGCTCCCTGTATGCCATTTGCAAAGCTC
miR160a_NT_col.12                  GTCGTGTACACGTATATGCCTGGCTCCCTGTATGCCATTTGCAAAGCTC
```

miR160a_line8_col.1_A.tumLBA	GTCGTGTACACGTATATGCCTGGCTCCCTGTATGCCATTTGCAAAGCTC
miR160a_line8_col.2_A.tumLBA	GTCGTGTACACGTATATGCCTGGCTCCCTGTATGCCATTTGCAAAGCTC
miR160a_line8_col.4_A.tumLBA	GTCGTGTACACGTATATGCCTGGCTCCCTGTATGCCATTTGCAAAGCTC
miR160a_line8_col.6_A.tumLBA	GTCGTGTACACGTA----CCTGGCTCCCTGTA----ATTTGCAAAGCTC
miR160a_line8_col.7_A.tumLBA	GTCGTGTACACGTATATGCCTGGCTCCCTGTATGCCATTTGCAAAGCTC
miR160a_line8_col.9_A.tumLBA	GTCGTGTACACGTATATGCCTGGCTCCCTGTATGCCATTTGCAAAGCTC
miR160a_line8_col.10_A.tumLBA	GTCGTGTACACGTATATGCCTGGCTCCCTGTATGCCATTTGCAAAGCTC
miR160a_line8_col.11_A.tumLBA	GTCGTGTACACGTATATGCCTGGCTCCCTGTATGCCATTTGCAAAGCTC

miR160a_line9_col.1_A.tumLBA	GTCGTGTACACGTATATGCCTGGCTCCCTGTATGCCATTTGCAAAGCTC
miR160a_line9_col.3_A.tumLBA	GTCGTGTACACGTATATGCCTGGCTCCCTGTATGCCATTTGCAAAGCTC
miR160a_line9_col.6_A.tumLBA	GTCGTGTACACGTATATGCCTGGCTCCCTGTATGCCATTTGCAAAGCTC
miR160a_line9_col.7_A.tumLBA	GTCGTGTACACGTATATGCCTGGCTCCCTGTATGCCATTTGCAAAGCTC
miR160a_line9_col.9_A.tumLBA	GTCGTGTACACGTATATGCCTGGCTCCCTGTATGCCATTTGCAAAGCTC
miR160a_line9_col.10_A.tumLBA	GTCGTGTACACGTATATGCCTGGCTCCCTGTATGCCATTTGCAAAGCTC
miR160a_line9_col.11_A.tumLBA	GTCGTGTACACGTATATGCCTGGCTCCCTGTATGCCATTTGCAAAGCTC

miR160a_line3_col.1_A.tumLBA	GTCGTGTACACGTATATGCCTGGCTCCCTGTATGCCATTTGCAAAGCTC
miR160a_line3_col.2_A.tumLBA	GTCGTGTACACGTATATGCCTGGCTCCCTGTATGCCATTTGCAAAGCTC
miR160a_line3_col.5_A.tumLBA	GTCGTGTACACGTATATGCCTGGCTCCCTGTATGCCATTTGCAAAGCTC
miR160a_line3_col.6_A.tumLBA	GTCGTGTACACGTATATGCCTGGCTCCCTGTATGCCATTTGCAAAGCTC
miR160a_line3_col.8_A.tumLBA	GTCGTGTACACGTATATGCCTGGCTCCCTGTATGCCATTTGCAAAGCTC
miR160a_line3_col.9_A.tumLBA	GTCGTGTACACGTATATGCCTGGCTCCCTGTATGCCATTTGCAAAGCTC
miR160a_line3_col.10_A.tumLBA	GTCGTGTACACGTATATGCCTGGCTCCCTGTATGCCATTTGCAAAGCTC
miR160a_line3_col.11_A.tumLBA	GTCGTGTACACGTATATGCCTGGCTCCCTGTATGCCATTTGCAAAGCTC
miR160a_line3_col.12_A.tumLBA	GTCGTGTACACGTATATGCCTGGCTCCCTGTATGCCATTTGCAAAGCTC

miR160a_line9_col.2_A.tumC58	GTCGTGTACACGTATATGCCTGGCTCCCTGTATGCCATTTGCAAAGCTC
miR160a_line9_col.3_A.tumC58	GTCGTGTACACGTATATGCCTGGCTCCCTGTATGCCATTTGCAAAGCTC
miR160a_line9_col.4_A.tumC58	GTCGTGTACACGTATATGCCTGGCTCCCTGTATGCCATTTGCAAAGCTC
miR160a_line9_col.6_A.tumC58	GTCGTGTACACGTATATGCCTGGCTCCCTGTATGCCATTTGCAAAGCTC
miR160a_line9_col.7_A.tumC58	GTCGTGTACACGTATATGCCTGGCTCCCTGTATGCCATTTGCAAAGCTC
miR160a_line9_col.8_A.tumC58	GTCGTGTACACGTATATGCCTGGCTCCCTGTATGCCATTTGCAAAGCTC
miR160a_line9_col.9_A.tumC58	GTCGTGTACACGTATATGCCTGGCTCCCTGTATGCCATTTGCAAAGCTC
miR160a_line9_col.10_A.tumC58	GTCGTGTACACGTATATGCCTGGCTCCCTGTATGCCATTTGCAAAGCTC
miR160a_line9_col.11_A.tumC58	GTCGTGTACACGTATATGCCTGGCTCCCTGTATGCCATTTGCAAAGCTC

miR160a_line18_col.2_A.tumLBA	GTCGTGTACACGTATATGCCTGGCTCCCTGTATGCCATTTGCAAAGCTC
miR160a_line18_col.3_A.tumLBA	GTCGTGTACACGTATATGCCTGGCTCCCTGTATGCCATTTGCAAAGCTC
miR160a_line18_col.8_A.tumLBA	GTCGTGTACACGTATATGCCTGGCTCCCTGTATGCCATTTGCAAAGCTC
miR160a_line18_col.11_A.tumLBA	GTCGTGTACACGTATATGCCTGGCTCCCTGTATGCCATTTGCAAAGCTC
miR160a_line18_col.12_A.tumLBA	GTCGTGTACACGTATATGCCTGGCTCCCTGTATGCCATTTGCAAAGCTC
miR160a_line18_col.13_A.tumLBA	GTCGTGTACACGTATATGCCTGGCTCCCTGTATGCCATTTGCAAAGCTC
miR160a_line18_col.14_A.tumLBA	GTCGTGTACACGTATATGCCTGGCTCCCTGTATGCCATTTGCAAAGCTC

miR160a_line19_col.2_A.tumLBA	GTCGTGTACACGTATATGCCTGGCTCCCTGTATGCCATTTGCAAAGCTC
miR160a_line19_col.8_A.tumLBA	GTCGTGTACACGTATATGCCTGGCTCCCTGTATGCCATTTGCAAAGCTC
miR160a_line19_col.9_A.tumLBA	GTCGTGTACACGTATATGCCTGGCTCCCTGTATGCCATTTGCAAAGCTC
miR160a_line19_col.11_A.tumLBA	GTCGTGTACACGTATATGCCTGGCTCCCTGTATGCCATTTGCAAAGCTC
miR160a_line19_col.12_A.tumLBA	GTCGTGTACACGTATATGCCTGGCTCCCTGTATGCCATTTGCAAAGCTC
miR160a_line19_col.18_A.tumLBA	GTCGTGTACACGTATATGCCTGGCTCCCTGTATGCCATTTGCAAAGCTC
miR160a_line19_col.19_A.tumLBA	GTCGTGTACACGTATATGCCTGGCTCCCTGTATGCCATTTGCAAAGCTC

miR160a_line20_col.1_A.tumLBA	GTCGTGTACACGTATATGCCTGGCTCCCTGTATGCCATTTGCAAAGCTC
miR160a_line20_col.3_A.tumLBA	GTCGTGTACACGTATATGCCTGGCTCCCTGTATGCCATTTGCAAAGCTC
miR160a_line20_col.5_A.tumLBA	GTCGTGTACACGTATATGCCTGGCTCCCTGTATGCCATTTGCAAAGCTC
miR160a_line20_col.6_A.tumLBA	GTCGTGTACACGTATATGCCTGGCTCCCTGTATGCCATTTGCAAAGCTC
miR160a_line20_col.7_A.tumLBA	GTCGTGTACACGTATATGCCTGGCTCCCTGTATGCCATTTGCAAAGCTC
miR160a_line20_col.13_A.tumLBA	GTCGTGTACACGTATATGCCTGGCTCCCTGTATGCCATTTGCAAAGCTC
miR160a_line20_col.18_A.tumLBA	GTCGTGTACACGTATATGCCTGGCTCCCTGTATGCCATTTGCAAAGCTC
miR160a_line20_col.19_A.tumLBA	GTCGTGTACACGTATATGCCTGGCTCCCTGTATGCCATTTGCAAAGCTC

b) *MIR160b*

Désirée

	AGGAGTAAGAATGATG-TGCCTGGCTCCCTGTAT-GCCACACACTTTCACC
miR160b_NT_col.1	AGGAGTAAGAATGATG-TGCCTGGCTCCCTGTAT-GCCACACACTTTCACC
miR160b_NT_col.2	AGGAGTAAGAATGGTG-TGCCTGGCTCCCTGTAT-GCCACACACTTTCACC
miR160b_NT_col.3	AGGAGTAAGAATGATG-TGCCTGGCTCCCTGTAT-GCCACACACTTTCACC
miR160b_NT_col.4	AGGAGTAAGAATGATG-TGCCTGGCTCCCTGTAT-GCCACACACTTTCACC
miR160b_NT_col.5	AGGAGTAAGAATGATG-TGCCTGGCTCCCTGTAT-GCCACACACTTTCACC
miR160b_NT_col.6	AGGAGTAAGAATGATG-TGCCTGGCTCCCTGTAT-GCCACACACTTTCACC
miR160b_NT_col.7	AGGAGTAAGAATGATG-TGCCTGGCTCCCTGTAT-GCCACACACTTTCACC
miR160b_NT_col.8	AGGAGTAAGAATGGTG-TGCCTGGCTCCCTGTAT-GCCACACACTTTCACC
miR160b_NT_col.9	AGGAGTAAGAATGGTG-TGCCTGGCTCCCTGTAT-GCCACACACTTTCACC
miR160b_NT_col.10	AGGAGTAAGAATGATG-TGCCTGGCTCCCTGTAT-GCCACACACTTTCACC
miR160b_NT-proto_col.1	AGGAGTAAGAATGATG-TGCCTGGCTCCCTGTAT-GCCACACACTTTCACC
miR160b_NT-proto_col.2	AGGAGTAAGAATGATG-TGCCTGGCTCCCTGTAT-GCCACACACTTTCACC
miR160b_NT-proto_col.3	AGGAGTAAGAATGATG-TGCCTGGCTCCCTGTAT-GCCACACACTTTCACC
miR160b_NT-proto_col.4	AGGAGTAAGAATGATG-TGCCTGGCTCCCTGTAT-GCCACACACTTTCACC
miR160b_NT-proto_col.5	AGGAGTAAGAATGATG-TGCCTGGCTCCCTGTAT-GCCACACACTTTCACC
miR160b_NT-proto_col.6	AGGAGTAAGAATGATG-TGCCTGGCTCCCTGTAT-GCCACACACTTTCACC
miR160b_NT-proto_col.7	AGGAGTAAGAATGGTG-TGCCTGGCTCCCTGTAT-GCCACACACTTTCACC
miR160b_NT-proto_col.8	AGGAGTAAGAATGGTG-TGCCTGGCTCCCTGTAT-GCCACACACTTTCACC
miR160b_NT-proto_col.9	AGGAGTAAGAATGATG-TGCCTGGCTCCCTGTAT-GCCACACACTTTCACC
miR160b_NT-proto_col.10	AGGAGTAAGAATGATG-TGCCTGGCTCCCTGTAT-GCCACACACTTTCACC
miR160b_line1_col.1_A.tumLBA	AGGAGTAAGAATGGTG-TGCCTGGCTCCC------GCCACACACTTTCACC
miR160b_line1_col.2_A.tumLBA	AGGAGTAAGAATGATG---CCTGGCTCCCTGTATTGCCACACACTTTCACC
miR160b_line1_col.3_A.tumLBA	AGGAGTAAGAATGGTG-TGCCTGGCTCCCTGTATTGCCACACACTTTCACC
miR160b_line1_col.4_A.tumLBA	AGGAGTAAGAATGATG---CCTGGCTCCCTG----------CACTTTCACC
miR160b_line1_col.5_A.tumLBA	AGGAGTAAGAATGGTG-TGCCTGGCTCCCTGTATTGCCACACACTTTCACC
miR160b_line1_col.6_A.tumLBA	AGGAGTAAGAATG------CCTGGCTCCCTGTA--GCCACACACTTTCACC
miR160b_line1_col.7_A.tumLBA	AGGAGTAAGAATGATG---CCTGGCTCCCTG-------------CTTTCACC
miR160b_line1_col.8_A.tumLBA	AGGAGTAAGAATGATGTTGCCTGGCTCCCTGTATTGCCACACACTTTCACC
miR160b_line1_col.9_A.tumLBA	-------------------CCTGGCTCCCTGTATTGCCACACACTTTCACC
miR160b_line1_col.10_A.tumLBA	AGGAGTAAGA-------TGCCTGGCTCCCTGTA------CACACTTTCACC
miR160b_line4_col.1_A.tumLBA	AGGAGTAAGAATGGTG-TGCCTGGCTCCCTGTAT-GCCACACACTTTCACC
miR160b_line4_col.2_A.tumLBA	AGGAGTAAGAATGATG-TGCCTGGCTCCCTGTAT-GCCACACACTTTCACC
miR160b_line4_col.3_A.tumLBA	AGGAGTAAGAATGGTG-TGCCTGGCTCCCTGTAT-GCCACACACTTTCACC
miR160b_line4_col.4_A.tumLBA	AGGAGTAAGAATGATG-TGCCTGGCTCCCTGTAT-GCCACACACTTTCACC
miR160b_line4_col.5_A.tumLBA	AGGAGTAAGAATGATG-TGCCTGGCTCCCTGTAT-GCCACACACTTTCACC
miR160b_line4_col.6_A.tumLBA	AGGAGTAAGAATGATG-TGCCTGGCTCCCTGTAT-GCCACACACTTTCACC
miR160b_line4_col.7_A.tumLBA	AGGAGTAAGAATGGTG-TGCCTGGCTCCCTGTAT-GCCACACACTTTCACC
miR160b_line4_col.8_A.tumLBA	AGGAGTAAGAATGGTG-TGCCTGGCTCCCTGTAT-GCCACACACTTTCACC
miR160b_line4_col.9_A.tumLBA	AGGAGTAAGAATGGTG-TGCCTGGCTCCCTGTAT-GCCACACACTTTCACC
miR160b_line4_col.10_A.tumLBA	AGGAGTAAGAATGATG-TGCCTGGCTCCCTGTAT-GCCACACACTTTCACC
miR160b_line4_col.11_A.tumLBA	AGGAGTAAGAATGGTG-TGCCTGGCTCCCTGTAT-GCCACACACTTTCACC
miR160b_line6_col.1_A.tumC58	AGGAGTAAGAATGGTG-TGCCTGGCTCC--------------ACTTTCACC
miR160b_line6_col.2_A.tumC58	AGGAGTAAGAATGATG-TGCCTGGCTCCCTGTAT-GCCACACACTTTCACC
miR160b_line6_col.3_A.tumC58	AGGAGT------------GCCTGGCTCCCTGT--------ACACTTTCACC
miR160b_line6_col.4_A.tumC58	AGGAGT------------GCCTGGCTCCCTGT--------ACACTTTCACC
miR160b_line6_col.6_A.tumC58	AGGAGTAAGAATGGTG-TGCCTGGCTCCCTGTAT-GCCACACACTTTCACC
miR160b_line6_col.12_A.tumC58	AGGAGT------------GCCTGGCTCCCTGT--------ACACTTTCACC
miR160b_line6_col.13_A.tumC58	AGGAGTAAGAATGATG-TGCCTGGCTCCCTGTA---CCACACACTTTCACC
miR160b_line6_col.14_A.tumC58	AGGAGTAAGAATGGTG-TGCCTGGCTCCCTGTAT-GCCACACACTTTCACC
miR160b_line6_col.15_A.tumC58	AGGAGTAAGA--------GCCTGGCTCCCTGTAT-GCCACACACTTTCACC
miR160b_line8_col.1_A.tumC58	AGGAGTAAGAATGATG-TGCCTGGCTCCCTGTAT-GCCACACACTTTCACC
miR160b_line8_col.2_A.tumC58	AGGAGTAAGAATGGTG-TGCCTGGCTCCCTGTAT-GCCACACACTTTCACC
miR160b_line8_col.3_A.tumC58	AGGAGTAAGAATGATG-TGCCTGGCTCCCTGTAT-GCCACACACTTTCACC
miR160b_line8_col.4_A.tumC58	AGGAGTAAGAATGATG-TGCCTGGCTCCCTGTAT-GCCACACACTTTCACC
miR160b_line8_col.5_A.tumC58	AGGAGTAAGAATGATG-TGCCTGGCTCCCTGTAT-GCCACACACTTTCACC
miR160b_line8_col.6_A.tumC58	AGGAGTAAGAATGATG-TGCCTGGCTCCCTGTAT-GCCACACACTTTCACC
miR160b_line8_col.7_A.tumC58	AGGAGTAAGAATGATG-TGCCTGGCTCCCTGTAT-GCCACACACTTTCACC
miR160b_line8_col.8_A.tumC58	AGGAGTAAGAATGGTG-TGCCTGGCTCCCTGTAT-GCCACACACTTTCACC
miR160b_line8_col.11_A.tumC58	AGGAGTAAGAATGATG-TGCCTGGCTCCCTGTAT-GCCACACACTTTCACC
miR160b_line8_col.12_A.tumC58	AGGAGTAAGAATGATG-TGCCTGGCTCCCTGTAT-GCCACACACTTTCACC

```
miR160b_line12_col.1_A.tumC58        AGGAGTAAGAATGATG-TGCCTGGCTCCCTGTAT-GCCACACACTTTCACC
miR160b_line12_col.2_A.tumC58        AGGAGTAAGAATGGTG-TGCCTGGCTCCCTGTAT-GCCACACACTTTCACC
miR160b_line12_col.3_A.tumC58        AGGAGTAAGAATGATG-TGCCTGGCTCCCTGTAT-GCCACACACTTTCACC
miR160b_line12_col.4_A.tumC58        AGGAGTAAGAATGATG-TGCCTGGCTCCCTGTAT-GCCACACACTTTCACC
miR160b_line12_col.5_A.tumC58        AGGAGTAAGAATGATG-TGCCTGGCTCCCTGTAT-GCCACACACTTTCACC
miR160b_line12_col.6_A.tumC58        AGGAGTAAGAATGATG-TGCCTGGCTCCCTGTAT-GCCACACACTTTCACC
miR160b_line12_col.7_A.tumC58        AGGAGTAAGAATGATG-TGCCTGGCTCCCTGTAT-GCCACACACTTTCACC
miR160b_line12_col.8_A.tumC58        AGGAGTAAGAATGATG-TGCCTGGCTCCCTGTAT-GCCACACACTTTCACC
miR160b_line12_col.9_A.tumC58        AGGAGTAAGAATGATG-TGCCTGGCTCCCTGTAT-GCCACACACTTTCACC
miR160b_line12_col.10_A.tumC58       AGGAGTAAGAATGATG-TGCCTGGCTCCCTGTAT-GCCACACACTTTCACC
```

c) *MIR390a*

Désirée

```
                                   ATGGAGAATCTGTAAAG-CTCAGGAGGGATAGCGCCATGGATGATTCAATT-GATCTG

miR390a_NT_col.1                   ATGGAGAATCTGTAAAG-CTCAGGAGGGATAGCGCCATGGATGACTCAATTTGATCTT
miR390a_NT_col.2                   ATGGAGAATCTGTAAAG-CTCAGGAGGGATAGCGCCATGGATGACTCAATT-GATCTT
miR390a_NT_col.3                   ATGGAGAATCTGTAAAG-CTCAGGAGGGATAGCGCCATGGATGACTCAATTTGATCTT
miR390a_NT_col.6                   ATGGAGAATCTGTAAAG-CTCAGGAGGGATAGCGCCATGGATGACTCAATT-GATCTT
miR390a_NT_col.8                   ATGGAGAATCTGTAAAG-CTCAGGAGGGATAGCGCCATGGATGACTCAATTTGATCTT
miR390a_NT_col.9                   ATGGAGAATCTGTAAAG-CTCAGGAGGGATAGCGCCATGGATGACTCAATTTGATCTT
miR390a_NT_col.10                  ATGGAGAATCTGTAAAG-CTCAGGAGGGATAGCGCCATGGATGACTCAATTTGATCTT
miR390a_NT_col.11                  ATGGAGAATCTGTAAAG-CTCAGGAGGGATAGCGCCATGGATGACTCAATTTGATCTT
miR390a_NT_col.12                  ATGGAGAATCTGTAAAG-CTCAGGAGGGATAGCGCCATGGATGACTCAATT-GATCTT

miR390a_NT-proto_col.1             ATGGAGAATCTGTAAAG-CTCAGGAGGGATAGCGCCATGGATGACTCAATTTGATCTT
miR390a_NT-proto_col.2             ATGGAGAATCTGTAAAG-CTCAGGAGGGATAGCGCCATGGATGACTCAATTTGATCTT
miR390a_NT-proto_col.3             ATGGAGAATCTGTAAAG-CTCAGGAGGGATAGCGCCATGGATGACTCAATT-GATCTT
miR390a_NT-proto_col.4             ATGGAGAATCTGTAAAG-CTCAGGAGGGATAGCGCCATGGATGACTCAATT-GATCTT
miR390a_NT-proto_col.5             ATGGAGAATCTGTAAAG-CTCAGGAGGGATAGCGCCATGGATGACTCAATT-GATCTT
miR390a_NT-proto_col.6             ATGGAGAATCTGTAAAG-CTCAGGAGGGATAGCGCCATGGATGACTCAATT-GATCTT
miR390a_NT-proto_col.7             ATGGAGAATCTGTAAAG-CTCAGGAGGGATAGCGCCATGGATGACTCAATTTGATCTT
miR390a_NT-proto_col.8             ATGGAGAATCTGTAAAG-CTCAGGAGGGATAGCGCCATGGATGACTCAATTTGATCTT
miR390a_NT-proto_col.9             ATGGAGAATCTGTAAAG-CTCAGGAGGGATAGCGCCATGGATGACTCAATT-GATCTT
miR390a_NT-proto_col.10            ATGGAGAATCTGTAAAG-CTCAGGAGGGATAGCGCCATGGATGACTCAATT-GATCTT

miR390a_line1_col.8_A.tumC58       ATGGAGAATCTG------CTCAGGAGGGATAGCGCCATGGATGACTCAATTTGATCTT
miR390a_line1_col.9_A.tumC58       ATGGAGAATCTG------CTCAGGAGGGATAGCGCCATGGATGACTCAATTTGATCTT
miR390a_line1_col.14_A.tumC58      ATGGAGAATCTG------CTCAGGAGGGATAGCGCCATGGATGACTCAATTTGATCTT
miR390a_line1_col.15_A.tumC58      ATGGAGAATCTG------CTCAGGAGGGATAGCGCCATGGATGACTCAATTTGATCTT
miR390a_line1_col.16_A.tumC58      ATGGAGAATCTG------CTCAGGAGGGATAGCGCCATGGATGACTCAATTTGATCTT
miR390a_line1_col.18_A.tumC58      ATGGAGAATCTGTA----CTCAGGAGGGATAGCGCCATGGATGACTCAATTTGATCTT
miR390a_line1_col.19_A.tumC58      ATGGAGAATCTG------CTCAGGAGGGATAGCGCCATGGATGGCTCAATTTGATCTT
miR390a_line1_col.20_A.tumC58      ATGGAGAATCTGTA--G-CTCAGGAGGGATAGCGCCATGGATGACTCAATT-GATCTT
miR390a_line1_col.21_A.tumC58      ATGGAGAATCTGTAAAGTCTCAGGAGGGATAGCGCCATGGATGACTCAATT-GATCTT
miR390a_line1_col.22_A.tumC58      ATGGAGAATCTG------CTCAGGAGGGATAGCGCCATGGATGACTCAATTTGATCTT
miR390a_line1_col.24_A.tumC58      ATGGAGAATCTG------CTCAGGAGGGATAGCGCCATGGATGACTCAATTTGATCTT
miR390a_line1_col.26_A.tumC58      ATGGAGAATCTGTA----CTCAGGAGGGATAGCGCCATGGATGACTCAATTTGATCTT

miR390a_line3_col.4_A.tumC58       ATGGAGAATCTGTAAAG-CTCAGGAGGGATAGCGCCATGGATGACTCAATTTGATCTT
miR390a_line3_col.7_A.tumC58       ATGGAGAATCTGTAAAG-CTCAGGAGGGATAGCGCCATGGATGACTCAATT-GATCTT
miR390a_line3_col.8_A.tumC58       ATGGAGAATCTGTAAAG-CTCAGGAGGGATAGCGCCATGGATGACTCAATT-GATCTT
miR390a_line3_col.9_A.tumC58       ATGGAGAATCTGTAAAG-CTCAGGAGGGATAGCGCCATGGATGACTCAATTTGATCTT
miR390a_line3_col.10_A.tumC58      ATGGAGAATCTG------CTCAGGAGGGATAGCGCCATGGATGACTCAATTTGATCTT
miR390a_line3_col.12_A.tumC58      ATGGAGAATCTGTAAAG-CTCAGGAGGGATAGCGCCATGGATGACTCAATTTGATCTT

miR390a_line4_col.1_A.tumC58       ATGGAGAATCTGTAAAG-CTCAGGAGGGATAGCGCCATGGATGACTCAATT-GATCTT
miR390a_line4_col.2_A.tumC58       ATGGAGAATCTGTAAAG-CTCAGGAGGGATAGCGCCATGGATGACTCAATT-GATCTT
miR390a_line4_col.3_A.tumC58       ATGGAGAATCTGTAAAG-CTCAGGAGGGATAGCGCCATGGATGACTCAATT-GATCTT
miR390a_line4_col.4_A.tumC58       ATGGAGAATCTGTAAAG-CTCAGGAGGGATAGCGCCATGGATGACTCAATTTGATCTT
miR390a_line4_col.5_A.tumC58       ATGGAGAATCTGTAAAG-CTCAGGAGGGATAGCGCCATGGATGACTCAATTTGATCTT
miR390a_line4_col.6_A.tumC58       ATGGAGAATCTGTAAAG-CTCAGGAGGGATAGCGCCATGGATGACTCAATT-GATCTT
miR390a_line4_col.7_A.tumC58       ATGGAGAATCTGTAAAG-CTCAGGAGGGATAGCGCCATGGATGACTCAATTTGATCTT
miR390a_line4_col.8_A.tumC58       ATGGAGAATCTGTAAAG-CTCAGGAGGGATAGCGCCATGGATGACTCAATT-GATCTT
miR390a_line4_col.17_A.tumC58      ATGGAGAATCTGTAAAG-CTCAGGAGGGATAGCGCCATGGATGACTCAATTTGATCTT
miR390a_line4_col.18_A.tumC58      ATGGAGAATCTGTAAAG-CTCAGGAGGGATAGCGCCATGGATGACTCAATTTGATCTT

miR390a_line7_col.1_A.tumC58       ATGGAGAATCTGTAAAG-CTCAGGAGGGATAGCGCCATGGATGACTCAATTTGATCTT
miR390a_line7_col.2_A.tumC58       ATGGAGAATCTGTAAAG-CTCAGGAGGGATAGCGCCATGGATGACTCAATTTGATCTT
miR390a_line7_col.3_A.tumC58       ATGGAGAATCTGTAAAG-CTCAGGAGGGATAGCGCCATGGATGACTCAATT-GATCTT
miR390a_line7_col.5_A.tumC58       ATGGAGAATCTGTAAAG-CTCAGGAGGGATAGCGCCATGGATGACTCAATTTGATCTT
miR390a_line7_col.6_A.tumC58       ATGGAGAATCTGTAAAG-CTCAGGAGGGATAGCGCCATGGATGACTCAATTTGATCTT
miR390a_line7_col.7_A.tumC58       ATGGAGAATCTGTAAAG-CTCAGGAGGGATAGCGCCATGGATGACTCAATT-GATCTT
miR390a_line7_col.8_A.tumC58       ATGGAGAATCTGTAAAG-CTCAGGAGGGATAGCGCCATGGATGACTCAATTTGATCTT
miR390a_line7_col.9_A.tumC58       ATGGAGAATCTGTAAAG-CTCAGGAGGGATAGCGCCATGGATGACTCAATTTGATCTT
miR390a_line7_col.11_A.tumC58      ATGGAGAATCTGTAAAG-CTCAGGAGGGATAGCGCCATGGATGACTCAATTTGATCTT
miR390a_line7_col.12_A.tumC58      ATGGAGAATCTGTAAAG-CTCAGGAGGGATAGCGCCATGGATGACTCAATT-GATCTT

miR390a_line8_col.1_A.tumC58       ATGGAGAATCTGTAAAG-CTCAGGAGGGATAGCGCCATGGATGACTCAATT-GATCTT
miR390a_line8_col.2_A.tumC58       ATGGAGAATCTGTAAAG-CTCAGGAGGGATAGCGCCATGGATGACTCAATTTGATCTT
miR390a_line8_col.3_A.tumC58       ATGGAGAATCTGTAAAG-CTCAGGAGGGATAGCGCCATGGATGACTCAATTTGATCTT
miR390a_line8_col.4_A.tumC58       ATGGAGAATCTGTAAAG-CTCAGGAGGGATAGCGCCATGGATGACTCAATT-GATCTT
miR390a_line8_col.5_A.tumC58       ATGGAGAATCTGTAAAG-CTCAGGAGGGATAGCGCCATGGATGACTCAATTTGATCTT
miR390a_line8_col.6_A.tumC58       ATGGAGAATCTGTAAAG-CTCAGGAGGGATAGCGCCATGGATGACTCAATTTGATCTT
```

```
miR390a_line8_col.7_A.tumC58      ATGGAGAATCTGTAAAG-CTCAGGAGGGATAGCGCCATGGATGACTCAATT-GATCTT
miR390a_line8_col.8_A.tumC58      ATGGAGAATCTGTAAAG-CTCAGGAGGGATAGCGCCATGGATGACTCAATT-GATCTT
miR390a_line8_col.9_A.tumC58      ATGGAGAATCTGTAAAG-CTCAGGAGGGATAGCGCCATGGATGACTCAATTTGATCTT
miR390a_line8_col.10_A.tumC58     ATGGAGAATCTGTAAAG-CTCAGGAGGGATAGCGCCATGGATGACTCAATTTGATCTT
miR390a_line8_col.11_A.tumC58     ATGGAGAATCTGTAAAG-CTCAGGAGGGATAGCGCCATGGATGACTCAATTTGATCTT

miR390a_line2_col.4_A.tumLBA      ATGGAGAATCTGT-----CTCAGGAGGGATAGCGCCATGGATGACTCAATT-GATCTT
miR390a_line2_col.8_A.tumLBA      ----------------------------------------------------------
miR390a_line2_col.9_A.tumLBA      ATGGAGAATCTGT-----CTCAGGAGGGATAGCGCCATGGATGACTCAATT-GATCTT
miR390a_line2_col.12_A.tumLBA     ATGGAGAATCTGT-------CAGGAGGGATAGCGCCATGGATGACTCAATTTGATCTT
miR390a_line2_col.14_A.tumLBA     ----------------------------------------------------------
miR390a_line2_col.15_A.tumLBA     ----------------------------------------------------------
miR390a_line2_col.18_A.tumLBA     ATGGAGAATCTGT-----CTCAGGAGGGATAGCGCCATGGATGACTCAATT-GATCTT
miR390a_line2_col.19_A.tumLBA     ATGGAGAATCTGT-----CTCAGGAGGGATAGCGCCATGGATGACCCAATT-GATCTT
miR390a_line2_col.20_A.tumLBA     ATGGAGAATCTGTAAAG--TCAGGAGGGATAGCGCCATGGATGACTCAATTTGATCTT
miR390a_line2_col.23_A.tumLBA     ----------------------------------------------------------

miR390a_line3_col.9_A.tumLBA      ATGGAGAATCTGTAAAG-CTCAGGAGGGATAGCGCCATGGATGACTCAATTTGATCTT
miR390a_line3_col.10_A.tumLBA     ATGGAGAATCTGTAAAG-CTCAGGAGGGATAGCGCCATGGATGACTCAATTTGATCTT
miR390a_line3_col.12_A.tumLBA     ATGGAGAATCTGTAAAG-CTCAGGAGGGATAGCGCCATGGATGACTCAATT-GATCTT
miR390a_line3_col.1a_A.tumLBA     ATGGAGAATCTGTAAAG-CTCAGGAGGGATAGCGCCATGGATGACTCAATT-GATCTT
miR390a_line3_col.2a_A.tumLBA     ATGGAGAATCTGTAAAG-CTCAGGAGGGATAGCGCCATGGATGACTCAATTTGATCTT
miR390a_line3_col.3a_A.tumLBA     ATGGAGAATCTGTAAAG-CTCAGGAGGGATAGCGCCATGGATGACTCAATTTGATCTT
miR390a_line3_col.4a_A.tumLBA     ATGGAGAATCTGTAAAG-CTCAGGAGGGATAGCGCCATGGATGACTCAATTTGATCTT
miR390a_line3_col.5a_A.tumLBA     ATGGAGAATCTGTAAAG-CTCAGGAGGGATAGCGCCATGGATGACTCAATTTGATCTT
miR390a_line3_col.7a_A.tumLBA     ATGGAGAATCTGTAAAG-CTCAGGAGGGATAGCGCCATGGATGACTCAATT-GATCTT
miR390a_line3_col.8a_A.tumLBA     ATGGAGAATCTGTAAAG-CTCAGGAGGGATAGCGCCATGGATGACTCAATT-GATCTT

miR390a_line4_col.9_A.tumLBA      ATGGAGAATCTGTAAAGACTCAGGAGGGATAGCGCCATGGATGACTCAATT-GATCTT
miR390a_line4_col.12_A.tumLBA     ATGGAGAATCTGTAAAG--TCAGGAGGGA-----------------------------
miR390a_line4_col.13_A.tumLBA     ATGGAGAATCTGTAAAGACTCAGGAGGGATAGCGCCATGGATGACTCAATT-GATCTT
miR390a_line4_col.14_A.tumLBA     ATGGAGAATCTGTAAA-------GAGGGATAGCGCCATGGATGACTCAATTTGATCTT
miR390a_line4_col.15_A.tumLBA     ATGGAGAATCTGTAAA--CTCAGGAGGGATAGCGCCATGGATGACTCAATTTGATCTT
miR390a_line4_col.16_A.tumLBA     ATGGAGAATCTGTAA---CTCAGGAGGGATAGCGCCATGGATGACTCAATTTGATCTT
miR390a_line4_col.19_A.tumLBA     ATGGAGAATCTGTAAAGACTCAGGAGGGATAGCGCCATGGATGACTCAATT-GATCTT
miR390a_line4_col.20_A.tumLBA     ATGGAGAATCTGTAAA-------GAGGGATAGCGCCATGGATGACTCAATTTGATCTT

miR390a_line6_col.1_A.tumLBA      ATGGAGAATCTGT-----CTCAGGAGGGATAGCGCCATGGATGACTCAATT-GATCTT
miR390a_line6_col.2_A.tumLBA      ATGGAGAATCTGTAA---CTCAGGAGGGATAGCGCCATGGATGACTCAATTTGATCTT
miR390a_line6_col.3_A.tumLBA      ATGGAGAATCTGTAA---CTCAGGAGGGATAGCGCCATGGATGACTCAATTTGATCTT
miR390a_line6_col.4_A.tumLBA      ATGGAGAATCTGT-----CTCAGGAGGGATAGCGCCATGGATGACTCAATT-GATCTT
miR390a_line6_col.6_A.tumLBA      ATGGAGAATCTG-------TCAGGAGGGATAGCGCCATGGATGACTCAATTTGATCTT
miR390a_line6_col.7_A.tumLBA      ATGGAGAATCTGT-----CTCAGGAGGGATAGCGCCATGGATGACTCAATTTGATCTT
miR390a_line6_col.8_A.tumLBA      ATGGAGAATCTG-------TCAGGAGGGATAGCGCCATGGATGACTCAATTTGATCTT
miR390a_line6_col.9_A.tumLBA      ATGGAGAATCTGT-----CTCAGGAGGGATAGCGCCATGGATGACTCAATT-GATCTT
miR390a_line6_col.10_A.tumLBA     ATGGAGAATCTGTAA---CTCAGGAGGGATAGCGCCATGGATGACTCAATTTGATCTT
miR390a_line6_col.12_A.tumLBA     ATGGAGAATCT---------CAGGAGGGATAGCGCCATGGATGACTCAATTTGATCTT

miR390a_line7_col.1_A.tumLBA      ATGGAGAATCTGTAAA--CTCAGGAGGGATAGCGCCATGGATGACTCAATT-GATCTT
miR390a_line7_col.2_A.tumLBA      ATGGAGAATCTGTAAAG---CAGGAGGGA-----------------------------
miR390a_line7_col.3_A.tumLBA      ATGGAGAATCTGTAA---CTCAGGAGGGATAGCGCCATGGATGACTCAATTTGATCTT
miR390a_line7_col.4_A.tumLBA      ATGGAGAATCTGTAAAG-CTCAGGAGGGATAGCGCCATGGATGACTCAATT-GATCTT
miR390a_line7_col.5_A.tumLBA      ATGGAGAATCTGTAA---CTCAGGAGGGATAGCGCCATGGATGACTCAATTTGATCTT
miR390a_line7_col.6_A.tumLBA      ATGGAGAATCTGTAAA--CTCAGGAGGGATAGCGCCATGGATGACTCAATTTGATCTT
miR390a_line7_col.7_A.tumLBA      ATGGAGAATCTGTAAAG---CAGGAGGGA-----------------------------
miR390a_line7_col.8_A.tumLBA      ATGGAGAATCTGTAA---CTCAGGAGGGATAGCGCCATGGATGACTCAATTTGATCTT
miR390a_line7_col.9_A.tumLBA      ATGGAGAATCTGTAAAG---CAGGAGGGA-----------------------------
miR390a_line7_col.10_A.tumLBA     ATGGAGAATCTGTA----CTCAGGAGGGATAGCGCCATGGATGACTCAATT-GATCTT
miR390a_line7_col.11_A.tumLBA     ATGGAGAATCTGTAA---CTCAGGAGGGATAGCGCCATGGATGACTCAATTTGATCTT
```
