# Supplementary material for: CRISPR/Cas9-mediated fine-tuning of miRNA expression in tetraploid potato
Source: Hortic Res. 2022 Jun 30;9:uhac147. doi: 10.1093/hr/uhac147 (PMC9437727; doi:10.1093/hr/uhac147)
Supplement: Web_Material_uhac147 [file web_material_uhac147.zip › Figure S7.pdf]

Figure S7: Mature miRNAs and miRNA variants produced non-mutated *MIR160a* locus (a) or non-mutated *MIR390a* locus (b). Mature miR-5p and miR-3p are shown in boxes, flanking sequence of pre-miRNA precursor is marked in grey, post-transcriptional modifications are highlighted in green.

a) *MIR160a* locus

GTCGTGTACACGTATATGCCTGGCTCCCTGTATGCCATTTGCAAAGCTCACCGTAATATATCGATGGGCCTTGTTGAATGGCGTATGAGGAGCCAAGCATA

ATATGCCTGGCTCCCTGTATG
ATGCCTGGCTCCCTGTATGCCA
ATGCCTGGCTCCCTGTATGCC
TGCCTGGCTCCCTGTATGCCA
TGCCTGGCTCCCTGTATGCC**C**
TGCCTGGCTCCCTGTATGCC**T**
TGCCTGGCTCCCTGTATGCC
TGCCTGGCTCCCTGTATGC
TGCCTGGCTCCCTGTATG**T**
TGCCTGGCTCCCTGTATG
GCTCCCTGTATGCCATTTGC
TATATCGATGGGCCTTGTTGA
ATATCGATGGGCCTTGTTGA
TATCGATGGGCCTTGTTGAATG**T**
ATGGGCCTTGTTGAATGGCGTATG
GCGTATGAGGAGCCAAGCATA
GCGTATGAGGAGCCAAGCA
GCGTATGAGGAGCCAAGC

b) MIR390a locus

GCATGGAGAATCTGTAAAGCTCAGGAGGGATAGCGCCATGGATGATTCAATTGATCTGTTTGCACATCTCTAGCGCTATCCATCCTGAGTTTTACGGCTTTTTCACGC

ATCTGTAAAGCTCAGGAGGGATAGC
TAAAGCTCAGGAGGGATAGCG
AAGCTCAGGAGGGATAGCGCCA
AAGCTCAGGAGGGATAGCGCC
AAGCTCAGGAGGGATAGCG**TC**
AAGCTCAGGAGGGATAGC**AC**
AAGCTCAGGAGGGATAGC**AT**
AAGCTCAGGAGGGATAGC**A**
AAGCTCAGGAGGGATAGC
AGCTCAGGAGGGATAGCGCC
TCAGGAGGGATAGCGCCATGGA**A**
TCAGGAGGGATAGCGCCATGG**T**
TCAGGAGGGATAGCGCCATG
TCAGGAGGGATAGCGCCAT**T**
CAGGAGGGATAGCGCCATGGA**A**

TGCACATCTCTAGCGCTATCCATC
CTAGCGCTATCCATCCTGAGT
CGCTATCCATCCTGAGTTTTACGG
CGCTATCCATCCTGAGTTTTA
CGCTATCCATCCTGAGTTTT
CGCTATCCATCCTGAGTT**CT**
CGCTATCCATCCTGAGTT**CA**
CGCTATCCATCCTGAGTT**CC**
CGCTATCCATCCTGAGTT
CGCTATCCATCCTGAGT
CATCCTGAGTTTTACG
