# Supplementary material for: CRISPR/Cas9-mediated fine-tuning of miRNA expression in tetraploid potato
Source: Hortic Res. 2022 Jun 30;9:uhac147. doi: 10.1093/hr/uhac147 (PMC9437727; doi:10.1093/hr/uhac147)

Figure S8: Mature miRNAs and miRNA variants detected in cr-*MIR160a* and cr-*MIR390a* transgenic lines of cv. Désirée aligned to wild-type pre-miRNA sequences (A, F, L, N, R; considered as wild-type miRNAs/miRNA variants) or aligned to mutated pre-miRNA sequences from modified alleles (B-E, G-K, M, O, P, S-V) considered as new miRNA variants. Mature miR-5p and miR-3p are shown in boxes, flanking sequence of pre-miRNA precursor is marked in grey, the numbers in parentheses denote the number of raw reads for each sequence, asterisks mark the sequences that were uniquely assigned (mapped only to one specific altered pre-miRNAs), post-transcriptional modifications are highlighted in green. On the right side of each pre-miRNA (a-v) is a corresponding secondary structure along with MFEI values of pre-miRNAs and the prediction processing efficiency. When the MFEI values of the pre-miRNAs were below 0.85 (see Methods) or an internal loop (> 5 nt) was detected in the miRNA/miRNA* duplex region, processing was predicted to be impaired. Sequences of miRNAs coding regions are highlighted in colours (miR-5p– orange, miR-3p - blue).

cr-*MIR160a*_line3_A.tumLBA

A) wild-type pre-miR160a

GTCGTGTACACGTATATGCCTGGCTCCCTGTATGCCATTTGCAAAGCTCACCGTAATATATCGATGGGCCTTGTTGAATGGCGTATGAGGAGCCAAGCATA

ATGCCTGGCTCCCTGTATGCCA (2)
ATGCCTGGCTCCCTGTATGCC (3)
TGCCTGGCTCCCTGTATGCCA (20)
TGCCTGGCTCCCTGTATGCC (9)
TGCCTGGCTCCCTGTATG (1)
ATATCGATGGGCCTTGTTGA (2)

GCGTATGAGGAGCCAAGCATA (1)
GCGTATGAGGAGCCAAGC (3)

B) miRNA160a_line3_col.2_A.tumLBA

GTCGTGTACACGTATATTTGCCTGGCTCCCTGTATTTGCAAAGCTCACCGTAATATATCGATGGGCCTTGTTGAATGGCGTATGAGGAGCCAAGCATA

TGCCTGGCTCCCTGTA (1)
TGCCTGGCTCCCTGT (2)

GATGGGCCTTGTTGAAT (1)
GCGTATGAGGAGCCAAGCTT (2)

C) miRNA160a_line3_col.4_A.tumLBA

GTCGTGTACACGTGCCTGGCTCCCTGTATATTTGCAAAGCTCACCGTAATATATCGATGGGCCTTGTTGAATGGCGTATGAGGAGCCAAGCATA

TGCCTGGCTCCCTGTA (1)
TGCCTGGCTCCCTGT (2)

GATGGGCCTTGTTGAAT (1)
GCGTATGAGGAGCCAAGCTT (2)

D) miRNA160a_line3_col.5,6,7,8_A.tumLBA

GTCGTGTACACGTATATTGCCTGGCTCCCTGTATGCCATTTGCAAAGCTCACCGTAATATATCGATGGGCCTTGTTGAATGGCGTATGAGGAGCCAAGCATA

TGCCTGGCTCCCTGTATGCCG (1) *
TGCCTGGCTCCCTGTATGCT (1) *
TGCCTGGCTCCCTGTA (1)
TGCCTGGCTCCCTGT (2)

GATGGGCCTTGTTGAAT (1)
GCGTATGAGGAGCCAAGCTT (2)

E) miRNA160a_line3_col.9_A.tumLBA

GTCGTGTACACGTATATTGCAAATGGCAAAGCTCACCGTAATATATCGATGGGCCTTGTTGAATGGCGTATGAGGAGCCAAGCATA

GATGGGCCTTGTTGAAT (1)
GCGTATGAGGAGCCAAGCTT (2)

a

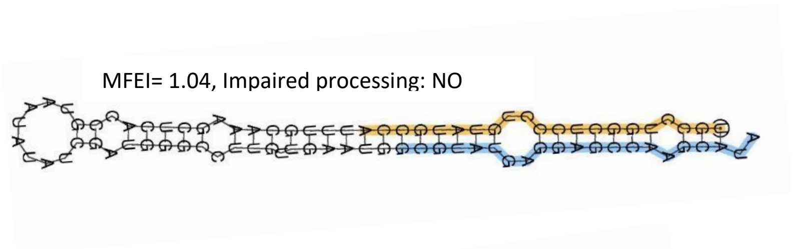

b

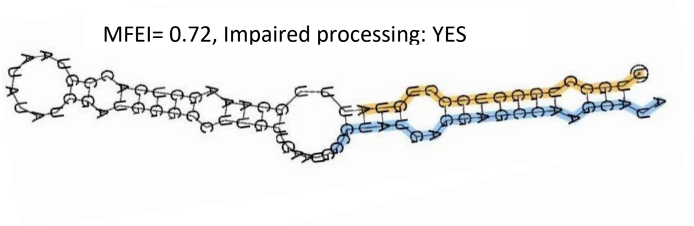

c

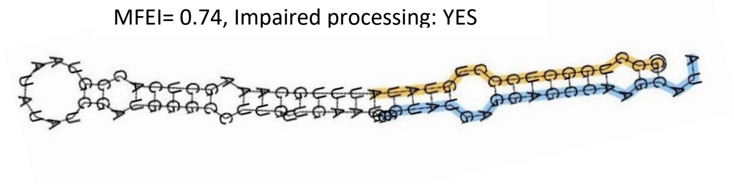

d

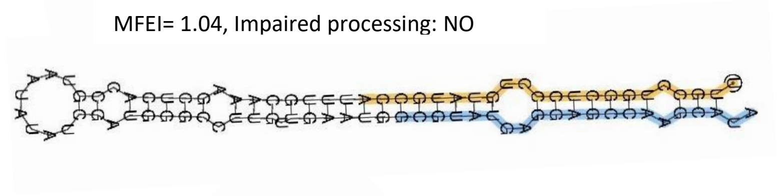

e

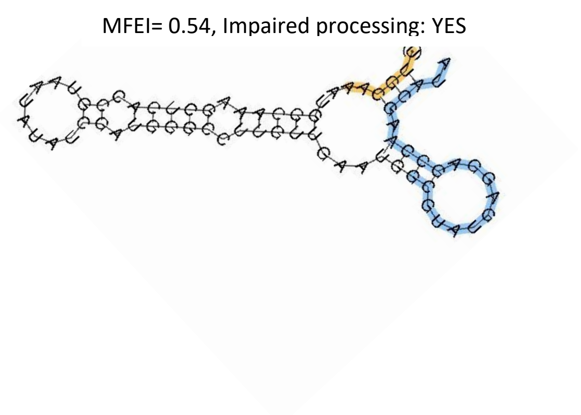

cr-*MIR160a*_line6_A.tumLBA

F)wild-type pre-miR160a

GTCGTGTACACGTATATGCCTGGCTCCCTGTATGCCATTTGCAAAGCTCACCGTAATATATCGATGGGCCTTGTTGAATGGCGTATGAGGAGCCAAGCATA

TGCCTGGCTCCCTGTATGCCA(6)
TGCCTGGCTCCCTGTATGCC(1)

G)miRNA160a_line6_col.1_A.tumLBA

GTCGTGTACACTGCCTGGCTCCCTGTAAAGCTCACCGTAATATATCGATGGGCCTTGTTGAATGGCGTATGAGGAGCCAAGCATA

TGCCTGGCTCCCTGT(1)

H)miRNA160a_line6_col.3_A.tumLBA

GTCGTGTACACGTATTGCCTGGCTCCCTGTATTGCCATTTGCAAAGCTCACCGTAATATATCGATGGGCCTTGTTGAATGGCGTATGAGGAGCCAAGCATA

TGCCTGGCTCCCTGT(1)

I)miRNA160a_line6_col.4_A.tumLBA

GTCGTGTACACGTATTGCCTGGCTCCCTGTAATTTGCAAAGCTCACCGTAATATATCGATGGGCCTTGTTGAATGGCGTATGAGGAGCCAAGCATA

TGCCTGGCTCCCTGT(1)

J)miRNA160a_line6_col.7_A.tumLBA

GTCGTGTACACGTATTGCCTGGCTCCCTGTATGCCATTTGCAAAGCTCACCGTAATATATCGATGGGCCTTGTTGAATGGCGTATGAGGAGCCAAGCATA

TGCCTGGCTCCCTGTATGCCG(3)*
TGCCTGGCTCCCTGT(1)
TGGCTCCCTGTATGCCATTTG(1)*
CGCCTGGCTCCCTGTATGCCA(1)*
CGCCTGGCTCCCTGTATGCC(1)*

K)miRNA160a_line6_col.10_A.tumLBA

GTCGTGTACACGTATATTGCCTGGCTCCCTGTAATTTGCAAAGCTCACCGTAATATATCGATGGGCCTTGTTGAATGGCGTATGAGGAGCCAAGCATA

TGCCTGGCTCCCTGT(1)

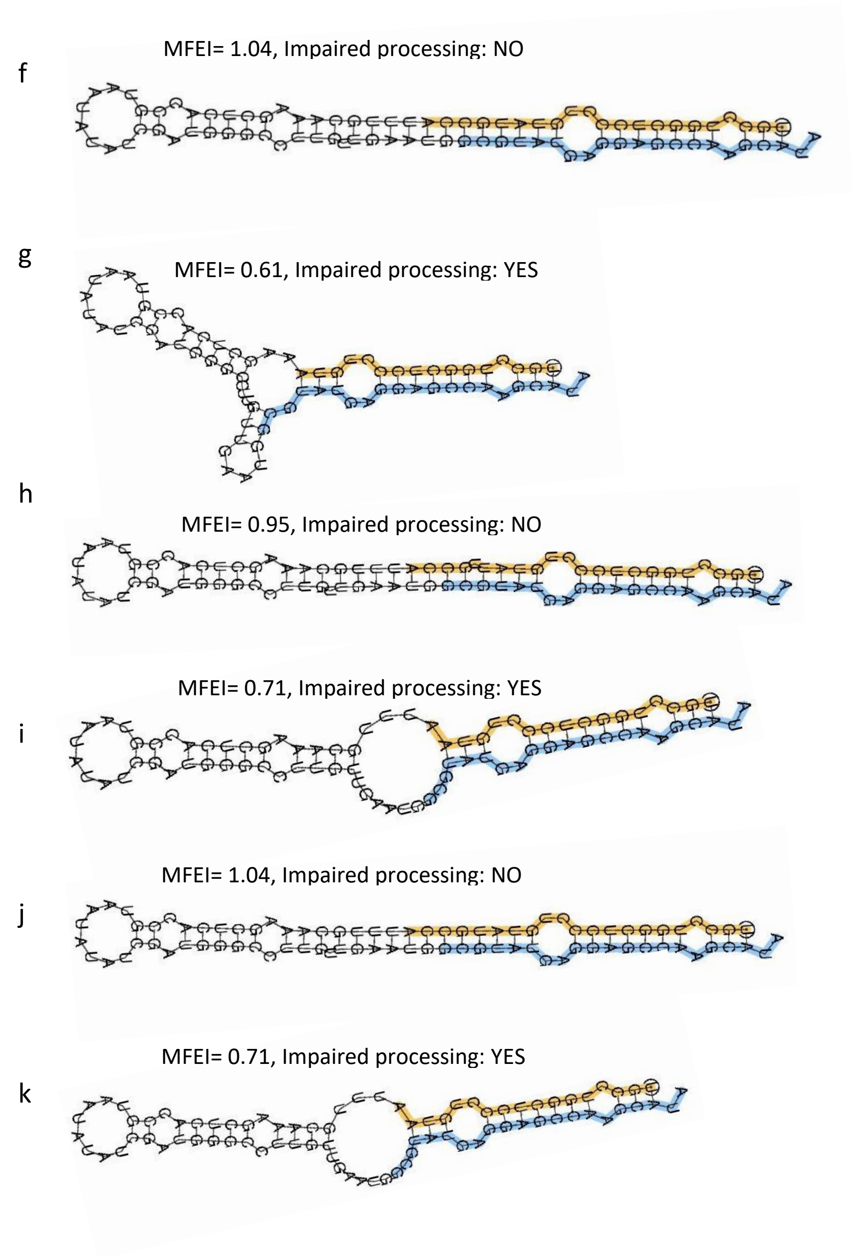

cr-*MIR160*a_line11_A.tumLBA

L) wild-type pre-miR160a

GTCGTGTACACGTATATGCCTGGCTCCCTGTATGCCATTTGCAAAGCTCACCGTAATATATCGATGGGCCTTGTTGAATGGCGTATGAGGAGCCAAGCATA

TGCCTGGCTCCCTGTATGCCA (52)
TGCCTGGCTCCCTGTATGCCC (1)
TGCCTGGCTCCCTGTATGCC (9)
TGCCTGGCTCCCTGTATG (3)

M) miRNA160a_line11_col.11_A.tumLBA

GTCGTGTACACGTATATGCCTGGCTCCCTGTATGCCATTTACAAAGCTCACCGTAATATATCGATGGGCCTTGTTGAATGGCGTATGAGGAGCCAAGCATA

TGCCTGGCTCCCTGTATGCCG (5) *

l

MFEI= 1.04, Impaired processing: NO

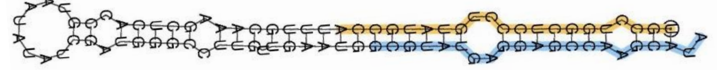

m

MFEI= 1.09, Impaired processing: NO

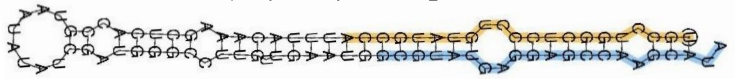

cr-*MIR390a*_line2_A.tumLBA

N)wild-type pre-miR390a

GCATGGAGAATCTGTAAAGCTCAGGAGGGATAGCGCCATGGATGATTCAATTGATCTGTTTGCACATCTCTAGCGCTATCCATCCTGAGTTTTACGGCTTTTTCACGC

TCAGGAGGGATAGCGCCATG(1)

O)miRNA390a_line2_col.4,9,18_A.tumLBA

GCATGGAGAATCTGTCTCAGGAGGGATAGCGCCATGGATGACTCAATTGATCTTTTTGCACATCTCTAGCGCTATCCATCCTGAGTTTTACGGCTTTTTTACGC

ATCCATCCTGAGTTTTACGGCTTT(1)
TTGATCTTTTTGCACATCTCTAGC(1)

P)miRNA390a_line2_col.12_A.tumLBA

GCATGGAGAATCTGTCAGGAGGGATAGCGCCATGGATGACTCAATTTGATCTTTTTGCACATCTCTAGCGCTATCCATCCTGAGTTTTACGGCTTTTTCACGC

ATCCATCCTGAGTTTTACGGCTTT(1)
TTGATCTTTTTGCACATCTCTAGC(1)

Q)miRNA390a_line2_col.20_A.tumLBA

GCATGGAGAATCTGTAAAGTCAGGAGGGATAGCGCCATGGATGACTCAATTTGATCTTTTTGCACATCTCTAGCGCTATCCATCCTGAGTTTTACGGCTTTTTCACGC

ATGGAGAATCTGTAAAG(2)*
ATGGAGAATCTGTAAA(4)*
TTGATCTTTTTGCACATCTCTAGC(1)
ATCCATCCTGAGTTTTACGGCTTT(1)

n

MFEI= 1.04, Impaired processing: NO

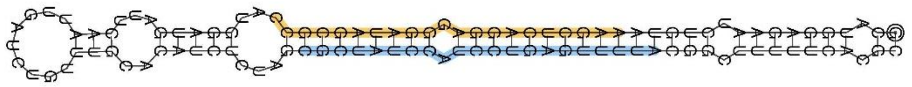

o

MFEI= 0.85, Impaired processing: YES

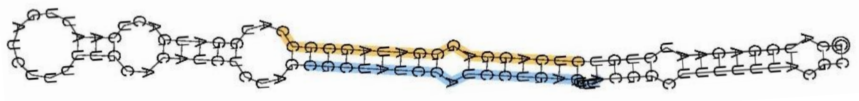

p

MFEI= 0.79, Impaired processing: YES

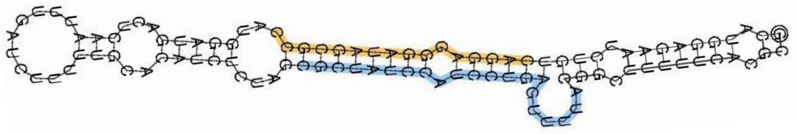

q

MFEI= 0.94, Impaired processing: NO

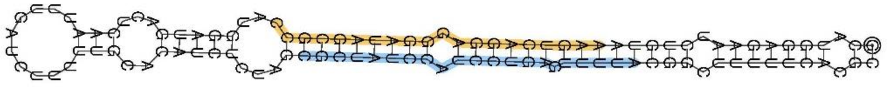

cr-*MIR390a*_line7_A.tumLBA

R)wild-type pre-miR390a

GCATGGAGAATCTGTAAAGCTCAGGAGGGATAGCGCCATGGATGATTCAATTGATCTGTTTGCACATCTCTAGCGCTATCCATCCTGAGTTTTACGGCTTTTTCACGC

AAGCTCAGGAGGGATAGCGCC(1)
AAGCTCAGGAGGGATAGCA(1)
CGCTATCCATCCTGAGTTTT(3)
CGCTATCCATCCTGAGTTCT(1)

S)miRNA390a_line7_col.1_A.tumLBA

GCATGGAGAATCTGTAAACTCAGGAGGGATAGCGCCATGGATGACTCAATTGATCTTTTTGCACATCTCTAGCGCTATCCATCCTGAGTTTTACGGCTTTTTTACGC

ATGGAGAATCTGTAAA(2)
ATGGAGAATCTGTAA(1)
AACTCAGGAGGGATAGCGCC(5)
AACTCAGGAGGGATAGCG(1)
CGCTATCCATCCTGAGTTC(1)
ATCCATCCTGAGTTTTACGGCTTT(1)

T)miRNA390a_line7_col.2_A.tumLBA

GCATGGAGAATCTGTAAAGCAGGAGGGAATACATCCTGAGTTTTACGGCTTTTTTACGC

CGATGGAGAATCTGTAAAGC(1)*
TGATGGAGAATCTGTAAAGC(3)*
GATGGAGAATCTGTAAAGC(23)*
GATGGAGAATCTGTAAAG(2)*
ATGGAGAATCTGTAAAGC(21)*
ATGGAGAATCTGTAAAG(1)*
ATGGAGAATCTGTAAA(2)
ATGGAGAATCTGTAA(1)

U)miRNA390a_line7_col.3_A.tumLBA

GCATGGAGAATCTGTAACTCAGGAGGGATAGCGCCATGGATGACTCAATTTGATCTTTTTGCACATCTCTAGCGCTATCCATCCTGAGTTTTACGGCTTTTTCACGC

ATGGAGAATCTGTAA(1)
AACTCAGGAGGGATAGCGCC(5)
AACTCAGGAGGGATAGCG(1)
CGCTATCCATCCTGAGTTC(1)
ATCCATCCTGAGTTTTACGGCTTT(1)

V)miRNA390a_line7_col.10_A.tumLBA

GCATGGAGAATCTGTACTCAGGAGGGATAGCGCCATGGATGACTCAATTGATCTTTTTGCACATCTCTAGCGCTATCCATCCTGAGTTTTACGGCTTTTTTACGC

CGCTATCCATCCTGAGTTC(1)
ATCCATCCTGAGTTTTACGGCTTT(1)

R

MFEI= 1.04, Impaired processing: NO

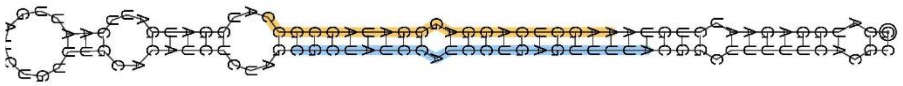

s

MFEI= 0.97, Impaired processing: NO

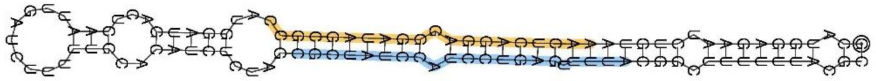

t

MFEI= 0.69, Impaired processing: YES

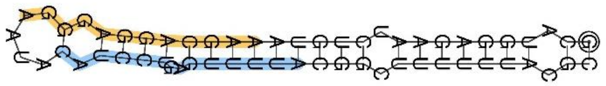

u

MFEI= 0.95, Impaired processing: NO

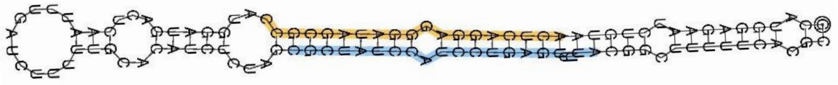

v

MFEI= 0.93, Impaired processing: NO

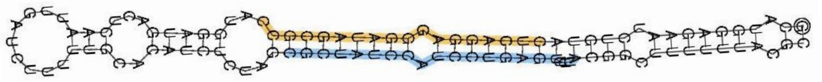

Supplement: Web_Material_uhac147 [file web_material_uhac147.zip › Figure S8.pdf]
