# Supplementary material for: CRISPR/Cas9-mediated fine-tuning of miRNA expression in tetraploid potato
Source: Hortic Res. 2022 Jun 30;9:uhac147. doi: 10.1093/hr/uhac147 (PMC9437727; doi:10.1093/hr/uhac147)
Supplement: Web_Material_uhac147 [file web_material_uhac147.zip › Figure S9.pdf]

Figure S9: Amplification of T-DNA regions from the genomic DNA of transgenic lines. a) The scheme of the T-DNA region integrated in the genome of transgenic plants. Four different T-DNA regions were amplified from the genomic DNA of transgenic plants. Primers target sites and the size of amplicons are shown. The scheme is not to scale. b) Names and sequences of the primers used for amplification of T-DNA regions by PCR. c) Results of agarose gel electrophoresis. PCR amplicons are shown. The primers and the templates (gDNA of transgenic lines) used for amplification are shown above the gel image. LB: left border of T-DNA, RB: right border of T-DNA, PcUBI: *Petroselinum crispum* Ubiquitin4-2 promoter, Kan: kanamycin resistance gene *NptII*, sgRNA1 and sgRNA2: guide sequences, esgRNA: enhanced single guide RNA scaffold, AtU6: RNA PolIII promoter (U6) from *Arabidopsis* (At), StU6: U6 from *Solanum tuberosum* (St), tPea3A: pea3A terminator L: transgenic line. MassRulerDNA Ladder Mix (Thermo Scientific) was used to determine lengths of DNA fragments on agarose gel.

a)

MIR160a

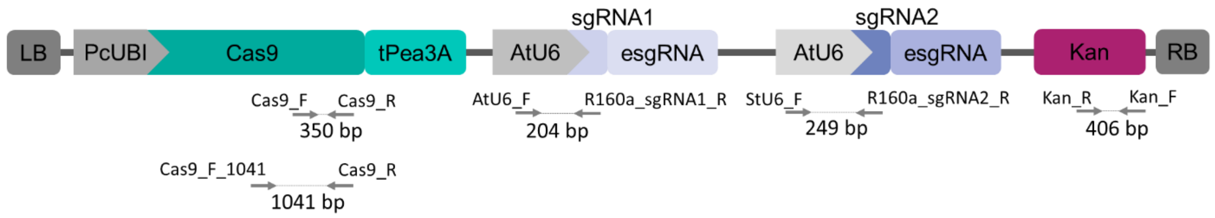

MIR160b

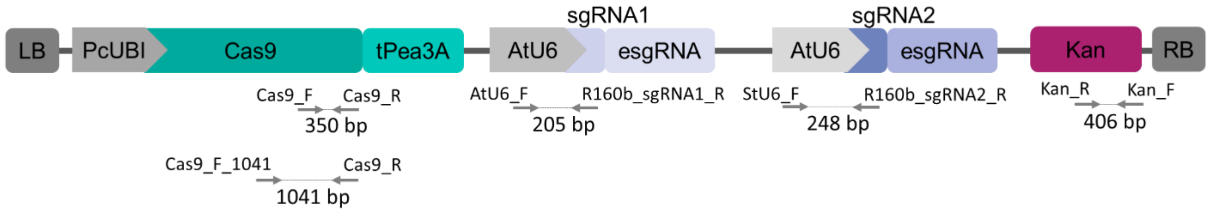

MIR390a

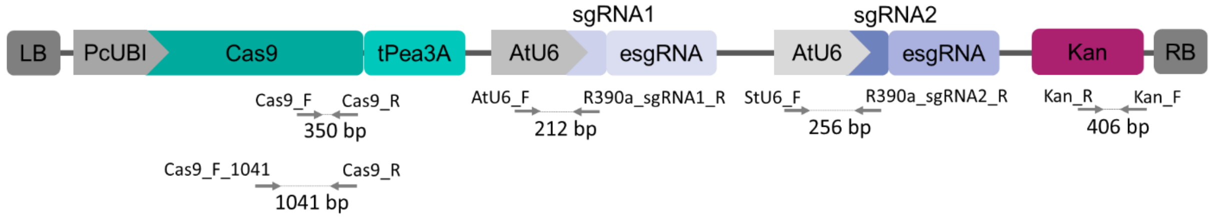

b)

Primer name	Primer sequence (5' → 3')
Cas9_F	ACTTTTCGTTGAGCAGCACAA
Cas9_F_1041	AGCAAGAGATCGGAAAGGCT
Cas9_R	GATCAGCCCTTGAATCACCA
Kan_F	GATGGATTGCACGCAGGTTC
Kan_R	GATGTTTCGCTTGGTGGTCG
AtU6_F	GATAATCTTCAAAAGGCCCCTGG
StU6_F	GAGGTGTGGAATGAAGGATTGTC
R160a_sgRNA1_R	CGGCATATACGTGTACACGAC
R160a_sgRNA2_R	ACGTATGCCATTTGCAAAGCTC
R160b_sgRNA1_R	ACGGCACATCATTCTTACTCCT
R160b_sgRNA2_R	CGTATGCCACACACTTTCACC
R390a_sgRNA1_R	CTTAAACGAGCTTTACAGATTCTCCAT
R390b_sgRNA2_R	CTCTTAAACTGGATGATTCAATTGATCTG

c)

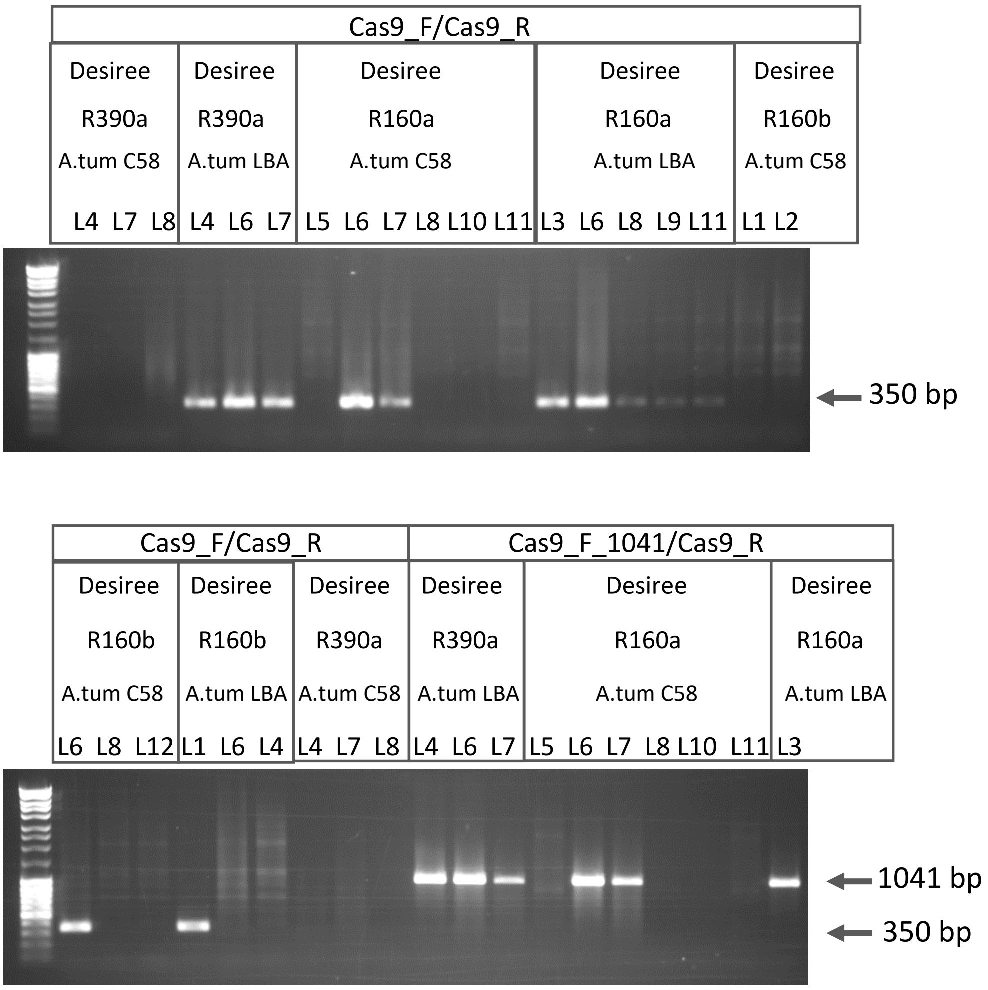

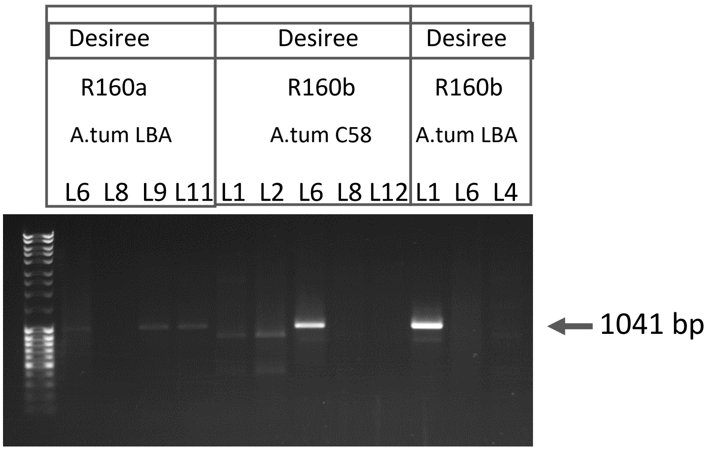
Desiree
R160a
A.tum LBA
L6 L8 L9 L11
Desiree
R160b
A.tum C58
L1 L2 L6 L8 L12
Desiree
R160b
A.tum LBA
L1 L6 L4
1041 bp

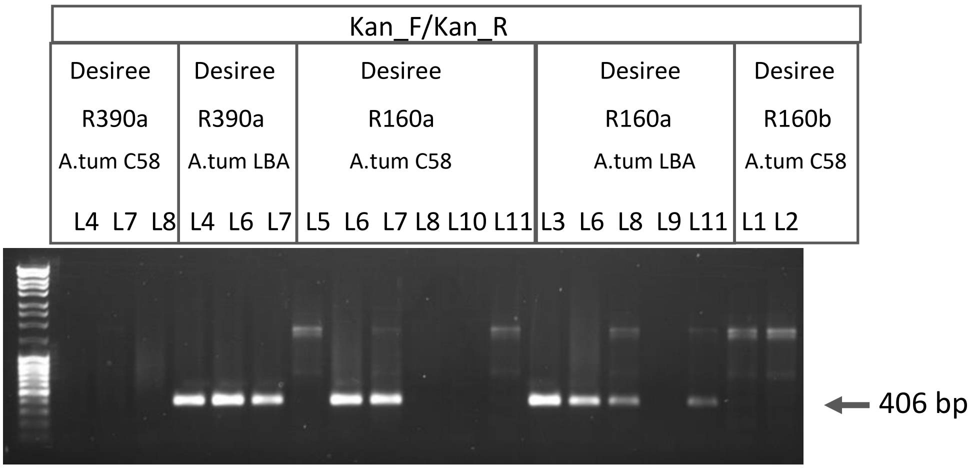
Kan_F/Kan_R
Desiree
R390a
A.tum C58
L4 L7 L8
Desiree
R390a
A.tum LBA
L4 L6 L7
Desiree
R160a
A.tum C58
L5 L6 L7 L8 L10 L11
Desiree
R160a
A.tum LBA
L3 L6 L8 L9 L11
Desiree
R160b
A.tum C58
L1 L2
406 bp

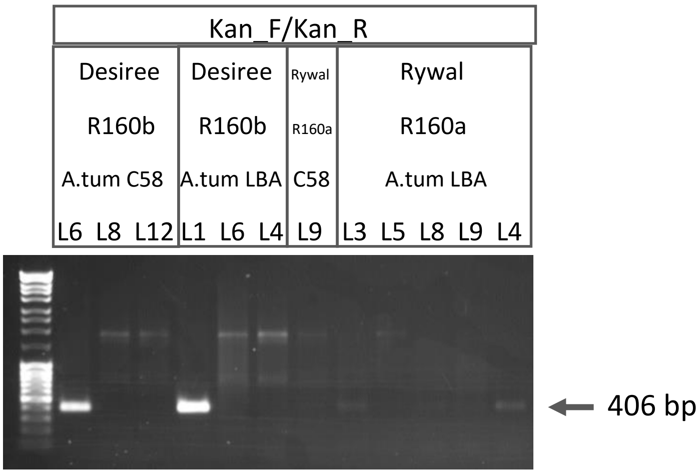
Kan_F/Kan_R
Desiree
R160b
A.tum C58
L6 L8 L12
Desiree
R160b
A.tum LBA
L1 L6 L4
Rywal
R160a
C58
L9
Rywal
R160a
A.tum LBA
L3 L5 L8 L9 L4
406 bp

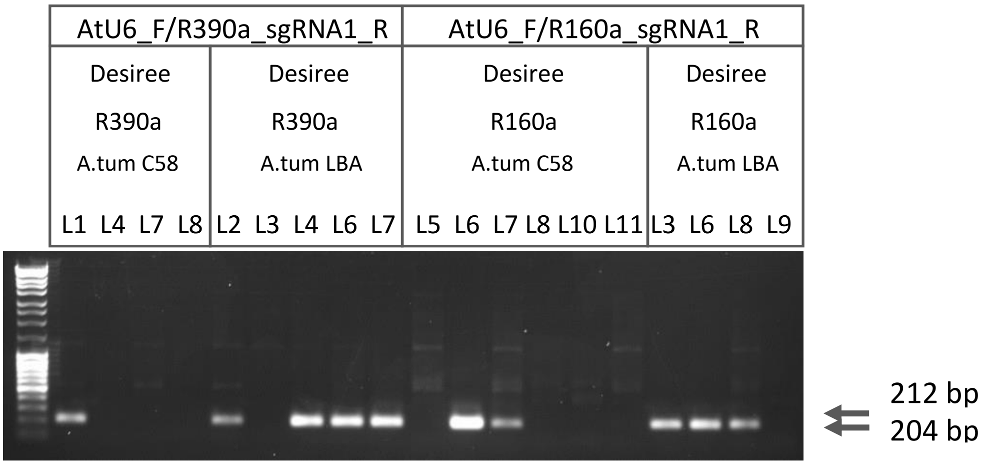
AtU6_F/R390a_sgRNA1_R
AtU6_F/R160a_sgRNA1_R
Desiree R390a A.tum C58
L1 L4 L7 L8
Desiree R390a A.tum LBA
L2 L3 L4 L6 L7
Desiree R160a A.tum C58
L5 L6 L7 L8 L10 L11
Desiree R160a A.tum LBA
L3 L6 L8 L9
212 bp
204 bp

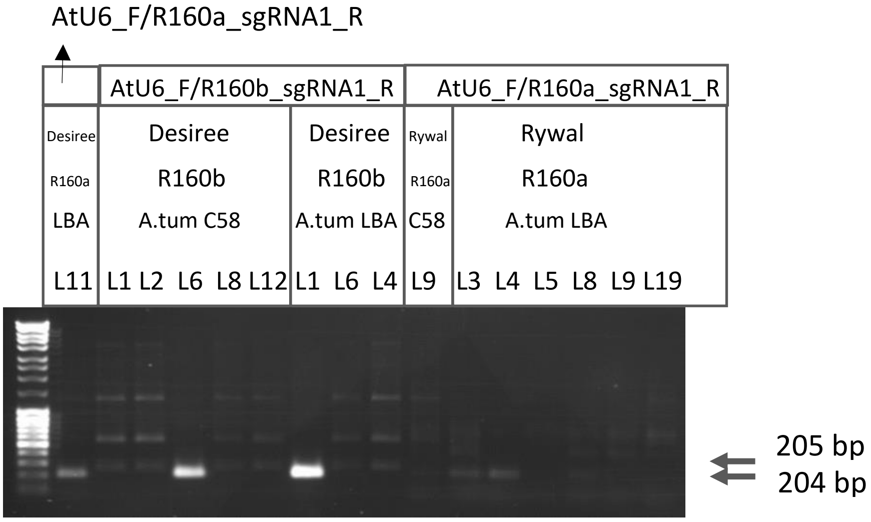
AtU6_F/R160a_sgRNA1_R
AtU6_F/R160b_sgRNA1_R
AtU6_F/R160a_sgRNA1_R
Desiree R160a LBA
L11
Desiree R160b A.tum C58
L1 L2 L6 L8 L12
Desiree R160b A.tum LBA
L1 L6 L4
Rywal R160a C58
L9
Rywal R160a A.tum LBA
L3 L4 L5 L8 L9 L19
205 bp
204 bp

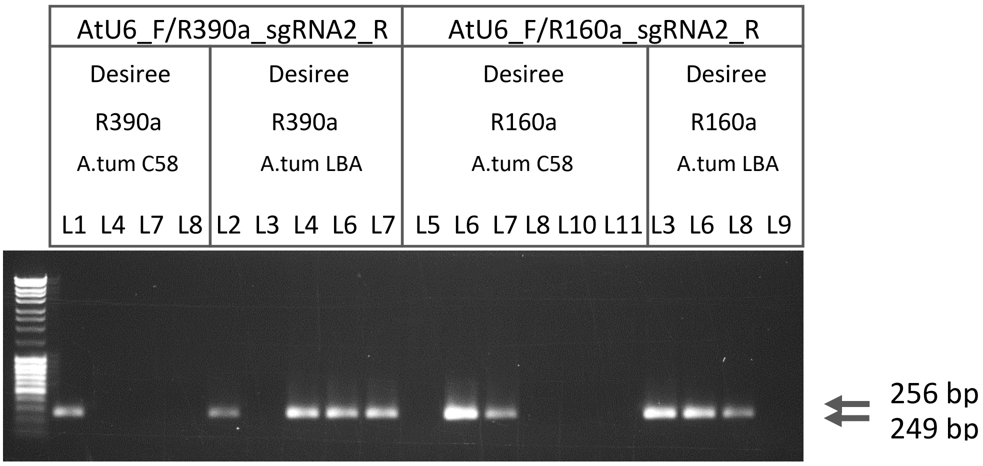
AtU6_F/R390a_sgRNA2_R
AtU6_F/R160a_sgRNA2_R
Desiree R390a A.tum C58
L1 L4 L7 L8
Desiree R390a A.tum LBA
L2 L3 L4 L6 L7
Desiree R160a A.tum C58
L5 L6 L7 L8 L10 L11
Desiree R160a A.tum LBA
L3 L6 L8 L9
256 bp
249 bp

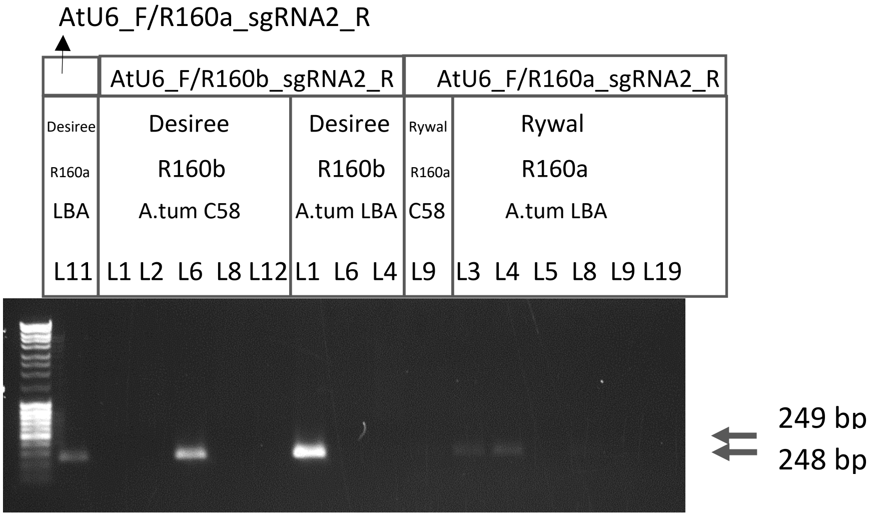
AtU6_F/R160a_sgRNA2_R
AtU6_F/R160b_sgRNA2_R
AtU6_F/R160a_sgRNA2_R
Desiree
R160a
LBA
L11
Desiree
R160b
A.tum C58
L1 L2 L6 L8 L12
Desiree
R160b
A.tum LBA
L1 L6 L4
Rywal
R160a
C58
L9
Rywal
R160a
A.tum LBA
L3 L4 L5 L8 L9 L19
249 bp
248 bp

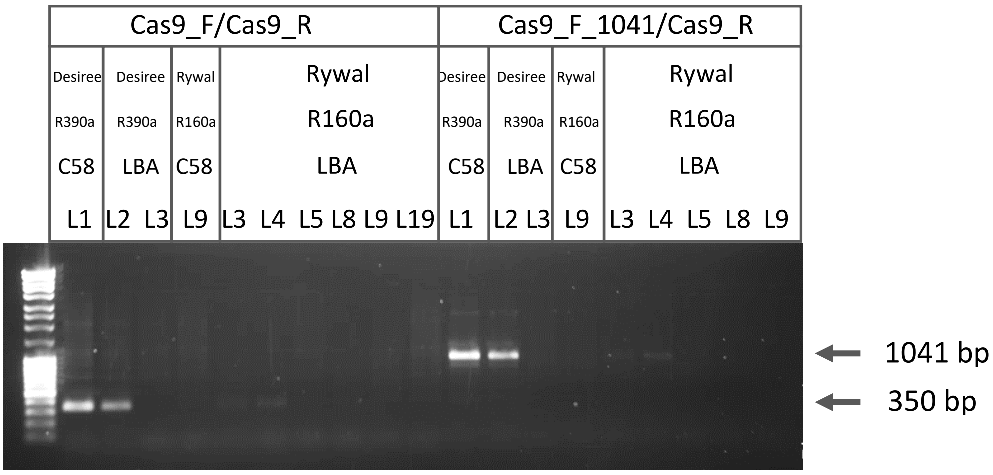
Cas9_F/Cas9_R
Cas9_F_1041/Cas9_R
Desiree
R390a
C58
L1
Desiree
R390a
LBA
L2 L3
Rywal
R160a
C58
L9
Rywal
R160a
LBA
L3 L4 L5 L8 L9 L19
Desiree
R390a
C58
L1
Desiree
R390a
LBA
L2 L3
Rywal
R160a
C58
L9
Rywal
R160a
LBA
L3 L4 L5 L8 L9
1041 bp
350 bp

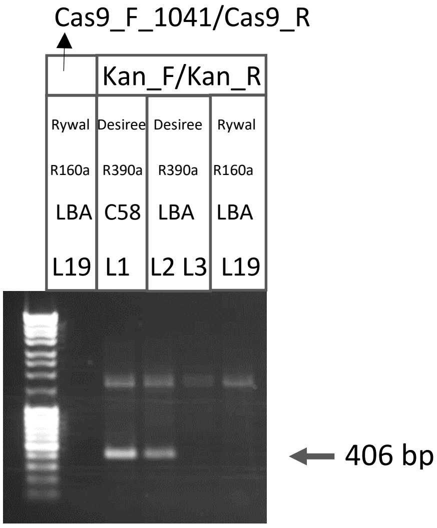
Cas9_F_1041/Cas9_R
Kan_F/Kan_R
Rywal
R160a
LBA
L19
Desiree
R390a
C58
L1
Desiree
R390a
LBA
L2 L3
Rywal
R160a
LBA
L19
406 bp
