# Supplementary material for: CRISPR/Cas9-mediated fine-tuning of miRNA expression in tetraploid potato
Source: Hortic Res. 2022 Jun 30;9:uhac147. doi: 10.1093/hr/uhac147 (PMC9437727; doi:10.1093/hr/uhac147)
Supplement: Web_Material_uhac147 [file web_material_uhac147.zip › Methods S1.pdf]

Methods S1: Precipitation

Ice cold 100% ethanol (2 volumes of final volume) and 3 M potassium acetate (1/10 of the final volume) were added to isolated plasmid DNA. The solution was stored at 4°C for 1 h and centrifuged for 10 min at 17900 g at 4°C. The supernatant was discarded and the pellet was washed with 1 ml of sterile 70% ice cold ethanol. The solution was centrifuged for 10 min at 17900 g at 4°C and the supernatant was discarded. The pellet was kept uncovered at sterile conditions to allow evaporation of residual ethanol and resuspended in sterile ddH_2O to obtain final DNA concentration 1 µg/µl.
