# Supplementary material for: CRISPR/Cas9-mediated fine-tuning of miRNA expression in tetraploid potato
Source: Hortic Res. 2022 Jun 30;9:uhac147. doi: 10.1093/hr/uhac147 (PMC9437727; doi:10.1093/hr/uhac147)
Supplement: Web_Material_uhac147 [file web_material_uhac147.zip › Methods S2.pdf]

Methods S2: *Agrobacterium* transformation

Approximatelly 170 ng of plasmid DNA was added to 30 µl of chemically competent agrobacteria and incubated on ice for 20 min. The mixture was incubated at 42°C for 1,5 min and afterwards incubated on ice for 2 min. After 250 µl of LB medium was added, the mixture was incubated for 1 – 2 h at 28°C and plated on LB plates with selection for the plasmid (Spec) and agrobacteria (Rif and Gen).
