# Supplementary material for: CRISPR/Cas9-mediated fine-tuning of miRNA expression in tetraploid potato
Source: Hortic Res. 2022 Jun 30;9:uhac147. doi: 10.1093/hr/uhac147 (PMC9437727; doi:10.1093/hr/uhac147)
Supplement: Web_Material_uhac147 [file web_material_uhac147.zip › Methods S3.pdf]

Methods S3: Stable transformation Désirée

In the morning on the first day, one bacteria colony was cultured in 3 ml of LB medium with selection, rifampicin (final concentration 20 µg/ml) and spectinomycin (final concentration 75 µg/ml), and grown under standard conditions for agrobacteria. In the afternoon, 500 µl of the morning bacteria culture was inoculated in 3 ml of LB medium with selection and grown overnight.

In the morning on the second day, different volumes (500 and 1000 µl) of overnight bacteria culture were inoculated, each in 50 ml of LB medium with selection. In the afternoon, different volumes (between 50 and 200 µl) of the morning bacteria culture in the exponential growth phase were inoculated, each in 50 ml of LB medium with selection. Bacteria culture was grown overnight.

On the second day, the explants were prepared. First, two sterile filter papers were placed on seven plates with R3B medium and 2 ml of liquid PACM were added afterwards. 500 explants (2-5 mm long internodes) were transferred on five plates (100 on each) and 40 explants were placed on two plates (20 on each). The plates were taped with a parafilm and incubated in a growth chamber overnight.

In the morning on the third day, overnight bacteria culture (OD_{600} = 0.4 - 0.7) was centrifuged for 10 min at 2500 rpm. Pellet was resuspended in 75 ml of LB medium without selection. The upper filterpapers with explants were transferred in the bacteria suspension and were incubated for 5-10 minutes. After transformation, the explants were dried on a sterile filter paper and were transferred on R3B medium. The petridishes were taped with parafilm and incubated in a growth chamber. Two plates with 20 explants were used as a control and were not transformed.

On the fifth day, the explants were transferred onto Zcv medium with the selection, cefotaxime (final concentration 500 mg/l) and kanamycin (final concentration 50 mg/l). The plates were taped with parafilm and incubated in a growth chamber for 14 days. Three plates with Zcv medium were used as controls: the first plate was non-transformed positive control with Zcv medium without kanamycin and without bacteria, the second plate was a positive control with Zcv medium without kanamycin and bacteria, and the third plate was a negative control with Zcv medium with kanamycin and without bacteria. Explants were transferred onto fresh Zcv medium every two weeks. This procedure was repeated until sufficient number of transformants (shoots) were harvested on MS 30 medium with selection.

Media preparations:

For **LB medium**, 10 g of tripton, 5 g of yeast extract, 5 g of Nacl were added in 1 l of bidistilled water, dissolved, calibrated to pH 7.0 and autoclaved.

For **MS 30 medium**, 5 g of Murashige and Skoog Basal Medium with vitamins and 30 g of sucrose were added in 1 l of bidistilled water, dissolved and calibrated to pH 5.8. Next, eight grams of agar were added to the solution and autoclaved.

For **R3B**, 5 g of Murashige and Skoog Basal Medium with vitamins and 30 g of sucrose were added in 1 l of bidistilled water, dissolved and calibrated to pH 5.8. Next, eight grams of agar were added to the solution and autoclaved. Sterile 1-naphthaleneacetic acid (final concentration 2 mg/l) and 6-benzylaminopurine (final concentration 1 mg/l) were added in sterile R3B media.

For **PACM**, 5 g of Murashige and Skoog Basal Medium with vitamins, 2 g of casein hydrolysate and 30 g of sucrose were added in 1 l of bidistilled water, dissolved, calibrated to pH 6.5 and autoclaved.

Sterile 2,4-dichloroacetic acid (final concentration 1 mg/l) and kinetin (final concentration 0.5 mg/l) were added in sterile PACM media.

For **Zcv medium**, 5 g of Murashige and Skoog Basal Medium with vitamins and 20 g of sucrose were added in 1 l of bidistilled water, dissolved and calibrated to pH 5.8. Next, eight grams of agar were added to the solution and autoclaved. Sterile trans-zeatine riboside (final concentration 1 mg/l), cefotaxim (final concentration 200 mg/l), vancomycin (final concentration 200 mg/l) and kanamycin (final concentration 50 mg/l) were added in sterile Zcv media.
