# Supplementary material for: CRISPR/Cas9-mediated fine-tuning of miRNA expression in tetraploid potato
Source: Hortic Res. 2022 Jun 30;9:uhac147. doi: 10.1093/hr/uhac147 (PMC9437727; doi:10.1093/hr/uhac147)
Supplement: Web_Material_uhac147 [file web_material_uhac147.zip › Table S1.pdf]

Table S1: Selected genes and corresponding primers and probes sequences for expression analyses with quantitative PCR. Gene names (abbreviation), IDs and corresponding primer and probe sequences are shown.

**Target gene name**	**Target gene ID/Source**	**Forward primer sequence 5'→3'**	**Reverse primer sequence 5'→3'**	**Probe sequence 5'→3'**
ARF10	Sotub11g026490.1.1	CAGCAGTCCTTTCTGTTGTTTATC[illegible]	CAATCCGGACGGTAAGTTGT	FAM-AACATCACTGCAGGCATACAGGGA-ZEN / Iowa Black FQ
CathB	Sotub02g015280.1.1	ACTGTCTGATCGTTTCTGTATCC	ATCCACCATCACAACCACTC	FAM-ATGATCTGTTAGCGTGCTGTGGCT-ZEN / Iowa Black FQ
Cas9	Streptococcus pyogenes	CAGAAAGCAGAGGACCTTCGA[illegible]	CAATCTTTTCCCTGTTATCCTTGAG	FAM-CTCATCAGATCCACCTCGGAGAGTTGCA-ZEN / Iowa Black FQ
