# Supplementary material for: CRISPR/Cas9-mediated fine-tuning of miRNA expression in tetraploid potato
Source: Hortic Res. 2022 Jun 30;9:uhac147. doi: 10.1093/hr/uhac147 (PMC9437727; doi:10.1093/hr/uhac147)
Supplement: Web_Material_uhac147 [file Figure S5.pdf › Figure S5.pdf]

Figure S5: CRISPR-mediated mutations in miRNAs coding regions and adjacent regions in potato cv. Désirée protoplasts. Results of Sanger sequencing for individual PCR products (col.) covering 5p mature miRNA coding regions of a) *MIR160a*, b) *MIR160b* and c) *MIR390a* coding regions and adjacent regions in protoplasts of non-transgenic potato cv. Désirée (top; NT-proto) and transfected protoplasts (bottom; proto) are shown. Results are presented as consensus sequence, obtained by forward and reverse primers (Figure S11). See Type of mutations sheet in Data S1 for sequencing IDs, primers used and types of mutations). See (https://doi.org/10.5281/zenodo.5727015) for individual sequencing results. miRNA (boxed), sgRNA1 (light grey), sgRNA2 (dark grey), PAM motif (red), theoretical cutting site (arrowhead) in the *MIR* loci are shown above the alignment. Allelic variations are shown by blue, mutations are shown by orange.

a) MIR160a locus

```
                            GTCGTGTACACGTATA                                                                              TGCCTGGCTCCCTGTATGCCATTTGCAAAGCTC

miR160a_NT-proto_col.2      GTCGTGTACACGTATA------------------------------------------------------------------------------TGCCTGGCTCCCTGTATGCCATTTGCAAAGCTC
miR160a_NT-proto_col.3      GTCGTGTACACGTATA------------------------------------------------------------------------------TGCCTGGCTCCCTGTATGCCATTTGCAAAGCTC
miR160a_NT-proto_col.4      GTCGTGTACACGTATA------------------------------------------------------------------------------TGCCTGGCTCCCTGTATGCCATTTGCAAAGCTC
miR160a_NT-proto_col.5      GTCGTGTACACGTATA------------------------------------------------------------------------------TGCCTGGCTCCCTGTATGCCATTTGCAAAGCTC
miR160a_NT-proto_col.6      GTCGTGTACACGTATA------------------------------------------------------------------------------TGCCTGGCTCCCTGTATGCCATTTGCAAAGCTC
miR160a_NT-proto_col.7      GTCGTGTACACGTATA------------------------------------------------------------------------------TGCCTGGCTCCCTGTATGCCATTTGCAAAGCTC
miR160a_NT-proto_col.8      GTCGTGTACACGTATA------------------------------------------------------------------------------TGCCTGGCTCCCTGTATGCCATTTGCAAAGCTC
miR160a_NT-proto_col.9      GTCGTGTACACGTATA------------------------------------------------------------------------------TGCCTGGCTCCCTGTATGCCATTTGCAAAGCTC
miR160a_NT-proto_col.10     GTCGTGTACACGTATA------------------------------------------------------------------------------TGCCTGGCTCCCTGTATGCCATTTGCAAAGCTC

miR160a_proto_col.1         GTCGTGTACACGTATA------------------------------------------------------------------------------TGCCTGGCTCCCTGTATGCCATTTGCAAAGCTC
miR160a_proto_col.3         GTCGTGTACACGTATA------------------------------------------------------------------------------TGCCTGGCTCCCTGTATGCCATTTGCAAAGCTC
miR160a_proto_col.4         GTCGTGTACACGTATA------------------------------------------------------------------------------TGCCTGGCTCCCTGTATGCCATTTGCAAAGCTC
miR160a_proto_col.5         GTCGTGTACACGTATA------------------------------------------------------------------------------TGCCTGGCTCCCTGTATGCCATTTGCAAAGCTC
miR160a_proto_col.6         GTCGTGTACACGTATA------------------------------------------------------------------------------TGCCTGGCTCCCTGTATGCCATTTGCAAAGCTC
miR160a_proto_col.7         GTCGTGTACACGTATA------------------------------------------------------------------------------TGCCTGGCTCCCTGTATGCCATTTGCAAAGCTC
miR160a_proto_col.8         GTCGTGTACACGTATATGTCGCCGTCGCTACGGAGAGGTCGCTACCAAGCGTGGTGACTTGCCCAACGCCACCAGCGTGTTGCGCATCGGCCAGTGCCTGGCTCCCTGTA------TTGCAAAGCTC
miR160a_proto_col.9         GTCGTGTACACGTAT--------------------------------------------------------------------------------GCCTGGCTCCCTGTA-----TTTGCAAAGCTC
miR160a_proto_col.10        GTCGTGTACACGTATA------------------------------------------------------------------------------TGCCTGGCTCCCTGTATGCCATTTGCAAAGCTC
```

b) *MIR160b* locus

```
                           ▼              ▼
                     AGGAGTAAGAATGATGTGCCTGGCTCCCTGTATGCCACACACTTTCACC

miR160b_NT-proto_col.1    AGGAGTAAGAATGATGTGCCTGGCTCCCTGTATGCCACACACTTTCACC
miR160b_NT-proto_col.2    AGGAGTAAGAATGATGTGCCTGGCTCCCTGTATGCCACACACTTTCACC
miR160b_NT-proto_col.3    AGGAGTAAGAATGATGTGCCTGGCTCCCTGTATGCCACACACTTTCACC
miR160b_NT-proto_col.4    AGGAGTAAGAATGATGTGCCTGGCTCCCTGTATGCCACACACTTTCACC
miR160b_NT-proto_col.5    AGGAGTAAGAATGATGTGCCTGGCTCCCTGTATGCCACACACTTTCACC
miR160b_NT-proto_col.6    AGGAGTAAGAATGATGTGCCTGGCTCCCTGTATGCCACACACTTTCACC
miR160b_NT-proto_col.7    AGGAGTAAGAATGGTGTGCCTGGCTCCCTGTATGCCACACACTTTCACC
miR160b_NT-proto_col.8    AGGAGTAAGAATGGTGTGCCTGGCTCCCTGTATGCCACACACTTTCACC
miR160b_NT-proto_col.9    AGGAGTAAGAATGATGTGCCTGGCTCCCTGTATGCCACACACTTTCACC
miR160b_NT-proto_col.10   AGGAGTAAGAATGATGTGCCTGGCTCCCTGTATGCCACACACTTTCACC

miR160b_proto_col.1       AGGAGTAAGAATGGTGTGCCTGGCTCCCTGTATGCCACACACTTTCACC
miR160b_proto_col.2       AGGAGTAAGAATGATGTGCCTGGCTCCCTGTATGCCACACACTTTCACC
miR160b_proto_col.3       AGGAGTAAGAATGGTGTGCCTGGCTCCCTGTATGCCACACACTTTCACC
miR160b_proto_col.4       AGGAGTAAGAATGGTGTGCCTGGCTCCCTGTATGCCACACACTTTCACC
miR160b_proto_col.5       AGGAGTAAGAATGGTGTGCCTGGCTCCCTGTATGCCACACACTTTCACC
miR160b_proto_col.6       AGGAGTAAGAATGATGTGCCTGGCTCCCTGTATGCCACACACTTTCACC
miR160b_proto_col.7       AGGAGTAAGAATGAT--GCCTGGCTCCCTGTA--------------ACC
miR160b_proto_col.8       AGGAGTAAGAATGATGTGCCTGGCTCCCTGTATGCCACACACTTTCACC
miR160b_proto_col.9       AGGAGTAAGAATGGTGTGCCTGGCTCCCTGTATGCCACACACTTTCACC
miR160b_proto_col.10      AGGAGTAAGAATGATGTGCCTGGCTCCCTGTATGCCACACACTTTCACC
```

c) *MIR390a* locus

```
                              ▼                  ▼
                     ATGGAGAATCTGTAAAGCTCAGGAGGGATAGCGCCATGGATGACTCAATT-GATCTG

miR390a_NT-proto_col.1    ATGGAGAATCTGTAAAGCTCAGGAGGGATAGCGCCATGGATGACTCAATTTGATCTT
miR390a_NT-proto_col.2    ATGGAGAATCTGTAAAGCTCAGGAGGGATAGCGCCATGGATGACTCAATTTGATCTT
miR390a_NT-proto_col.3    ATGGAGAATCTGTAAAGCTCAGGAGGGATAGCGCCATGGATGACTCAATT-GATCTT
miR390a_NT-proto_col.4    ATGGAGAATCTGTAAAGCTCAGGAGGGATAGCGCCATGGATGACTCAATT-GATCTT
miR390a_NT-proto_col.5    ATGGAGAATCTGTAAAGCTCAGGAGGGATAGCGCCATGGATGACTCAATT-GATCTT
miR390a_NT-proto_col.6    ATGGAGAATCTGTAAAGCTCAGGAGGGATAGCGCCATGGATGACTCAATT-GATCTT
miR390a_NT-proto_col.7    ATGGAGAATCTGTAAAGCTCAGGAGGGATAGCGCCATGGATGACTCAATTTGATCTT
miR390a_NT-proto_col.8    ATGGAGAATCTGTAAAGCTCAGGAGGGATAGCGCCATGGATGACTCAATTTGATCTT
miR390a_NT-proto_col.9    ATGGAGAATCTGTAAAGCTCAGGAGGGATAGCGCCATGGATGACTCAATT-GATCTT
miR390a_NT-proto_col.10   ATGGAGAATCTGTAAAGCTCAGGAGGGATAGCGCCATGGATGACTCAATT-GATCTT

miR390a_proto_col.1       ATGGAGAATCTGTAAAGCTCAGGAGGGATAGCGCCATGGATGACTCAATTTGATCTT
miR390a_proto_col.2       ATGGAGAATCTGTAAAGCTCAGGAGGGATAGCGCCATGGATGACTCAATT-GATCTT
miR390a_proto_col.3       ATGGAGAATCTGTAAAGCTCAGGAGGGATAGCGCCATGGATGACTCAATTTGATCTT
miR390a_proto_col.4       ATGGAGAATCTGTAAAGCTCAGGAGGGATAGCGCCATGGATGACTCAATT-GATCTT
miR390a_proto_col.5       ATGGAGAATCTGTAAAGCTCAGGAGGGATAGCGCCATGGATGACTCAATT-GATCTT
miR390a_proto_col.6       ATGGAGAATCTGTAAAGCTCAGGAGGGATAGCGCCATGGATGACTCAATT-GATCTT
miR390a_proto_col.7       ATGGAGAATCTGTAAAGCTCAGGAGGGATAGCGCCATGGATGACTCAATT-GATCTT
miR390a_proto_col.8       ATGGAGAATCTGTAAAGCTCAGGAGGGATAGCGCCATGGATGACTCAATT-GATCTT
miR390a_proto_col.9       ATGGAGAATCTGTAAAGCTCAGGAGGGATAGCGCCATGGATGACTCAATTTGATCTT
miR390a_proto_col.10      ATGGAGAATCTGTAAAGCTCAGGAGGGATAGCGCCATGGATGACTCAATT-GATCTT
```
